# Quinone-mediated, tissue-adaptive double-network hydrogel for instant hemostasis and wet-tissue adhesion

**DOI:** 10.1038/s41467-026-72068-6

**Published:** 2026-04-22

**Authors:** Tae Young Kim, Kayoung Son, Chang-Hwan Moon, Keun-Young Yook, Soo A. Kim, Hyein Ham, Yurim Lee, Soo In Lee, Yejin Jo, Yunlong Yu, Dae-Hyun Kim, Jungmok Seo

**Affiliations:** 1 School of Electrical and Electronic Engineering, Yonsei University, Seodaemun-gu, Seoul Republic of Korea; 2 Department of Veterinary Surgery, College of Veterinary Medicine, Gyeongsang National University, Jinju-si Gyeongsangnam-do, Republic of Korea; 3 Institute of Burn Research, Southwest Hospital, Third Military Medical University, Army Medical University, Chongqing, China; 4 Department of Veterinary Surgery, Chungnam National University College of Veterinary Medicine, Daejeon, Republic of Korea

**Keywords:** Biomedical materials, Biomedical materials, Biomedical engineering, Therapeutics, Regenerative medicine

## Abstract

Rapid control of bleeding in complex wounds remains a major clinical challenge. Here, we develop a tissue-adaptive double-network hydrogel that enables instant adhesion to wet tissue and rapid hemostasis. We show that the material can be applied either as an injectable sealant for confined sites or as a conformal patch for large defects. The hydrogel is formed from interpenetrating polymer networks dynamically crosslinked to provide mechanical toughness, rapid self-healing, and shear-thinning injectability. We demonstrate that partial oxidation of catechol groups generates reactive quinone species that form strong bonds with wet tissue without additional activation steps, while incorporated polyphenols accelerate blood clotting. We show that the hydrogel achieves hemostasis within seconds in a mouse liver hemorrhage model and effectively controls bleeding in large-area liver and spleen injuries in rabbits. Finally, we test in a skin incision model to demonstrate that the hydrogel can function as a suture-free adhesive and support healing with minimal scarring.

## Abstract (web summary)

Rapid control of bleeding in complex wounds remains a major clinical challenge. Here, the authors develop a tissue-adaptive hydrogel that enables instant wet-tissue adhesion and rapid hemostasis, demonstrating effective bleeding control in preclinical models.

## Introduction

Rapid control of bleeding and reliable wound sealing without infection are central to surgery and trauma care^1–4^. However, standard closure methods (e.g., clips, staples, and sutures) are invasive, time-consuming to apply, and often do not seal properly or prevent high-flow bleeding^5–8^. Their success depends on operator skill and prolonged handling, which is not ideal in emergency settings^9^. In addition, the standard closure materials can damage soft tissue and trigger a foreign-body reaction (FBR), resulting in inflammation and delayed tissue repair^10–14^. These problems are worse in deep or geometrically irregular wounds where wound closing is particularly difficult^15,16^. These limitations motivate biomaterials-based approaches that combine fast hemostasis with gentle tissue handling.

Conventional hemostatic materials are commonly grouped by mechanism of action as either physical materials or chemical/biochemical materials. Physical materials, including gauze, foam dressings^17–21^, and mechanical closure devices (wound closure Zip band)^22–24^, work by covering the wound, absorbing fluid, and applying local pressure to minimize blood flow. They are generally sufficient for controlling minor or superficial bleeding; however, the absence of intrinsic pro-coagulant activity limits their hemostatic performance. On the other hand, chemical/biochemical hemostatic materials act on biological pathways. Representative examples are alginate^25–30^ and collagen-based dressing^29,31–36^ that form hydrogels upon contact with blood, which facilitate clot formation through platelet activation. Fibrin hydrogel glue promotes fibrin crosslinking and calcium-dependent aggregation^37–40^. In addition to their hemostatic functionality, hydrogel-based materials act as a passive barrier and maintain a moist, pro-healing environment^38,41,42^. Still, trade-offs remain according to their formulation type. Preformed patch-type hydrogel dressings can rapidly cover and adhere to large-area wounds. However, they conform poorly to deep, irregular, or anatomically complex wounds, limiting their clinical versatility^43–45^. By contrast, liquid state glue-type hydrogels are injectable and can reach difficult-to-reach sites, but they are prone to mechanical disruption or washout before adequate clot formation and tissue integration^46,47^.

To address these challenges, shear-thinning double-network (DN) hydrogels have emerged as promising alternatives^48^. The DN architecture offers mechanical toughness, enabling the formation of a stable passivation layer and allowing fabrication into patches of various sizes suitable for large-area wounds^49^. In addition, they are solid-like at rest, but their viscosity decreases under shear stress, allowing injection into deep, irregular cavities^50^. When the shear stress is removed, viscoelastic recovery and dynamic bond re-formation rapidly rebuild a cohesive gel^51^. Shear-thinning facilitates placement, whereas viscoelasticity mediates subsequent stress relaxation^52^. This combination enables the material to wet and fill tissue microtopography during placement and to preserve conformal contact as tissues deform, yielding a tissue-adaptive sealing behavior suited to complex wound environments^53,54^. Despite the advantages, conventional shear-thinning DN hydrogels typically exhibit low intrinsic adhesiveness to biological tissues and are prone to detachment^55–57^. To enhance adhesion, a carbodiimide activation step, such as EDC/NHS (1-ethyl-3-(3-dimethylaminopropyl)carbodiimide/N-hydroxysuccinimide), is commonly employed to facilitate covalent bonds with tissue surfaces^58^. However, this chemical activation strategy poses several practical limitations, including the generation of cytotoxic byproducts, poor long-term stability in vivo, and a lengthy preparation time ( > 1 h). The requirement for this extra step reduces its suitability for time-critical applications, such as trauma management or emergency surgery.

Herein, we present a Strong Tissue-adhesive And Thrombostatic (STAT) DN hydrogel designed to allow instant tissue adhesion through the transition of catechol to o-quinone, thereby allowing direct application onto bleeding wet tissues without a carbodiimide activation step. Moreover, it exhibits versatile formability, rapid hemostasis, and antibacterial properties that are advantageous in diverse wound conditions. The hydrogel is constructed as an interpenetrating polymer network (IPN) comprising two complementary networks. The first network formed by poly(vinyl alcohol) (PVA), sodium alginate functionalized with dopamine (SA-DOPA), tannic acid (TA), and sodium tetraborate decahydrate (borax), provides high mechanical toughness, resistance to external stress, and self-healing capability. The second network, composed of interpenetrated poly(acrylic acid) (PAA), imparts elasticity and flexibility to the hydrogel, supporting long-term structural integrity in dynamic physiological conditions. Notably, SA-DOPA is incorporated to introduce catechol functional groups. During gel formation, these catechols are oxidized into o-quinone structures without any reagents, a process induced simply by brief gloved kneading. This transformation enables strong and immediate tissue adhesion even on wet surfaces without additional chemical treatment. In parallel, TA, a natural polyphenol with multiple hydroxyl groups, enhances platelet aggregation at bleeding sites and chelates membrane-bound metal ions in bacteria, providing both hemostatic and antimicrobial effects.

The hemostatic efficacy of the STAT hydrogel was initially validated in a mouse liver puncture model, where patch application achieved rapid hemostasis within 10 s. Following these results, we subsequently evaluated its performance in rabbit spleen and liver incision/dissection models that simulate massive high-pressure bleeding. In rabbit models, the hydrogel achieved rapid hemostasis and maintained stable adhesion under clinically relevant settings. Furthermore, we employed the STAT hydrogel as a sealant for the incisional skin wound, where it effectively stabilized the wound edges. This moist environment further promoted tissue regeneration with minimal scarring. Collectively, these findings demonstrate that the STAT hydrogel integrates instantaneous wet-tissue adhesion, mechanical resilience, injectability, and antibacterial functionality into a single materials platform. This combination of properties addresses both structural and functional limitations of conventional hemostatic hydrogels, offering a versatile and clinically relevant solution for effective wound management.

## Results

### Design, mechanism, and functional advantages of the STAT hydrogel

**Fig. 1:**
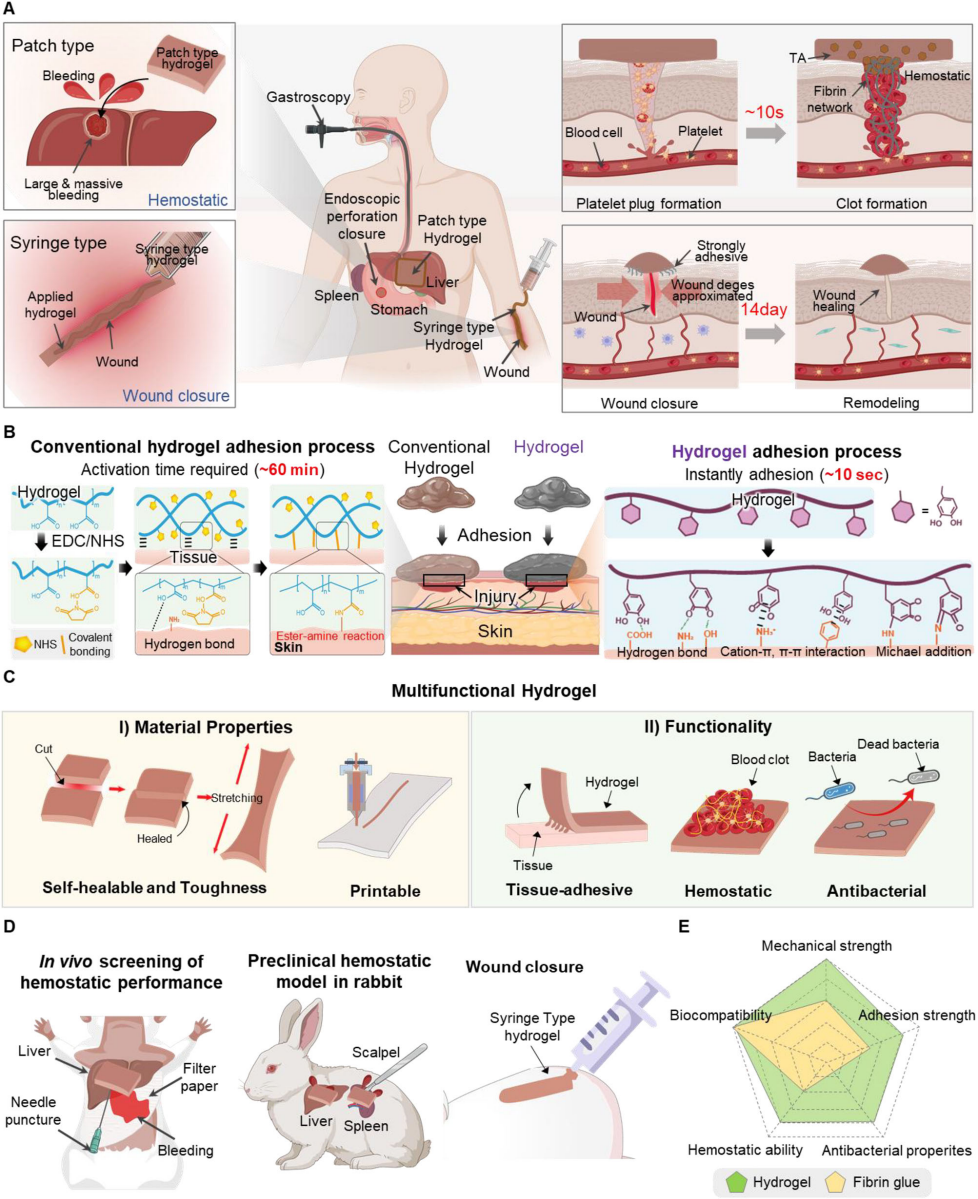
Design, mechanism, and functional advantages of the STAT hydrogel. **A** Schematic illustrations of patch-type and syringe-type STAT hydrogels for hemostasis, endoscopic perforation closure, and wound healing. Created in BioRender. Park, K. (2026). https://BioRender.com/y3jedza**B** Comparison of adhesion process between conventional and the STAT hydrogel. Created in BioRender. Park, K. (2026). https://BioRender.com/y3jedza and created using Adobe Illustrator. **C** The multifunctional properties of the STAT hydrogel. (i) material properties such as self-healing, toughness, and printability, and (ii) key functionalities including tissue adhesion, hemostasis, and antibacterial activity. Created using Adobe Illustrator. **D** Schematic illustration of animal models demonstrating excellent hemostatic and wound closure performance. Created in BioRender. Park, K. (2026). https://BioRender.com/y3jedza and created using Adobe Illustrator. **E** Performance analysis of the STAT hydrogel compared to commercial fibrin glue.

We fabricated two delivery types of the developed STAT hydrogel: a patch and a syringe-type (Fig. 1A). The patch-type can be applied directly to sites of extensive and massive bleeding, enabling rapid coverage and compression of the injury site. In contrast, the syringe-type leverages the hydrogel’s shear-thinning property. Under applied shear stress, the hydrogel’s viscosity decreases, allowing extrusion through a narrow nozzle and uniform application across irregular wound surfaces. Once applied, the STAT hydrogel fills gaps and conforms closely to the wound topography, and physically approximates the separated tissue edges. This mechanical approximation mimics the function of sutures by maintaining alignment of wound margins during the critical early stages of healing, but without the additional trauma caused by needle punctures. The upper-right panel of Fig. 1A illustrates how one of the main components, TA, interacts with endogenous hemostatic factors such as fibrin and platelets. This interaction concentrates these factors at the bleeding site^59^. This biochemical interaction enables rapid clot formation and hemostasis within seconds. The lower panel depicts how the hydrogel can secure gaping tissue and maintain a moist wound environment, which is known to facilitate natural tissue regeneration and accelerate the wound-healing process. Conventional hydrogel sealants often require chemical activators such as EDC/NHS to form covalent bonds with tissue. This process takes > 1 h and carries a risk of local irritation from residual reagents (Fig. 1B). In contrast, the incorporation of SA-DOPA in our STAT hydrogel enables the instantaneous formation of multiple non-covalent and covalent interactions. These include hydrogen bonding, cation–π interactions, π–π stacking, and Michael addition directly with the tissue surface^60^. Through these mechanisms, the STAT hydrogel achieves robust adhesion within seconds without any pretreatment. This rapid bonding is particularly advantageous in emergency or high-volume bleeding scenarios. Thus, although both TA and SA-DOPA contain catechol moieties, they serve distinct and complementary functions in the STAT hydrogel: TA primarily contributes to rapid hemostasis through interactions with endogenous clotting factors, whereas SA-DOPA provides strong interfacial adhesion to wet tissues through catechol-mediated surface interactions. Figure 1C shows illustrations that the STAT hydrogel exhibits high tensile strength due to its DN structure, where the first rigid network dissipates stress through sacrificial bond breakage and the second ductile network maintains structural integrity^61^. The material also possesses self-healing capability arising from dynamic catechol–metal coordination bonds and reversible hydrogen bonding. These mechanisms allow fractured interfaces to re-form both physical and covalent interactions upon contact^62^. Furthermore, the material’s shear-thinning behavior is driven by reversible non-covalent interactions between its polymer chains. Under shear stress, these interactions temporarily break, which lowers the viscosity for easy extrusion. Once the stress is removed, they rapidly re-establish, allowing the printed construct to retain its designed shape. Additionally, the STAT hydrogel demonstrates excellent hemostatic efficacy. This is driven by TA-mediated platelet activation and fibrin network formation. Furthermore, the STAT hydrogel possesses intrinsic antibacterial activity, which is attributed to two factors: the phenolic hydroxyl groups in TA and a slightly acidic microenvironment. Together, these factors compromise bacterial membrane integrity and inhibit bacterial proliferation. Based on these properties, we evaluated in vivo hemostatic performance using a mouse liver puncture model as an initial test. Subsequently, a rabbit spleen incision and liver dissection bleeding model was employed to confirm efficacy under higher blood pressure and severe hemorrhage (Fig. 1D). Finally, the syringe-type STAT hydrogel was applied to the wound region, confirming its potential to function as a suture-free wound closure and healing-promoting material. We performed a comparative analysis of our hydrogel against an FDA-approved commercial fibrin glue (Fig. 1E). While both materials demonstrated comparable biocompatibility, our STAT hydrogel significantly outperformed the fibrin glue in key metrics: mechanical strength, adhesion, antibacterial activity, and hemostatic speed. These results highlight the hydrogel’s potential as a next-generation platform for wound treatment. To further contextualize the performance of the STAT hydrogel, we conducted a comparative analysis with representative recently reported hemostatic adhesive hydrogels based on adhesion strength, hemostatic time, and printability (Supplementary Table 1).

### Fabrication and structural characterization of the STAT hydrogel

**Fig. 2:**
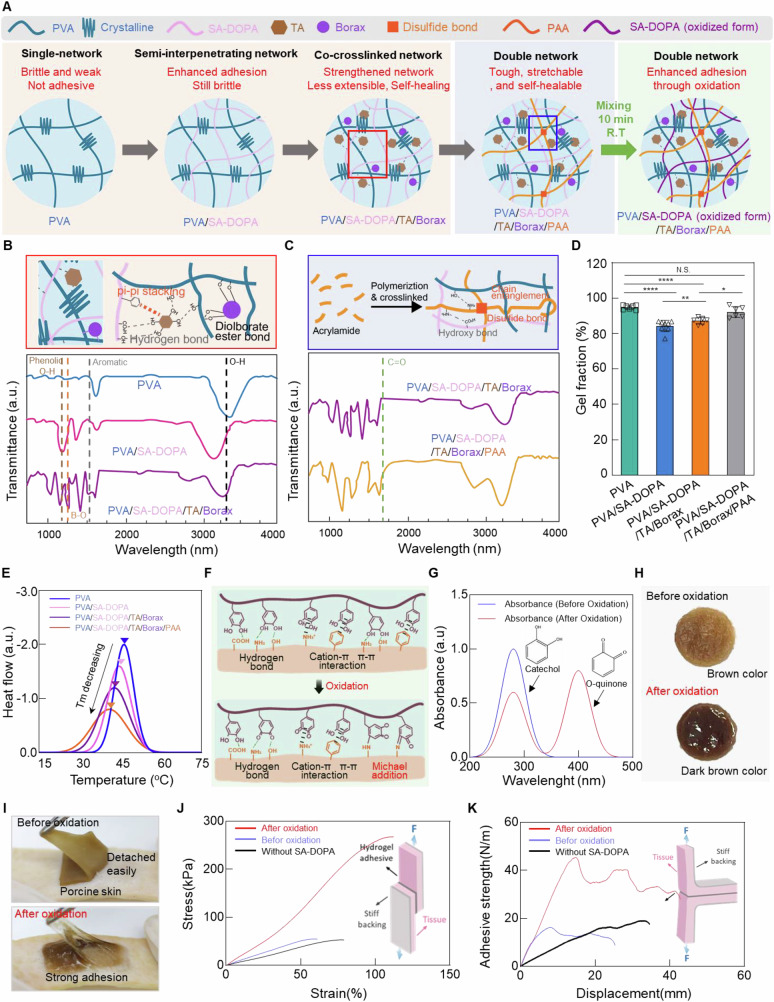
Fabrication and Structural Characterization of the STAT Hydrogel. **A** Schematic illustration of the four fabrication steps of the STAT hydrogel. Created using Adobe Illustrator. **B** Illustration and FT-IR spectra of the TA and borax network hydrogels. Created using Adobe Illustrator. **C** Illustration and FT-IR spectra of the double-network structure of PAA hydrogels. Created using Adobe Illustrator. **D** Gel fraction measurements for each hydrogel sample (*n* = 6: n is the sample size for each group). Data are presented as mean ± SEM. Statistical analysis was performed using a two-sided Student’s *t*-test. **E** DSC analysis of the hydrogel. **F** Schematic illustration of the process that converted catechol moieties into o-quinone species. Created using Adobe Illustrator. **G** UV–vis spectroscopy confirms the oxidative transformation of the hydrogel. **H** Photographs displaying the color change after oxidation of the hydrogel. **I** Photographs of pulling the hydrogel to detach from the porcine skin. **J** Shear strength of the hydrogel against porcine skin. Created using Adobe Illustrator. **K** 180-degree peel test of the hydrogel against porcine skin. Created using Adobe Illustrator. (*P < 0.05, **P < 0.01, ***P < 0.001*,* and ****P < 0.0001*). ns, not significant*. Exact *p*-values are reported in the Source Data.

Figure 2A schematically illustrates the four fabrication steps of the STAT hydrogel, involving the formation of a tough primary network and the addition of a ductile secondary network. The subsequent oxidation step promotes catechol-to-quinone conversion, generating multiple interactions, thereby enhancing interfacial adhesion. We used PVA as the backbone polymer. However, PVA hydrogels obtained through the freezing–thawing process are typically brittle and weak, exhibiting poor adhesion to tissue. To enhance tissue adhesion, SA-DOPA was incorporated into the PVA solution. The SA-DOPA concentration was systematically optimized, and 0.108 M was selected as the minimal level achieving saturated mechanical reinforcement (Supplementary Fig. 1). The flexible alginate chains physically entangle with the PVA network, and the catechol groups of DOPA form multiple hydrogen bonds with hydroxyl groups of PVA. This results in a semi-interpenetrating network with improved interfacial adhesion. The catechol groups of SA-DOPA are capable of not only extensive hydrogen bonding with neighboring molecules but also chemical interactions with amine (-NH₂) and carboxyl (-COOH) groups, thereby enhancing adhesion to biological substrates^63^. In the second step, TA and borax were introduced to reinforce the network. TA content was also optimized based on hemostatic performance, and 2000 mg was chosen since clotting enhancement saturated beyond this level (Supplementary Fig. 2). TA contains 25 phenolic hydroxyl groups per molecule. These groups are responsible for establishing multiple hydrogen bonds that crosslink the PVA–PVA, PVA–SA-DOPA, and SA-DOPA–SA-DOPA chains (Fig. 2B, red square box). This dense and reversible network of hydrogen bonds enables rapid reformation upon external damage, thereby imparting self-healing ability to the hydrogel. Additionally, borax forms diol-borate ester bonds with the hydroxyl groups of PVA, further stabilizing the network. These borate ester linkages are reversible under physiological conditions, contributing to repeated self-healing behavior^64^. In the third step, PAA was infused and polymerized within the primary network (PVA/SA-DOPA/TA/Borax) to generate the secondary network, completing the DN architecture (PVA/SA-DOPA/TA/Borax/PAA: STAT hydrogel network). Although multiple dynamic interactions are incorporated within the primary scaffold (e.g., TA-mediated hydrogen bonding and reversible borate ester linkages), only PAA forms an independently polymerized secondary interpenetrating network; therefore, the STAT system is best categorized as a reinforced double-network hydrogel rather than a multi-network hydrogel, consistent with the established definition of DN architectures^55,65^. The incorporation of the PAA network endowed the hydrogel with ductile and energy-dissipating properties, allowing large deformation without fracture, which improves overall mechanical toughness. This design synergistically combines a rigid, energy-dissipating network with a ductile, load-bearing network, providing both high strength and extensibility^66^. In the final step, adhesion was enhanced by gently kneading the STAT hydrogel to induce partial oxidation of catechol groups^67^. This oxidation of catechol to o-quinone is promoted under the mildly basic environment generated by borax and further assisted by dissolved oxygen during the kneading process. Notably, borax can reversibly coordinate with catechol moieties through borate-diol complexation, which helps suppress premature oxidation during static fabrication or storage^68,69^. Upon mechanical kneading, however, increased exposure to ambient oxygen and partial decomplexation of catechol sites trigger controlled catechol-to-quinone conversion, yielding sufficient interfacial reactivity for rapid wet-tissue bonding^70–72^. This mechanism was further supported by additional UV-vis analysis showing that borax-containing samples remained largely unoxidized under static conditions, whereas partial quinone formation was observed only when borax-containing systems were subjected to shearing/mixing (Supplementary Fig. 3). The resulting o-quinone then forms covalent bonds with amine or thiol groups on the tissue surface, which significantly increases adhesive strength. FT-IR spectra confirmed the sequential network formation of the STAT hydrogel. The formation of the primary network was monitored step-by-step, as shown in Fig. 2B. Initially, a broad O–H stretching band was observed at ~3300–3500 cm⁻¹, reflecting the hydroxyl groups of PVA. A new band corresponding to phenolic O–H bending (~1270 cm⁻¹) and additional aromatic signals (~1500–1600 cm⁻¹) were observed, confirming successful catechol grafting upon SA-DOPA incorporation. In the final step of primary network formation, the phenolic O–H peak intensified owing to the introduction of TA. Additionally, a distinct band at ~1330–1360 cm⁻¹ appeared, corresponding to the B–O stretching vibration of tetrahedral borate complexes. Subsequently, the formation of the secondary network was verified in Fig. 2C. After introducing the PAA network, a distinctive C = O stretching peak from PAA appeared at ∼1700 cm⁻¹, which was absent in the primary network. This confirms the successful construction of the DN architecture. Figure 2D shows the gel fraction analysis used to evaluate the network integrity of the hydrogels. The pristine PVA hydrogel displayed the highest gel fraction (~95%), which is attributed to its crosslinking primarily through semi-crystalline domains. In contrast, the semi-interpenetrating hydrogel (PVA/SA-DOPA) showed the lowest gel fraction, since the interpenetration of SA-DOPA disrupted the crystallinity of PVA and reduced the density of physical crosslinks. Subsequently, the gel fraction increased when TA and borax were introduced, reflecting the additional chemical crosslinks provided by phenolic interactions and borate ester formation. Finally, the STAT hydrogel demonstrated a further increase in gel fraction. This is attributed to the introduction of the secondary PAA network, which provided additional physical entanglements and multiple dynamic bonds. As a result, the gel fraction of the STAT hydrogel became statistically comparable to that of the pristine PVA hydrogel. This indicates that the dense, multi-component crosslinking within the dual-network structure effectively compensated for the initial loss of crystallinity caused by SA-DOPA incorporation. The gel fraction analysis provides a measure of the overall network stability. To understand how the other components specifically impacted the physical part of this network (the PVA crystalline domains), we performed differential scanning calorimetry (DSC). DSC analysis revealed a progressive decrease in the melting temperature (T_m_) from PVA to the STAT hydrogel. This indicates that the crystallinity of the PVA domains was increasingly disrupted by the incorporation of SA-DOPA, TA/borax, and PAA (Fig. 2E). This trend is consistent with the gel fraction analysis (Fig. 2D), where the initial reduction in crystallinity lowered the gel fraction of the semi-interpenetrating network. However, the subsequent introduction of chemical and dynamic crosslinks by TA/borax and PAA effectively counteracted this effect. These new interactions formed a robust, alternative network structure, compensating for this loss in physical crosslinking and restoring the gel fraction to a level comparable to that of pristine PVA. Together, these results suggest that the thermal transition temperature reflects decreased crystalline domains, while the gel fraction reflects reinforced crosslink density through multi-scale interactions. To enhance the adhesive performance, the hydrogel was gently kneaded in ambient air to promote partial oxidation of the catechol groups. As illustrated schematically, this process converted catechol moieties into o-quinone species (Fig. 2F). The generated oxidized catechol species can undergo covalent bonding through Michael-type addition reactions with nucleophilic groups (e.g., amines, thiols) present in biological tissues, thereby strengthening interfacial adhesion. This oxidative transformation was supported by UV–vis spectroscopy (Fig. 2G). Compared with the pre-oxidized state, the oxidized hydrogel exhibited a decrease in the catechol absorption peak (~280 nm), accompanied by the emergence of a new band around ~390 nm, consistent with the formation of quinone-like species. Consistent with these spectral changes, optical photographs also revealed a dark brown color after oxidation (Fig. 2H), suggesting catechol oxidation within the hydrogel network. To further support the contribution of quinone-mediated covalent bonding at the tissue interface, we performed a functional group blocking assay using sulfo-NHS acetate to mask primary amines on porcine skin. Amine blocking significantly reduced the adhesion of the oxidized STAT hydrogel after PBS rinsing (Supplementary Fig. 4), indicating that nucleophilic tissue amines participate in the oxidative catechol–quinone coupling mechanism. Adhesion testing on porcine skin demonstrated that pre-oxidized hydrogels adhered but could be removed easily, whereas oxidized hydrogels exhibited markedly stronger adhesion and resisted detachment (Fig. 2I and Supplementary Movie 1). In addition, because catechol–borate-assisted interactions remain partially reversible, adhesion could be gently reduced when required by applying a sorbitol-based triggering solution, enabling controlled detachment without disrupting the tissue surface (Supplementary Movie 2). Quantitative adhesion measurements using lap shear (Fig. 2J and Supplementary Fig. 5, 6A) and 180° peel tests (Fig. 2K and Supplementary Fig. 6B) revealed that oxidation increased adhesion strength by more than two-fold in both assays. This enhancement is attributed to covalent bonding through catechol–o-quinone conversion. To further demonstrate this strong adhesion in practical tissue conditions, Fig. S7A (Supplementary Information) presents photographs of the hydrogel attached to porcine skin under different deformations. The hydrogel remains firmly bound even during stretching, twisting, and bending, demonstrating its ability to adapt to the dynamic motions of biological tissues. Scanning electron microscopy (SEM) image reveals that the hydrogel seamlessly conforms to the irregular topography of the skin surface (Supplementary Fig. 7B). Additionally, the hydrogel shows the adhesion with various mouse organs, such as the heart, stomach, and kidneys (Supplementary Fig. 7C). These results confirm that the hydrogel achieves conformal and covalent adhesion to tissue, preventing mechanical mismatch. Such conformal contact allows the hydrogel to move in harmony with deforming tissues and ensures reliable wound sealing despite frequent patient motion. This robust tissue integration highlights the hydrogel’s potential as an effective wound dressing. To address concerns regarding the controllability of catechol oxidation, we performed additional experiments under defined mechanical mixing conditions. As shown in Supplementary Fig. 8A, STAT hydrogels were prepared using a standardized mechanical mixing condition. Representative photographs collected at different mixing times (Supplementary Fig. 8B) show a gradual darkening of the hydrogel, indicating progressive catechol oxidation during mixing. This visual change was quantitatively analyzed by ImageJ (Supplementary Fig. 8C), confirming a time-dependent increase in color intensity. Importantly, UV–vis spectroscopy further verified the underlying chemical conversion (Supplementary Fig. 8D): the characteristic catechol absorption band at ~280 nm decreased, while a new oxidation-associated band emerged around ~390–400 nm, consistent with catechol-to-o-quinone transformation. These results confirm that catechol oxidation proceeds in a controlled, mixing-time-dependent manner rather than occurring stochastically. Next, we examined how this programmable oxidation translates into mechanical and adhesive performance. Using a rheometer-based measurement, the adhesion force of STAT hydrogels was quantified as a function of mixing time (Supplementary Fig. 9A). Adhesion increased progressively with mixing duration and reached an optimum at 15 min, which was therefore selected as the standard fabrication condition in this study. Importantly, the mixing-dependent oxidation translated into tunable mechanical and adhesive performance. Adhesion strength and mechanical robustness increased progressively with mixing time, reaching an optimum at 15 min; however, further mixing beyond this point resulted in a pronounced decline in both adhesion and mechanical properties (Supplementary Fig. 9B–D). We attribute this decrease to excessive water loss during prolonged mixing. Consistently, gravimetric analysis revealed substantially greater hydrogel mass reduction at 20 min compared with 15 min, confirming accelerated dehydration under extended mixing conditions (Supplementary Fig. 9E). Finally, STAT hydrogels mixed for 15 min exhibited further mechanical stabilization during room-temperature aging, as evidenced by time-dependent stress-strain behavior and quantified mechanical properties (Supplementary Fig. 9F, G). Collectively, these data demonstrate that STAT oxidation and its resulting adhesive and mechanical performance are highly controllable and reproducible through defined mixing time, directly addressing concerns regarding process variability. In addition, to further confirm that catechol oxidation can be controlled not only during fabrication but also during storage, we examined the effect of limiting oxygen exposure. STAT hydrogels were stored in sealed containers under an N₂-purged atmosphere (Supplementary Fig. 10A). After one week, hydrogels stored under ambient conditions exhibited pronounced color darkening, whereas N₂-protected samples showed markedly reduced discoloration, indicating suppressed oxidative progression (Supplementary Fig. 10B). Quantitative image analysis confirmed significantly attenuated changes in normalized brightness under oxygen-limited storage (Supplementary Fig. 10C). Importantly, STAT hydrogels preserved under N₂ maintained their mechanical and adhesive performance after one week, demonstrating that oxidation-induced property changes can be effectively minimized through controlled storage conditions (Supplementary Fig. 10D).

### Mechanical and functional assessment of the STAT hydrogel

**Fig. 3:**
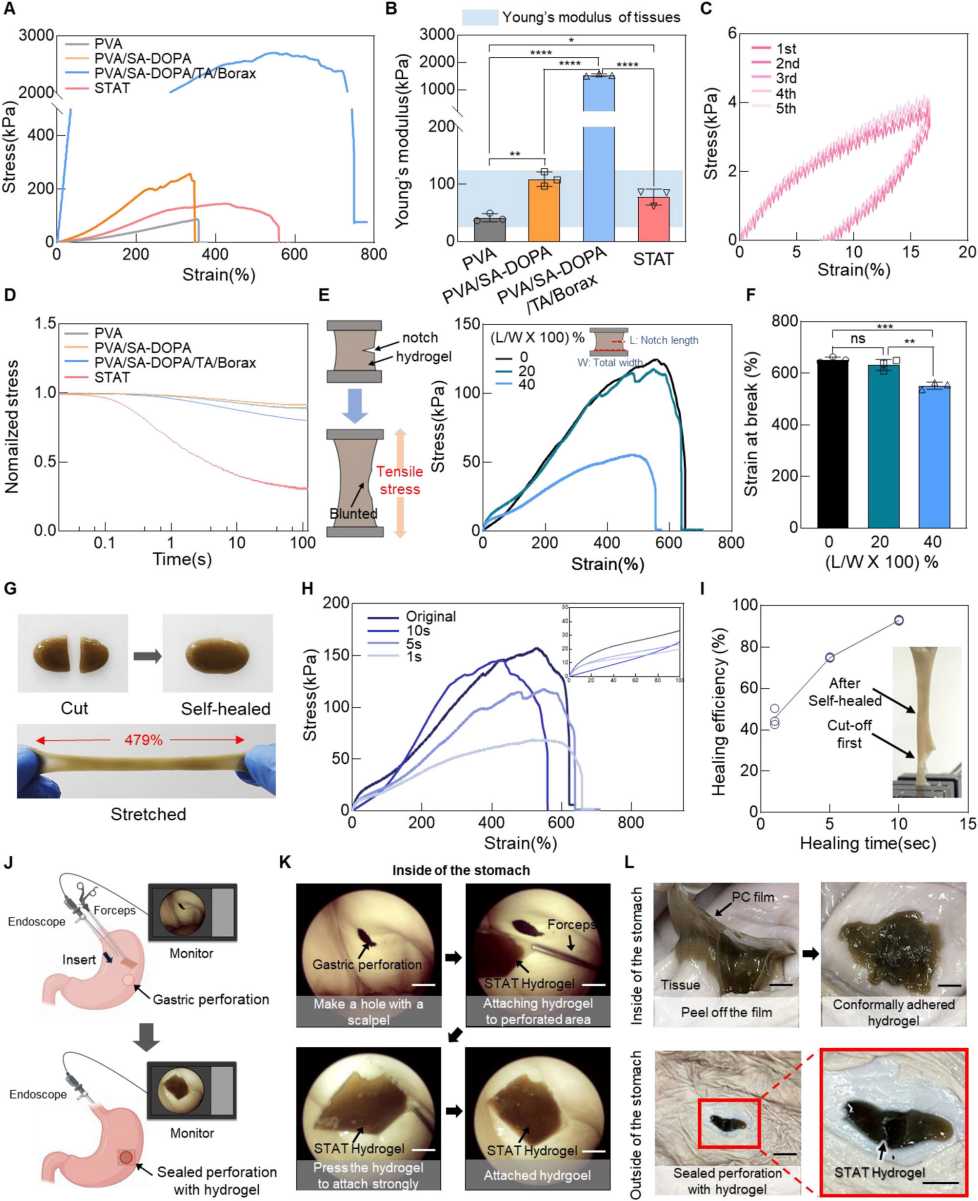
Mechanical and functional assessment of the STAT hydrogel. **A** Tensile test measurement of hydrogel in each fabrication step. **B** Young’s moduli of hydrogel in each fabrication step (*n* = 3: n is the sample size for each group). Data are presented as mean ± SEM. Statistical analysis was performed using one-way ANOVA followed by Tukey’s post hoc test. **C** Cyclic loading-unloading tests for the STAT hydrogel. **D** Stress-relaxation test for hydrogel in each fabrication step. **E** Schematic illustration describing the notch-insensitive property and strain at break values of the STAT hydrogel with varying notch lengths. Created using Adobe Illustrator. **F** Statistical analysis of the strain-at-break of the notch-introduced STAT hydrogel (*n* = 3: *n* is the sample size for each group). Data are presented as mean ± SEM. Statistical analysis was performed using one-way ANOVA followed by Tukey’s post hoc test. **G** Optical photographs showing the self-healing behavior of the STAT hydrogel. **H** Tensile test curves for the self-healed hydrogels with varying healing times. **I** Self-healing efficiency of the STAT hydrogel as a function of healing time. (*n* = 3: *n* is the sample size for each time point). **J** Illustration of the STAT hydrogel-mediated gastric perforation sealing experiment. Created using Adobe Illustrator. **K** Experimental procedure for sealing a gastric perforation using the STAT hydrogel. **L** Post-experiment photographs of the stomach’s inside and outside surfaces (*P < 0.05, **P < 0.01, ***P < 0.001, and ****P < 0.0001*). ns, not significant*. Exact *p*-values are reported in the Source Data.

Hydrogels possess a tissue-like softness; however, they generally suffer from low mechanical strength and inherent brittleness^73^. Such mechanical fragility should be overcome for their practical applications. In particular, tissue environments where hydrogels are applied undergo continuous deformation and external loads^74^. Easy crack propagation within the hydrogel structure inevitably compromises long-term stability. Our stepwise crosslinked the STAT hydrogel achieves high toughness and fracture resistance through energy dissipation mechanisms embedded within the network. This structure exhibits notch-insensitive behavior, meaning that even in the presence of minor defects or damage, catastrophic failure is avoided. Furthermore, reversible hydrogen bonding from TA and borate ester linkages formed by borax enables rapid network reconfiguration, thereby imparting self-healing functionality. To compare mechanical behavior, tensile stress–strain curves were measured for single-network PVA, semi-interpenetrating network PVA/SA-DOPA, co-crosslinked network PVA/SA-DOPA/TA/Borax, and the STAT hydrogel (PVA/SA-DOPA/TA/Borax/PAA) (Fig. 3A). The PVA single network exhibited a tensile strength above ~100 kPa with an elongation at break limited to ~370%. Incorporation of SA-DOPA increased the tensile strength to over ~250 kPa, owing to additional hydrogen bonding and catechol-mediated interactions within the polymer network. The addition of TA and borax further enhanced both strength and extensibility, reaching stresses of almost 2800 kPa and strains up to ~750%. In contrast, the final STAT hydrogel showed moderate strength, almost 150 kPa, with high extensibility ~580%, indicating that the stepwise introduction of dissipative components yields a well-balanced network with improved toughness. Young’s modulus measurements (Fig. 3B) demonstrated a progressive increase in stiffness with the incorporation of additional crosslinking components. The single-network PVA exhibited the lowest modulus (~30 kPa), while the introduction of SA-DOPA raised the modulus to ~120 kPa, which is higher than that of typical soft tissues. Co-crosslinking with TA and borax further reinforced the network, yielding the highest modulus (~1200 kPa). In contrast, the STAT hydrogel displayed a reduced modulus (~80 kPa), which falls within the typical range of soft tissues (e.g., liver, myocardium, skeletal muscle, skin), thereby indicating favorable mechanical compatibility with biological environments. This reduction arises from the introduction of a soft, energy-dissipating second network (PAA), which lowers the initial stiffness while enhancing overall toughness and compliance^61^. To evaluate energy dissipation under cyclic deformation, five consecutive loading–unloading cycles were performed (Fig. 3C). The STAT hydrogel exhibited a large hysteresis loop in the first cycle and maintained a similar curve shape in subsequent cycles, demonstrating repeated energy dissipation capability. This behavior arises from the continuous reformation of TA-based reversible bonds under external stress, which stabilizes the network. These findings indicate that the stepwise crosslinking strategy effectively enhances the hydrogel’s mechanical strength, ductility, and ability to sustain toughness over repeated loading. Stress relaxation behavior was further compared by measuring normalized stress–time curves (Fig. 3D). The stress of each sample was monitored over time under a constant strain of 15%. The PVA single network and SA-DOPA-modified network maintained higher initial stress retention but exhibited gradual decay over time due to insufficient dissipative components. Networks containing TA and borax showed progressive stress relaxation under sustained load due to partial dissociation of hydrogen and borate ester bonds. The STAT hydrogel demonstrated rapid stress relaxation, with stress levels decreasing by half within a few seconds, indicating active network rearrangement via reversible bonding. Notch-insensitive behavior was assessed through tensile tests on samples with varying notch lengths (Fig. 3E, F, and Supplementary Fig. 11). Whereas conventional hydrogels fracture readily when notched, our hydrogel maintained elongation beyond 600% even when notched to 20% of its width. This is attributed to the sacrificial bonds from TA and borax that effectively dissipate stress, while PAA chains preserve structural continuity and suppress crack propagation. During tensile deformation, crack blunting rather than sharp propagation was observed. To further validate that this notch tolerance arises from the reinforced dynamic DN architecture, we additionally performed the same notch tensile test using a pristine single-network PVA hydrogel as a control (Supplementary Fig. 12A–C). In contrast to STAT, the PVA hydrogel exhibited pronounced notch sensitivity, with rapid crack initiation and premature fracture upon the introduction of a notch. This comparison highlights that the brittle nature of physically crosslinked PVA alone is insufficient to delocalize stress concentration at the crack tip, whereas the incorporation of dynamic sacrificial interactions (TA/borax) together with the ductile PAA secondary network enables efficient energy dissipation and crack-arresting behavior. Self-healing performance was evaluated by rejoining cut hydrogel specimens and monitoring the recovery of mechanical properties over time. As shown in Fig. 3G, cut samples adhered within seconds and could be stretched up to 479% after healing. Analysis of tensile–unloading curves (Fig. 3H) revealed partial recovery of mechanical strength within 1 s of contact, with nearly complete restoration of the original stress–strain profile within 10 s. This rapid repair is due to the simultaneous reformation of TA-mediated multiple hydrogen bonds and reversible covalent bonds in the PAA backbone^55^. Quantitative self-healing efficiency analysis (Fig. 3I and Supplementary Fig. 5) demonstrated that over 50% of the original strength was recovered after 1 s, and more than 90% after 10 s. This healing rate is markedly faster than that of most self-healing hydrogels (which require hours to tens of hours) and maintains high viscoelastic stability. Taken together, the STAT hydrogel’s combination of notch insensitivity and ultra-rapid self-healing enables it to maintain long-term structural stability in mechanically dynamic and damage-prone biological environments. Fig. 3J and Movie S3 (Supplementary Information) present a schematic of the hydrogel delivery process using an endoscope. The hydrogel was first affixed to a polycarbonate (PC) film, grasped with forceps, and guided toward an artificially created gastric perforation in an ex vivo porcine stomach model. Under real-time endoscopic visualization, the hydrogel was delivered and attached to the perforated site. Figure 3K sequentially depicts the procedure as observed through the endoscopic camera. Following perforation creation using a scalpel, the hydrogel was positioned at the defect site via forceps and firmly pressed onto the tissue surface. The hydrogel adhered stably even within the highly hydrated gastric environment, indicating excellent delivery and adhesion performance under wet conditions. Figure 3L shows both internal and external tissue views after hydrogel attachment. Internally, after removal of the PC film, the hydrogel remained tightly adhered to the tissue surface. External observation revealed that the perforation was completely sealed by the hydrogel. Magnified images showed full coverage of the defect with seamless integration into surrounding tissue, clearly demonstrating the hydrogel’s strong adhesion and sealing capability in high-moisture environments.

### Antibacterial activity, hemostatic effects, and biocompatibility of the STAT hydrogel

**Fig. 4:**
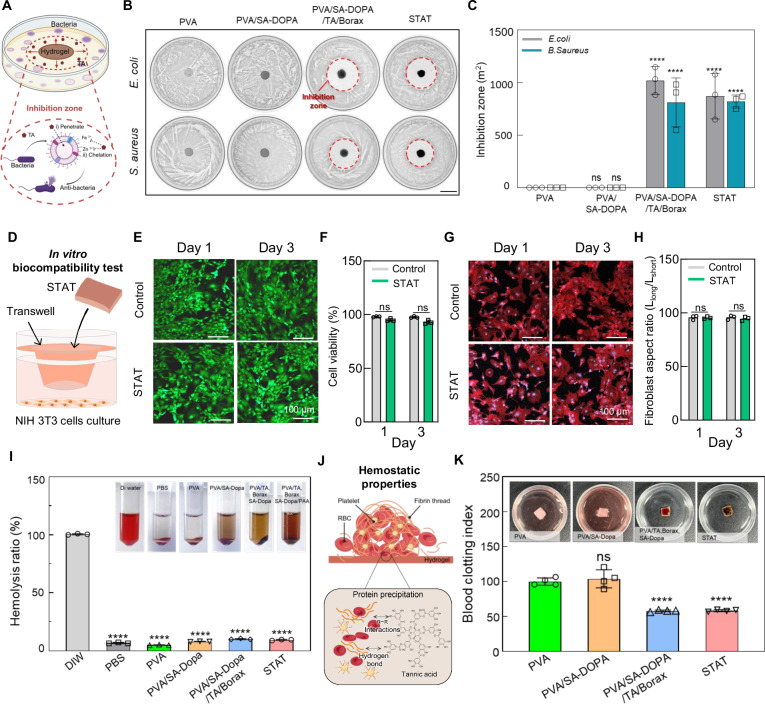
Antibacterial activity, hemostatic effects, and biocompatibility of the STAT hydrogel. **A** Schematic illustration of the antibacterial performances of the hydrogel. Created in BioRender. Park, K. (2026). https://BioRender.com/y3jedza. **B** The growth of E. coli and S. aureus bacterial colonies (scale bars: 20 mm). The inhibition zones are marked with red dashed lines. **C** Quantitative analysis of the inhibition zone diameters surrounding the hydrogels after a 12 h incubation period (*n* = 3: *n* is the sample size for each group). Data are presented as mean ± SEM. Statistical analysis was performed using one-way ANOVA followed by Tukey’s post hoc test. **D** Schematic illustration describing the in vitro biocompatibility test. Created using Adobe Illustrator. **E** Live/Dead staining of NIH3T3 cells (green: live cells, red: dead cells) for in vitro biocompatibility testing (scale bars: 100 µm). **F** Cell viability calculated from the live/dead assay (*n* = 3: *n* is the sample size for each group). Data are presented as mean ± SEM. Statistical analysis was performed using a two-sided Student’s t-test. **G** Fluorescence images of actin filaments using phalloidin staining. Cell nuclei were co-stained using DAPI (scale bars: 100 µm). **H** Quantification of cell aspect ratios is defined as the ratio of the major to the minor cell axis (*n* = 3: *n* is the sample size for each group). Data are presented as mean ± SEM. Statistical analysis was performed using a two-sided Student’s *t*-test. **I** Optical image of hemolysis test on hydrogels and hemolysis ratio of each group. (*n* = 3: *n* is the sample size for each group). Data are presented as mean ± SEM. Statistical analysis was performed using one-way ANOVA followed by Tukey’s post hoc test. **J** Schematic illustration describing the hemostatic effects of the hydrogel. Created in BioRender. Park, K. (2026). https://BioRender.com/y3jedza. **K** Photographs of blood clotting on each hydrogel and blood clotting index value for each group (*n* = 4: *n* is the sample size for each group). Data are presented as mean ± SEM. Statistical analysis was performed using one-way ANOVA followed by Tukey’s post hoc test. (*P < 0.05, **P < 0.01, ***P < 0.001, and ****P < 0.0001*). ns, not significant*. Exact *p*-values are reported in the Source Data.

Figure 4A schematically illustrates the antibacterial mechanism of the hydrogel. For wound-healing materials, the ability to effectively suppress bacterial infection at the wound site is essential, as bacterial colonization can delay tissue regeneration and lead to severe complications^75^. The multiple phenolic hydroxyl groups in TA can bind to metal ions or induce protein denaturation, thereby disrupting bacterial cell membranes and inhibiting metabolic activity^76^. As a result, incorporation of TA into the hydrogel network provides sustained antibacterial activity at the material’s surface. This effect was clearly evidenced by optical images (Fig. 4B), which showed that only TA-containing hydrogels produced a distinct inhibition zone against both Gram-negative (G⁻) and Gram-positive (G⁺) bacteria. Furthermore, quantitative analysis (Fig. 4C) confirmed these findings: groups lacking TA exhibited negligible antibacterial activity, whereas TA-containing hydrogels showed pronounced antibacterial effects. The cell biocompatibility of the prepared hydrogels was then evaluated. For biomaterials intended for clinical application, the absence of cytotoxicity is a fundamental requirement. Live/Dead staining (Fig. 4E) revealed no significant decrease in cell viability in STAT hydrogel-treated groups compared with the control. Quantitative assessment via CCK-8 assay (Fig. 4F) similarly showed that all STAT hydrogel groups maintained cell viability comparable to the control, indicating minimal cytotoxicity. Furthermore, phalloidin staining revealed no morphological changes in cells relative to the control group (Fig. 4G, H). This confirms that the STAT hydrogels did not induce cytoskeletal disruption or abnormal cell spreading. The hemolytic potential of the hydrogels was also assessed (Fig. 4I). Since these materials may come into direct contact with blood in biomedical applications, it is essential that they exhibit hemocompatibility and do not induce hemolysis of red blood cells (RBCs). As expected, RBCs suspended in deionized (DI) water underwent complete hemolysis due to osmotic imbalance, producing a strong red color. In contrast, both phosphate-buffered saline (PBS) and all hydrogel groups exhibited comparably low hemolysis rates because their isotonic environments prevented hemolysis. This finding indicates that the hydrogels are non-hemolytic and safe for contact with blood. Figure 4J demonstrates the rapid blood coagulation-inducing ability of TA-containing hydrogels. The phenolic hydroxyl groups in TA strongly interact with plasma proteins and platelets, promoting protein denaturation and platelet activation. This accelerates platelet aggregation and fibrin network formation, resulting in rapid coagulation upon contact between blood and the hydrogel surface. Hydrogels without TA allowed blood to spread widely across the surface with little coagulation, whereas TA-containing hydrogels produced visible coagulation clots within a short time (inset images). Finally, blood clotting performance was quantitatively evaluated. Blood collected from mice was dropped onto each hydrogel surface and, after 10 s, immersed in PBS. Only the TA-containing hydrogels showed clear clot formation. The blood clotting index (BCI) was calculated to evaluate hemostatic performance (Fig. 4K). Both the PVA/SA-DOPA/TA/Borax and STAT hydrogels exhibited low BCI values of 56.40 ± 1.5 and 58.40 ± 1.7, respectively. These values were approximately two-fold lower than those of the TA-free controls (PVA and PVA/SA-DOPA), a significant improvement attributed to the procoagulant activity of TA. In addition, an in vitro platelet-rich plasma (PRP) interaction assay further showed that STAT significantly promoted platelet association with the hydrogel surface compared with the PVA control (Supplementary Fig. 12D), supporting platelet-involved clot stabilization as a contributing mechanism to the rapid hemostatic performance. Notably, the incorporation of the secondary PAA network did not compromise the coagulation performance, as the TA-containing hydrogels exhibited similarly low BCI values regardless of PAA addition. This confirms that hemostatic activity is primarily governed by TA-mediated interactions, while PAA mainly serves as a mechanical reinforcement network. Collectively, these results demonstrate that the hydrogels possess a unique combination of properties: TA-mediated surface antibacterial activity, excellent cell compatibility, low hemolytic potential, and superior hemostatic capability. This multifaceted functionality makes them promising candidates for wound-healing patches in clinical applications.

### In vivo screening and preclinical evaluation of hemostatic performance

**Fig. 5:**
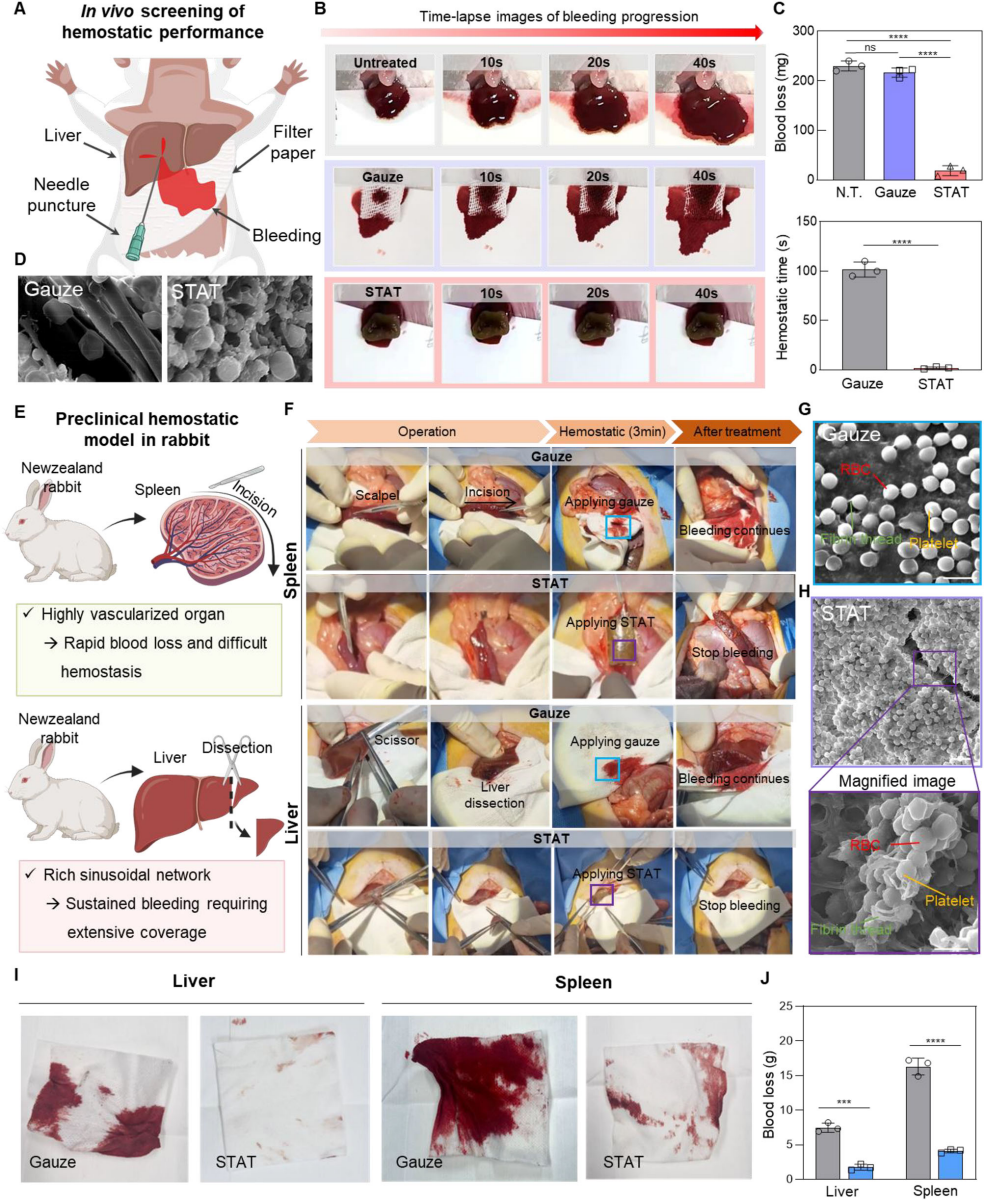
In Vivo Screening and Preclinical Evaluation of Hemostatic Performance. **A** Schematic illustration describing a hemostatic experiment in a mouse model. Created using Adobe Illustrator. **B** Optical photographs of the untreated, gauze, STAT hydrogel groups in the mouse liver hemorrhage model (*n* = 3: *n* is the sample size for each group). Created in BioRender. Park, K. (2026). https://BioRender.com/y3jedza. **C** Accumulated blood loss and hemostatic time comparisons among different hemostat treatments (*n* = 3: *n* is the sample size for each group). Data are presented as mean ± SEM. Statistical analysis was performed using one-way ANOVA followed by Tukey’s post hoc test. **D** SEM image of gauze and STAT hydrogel after blood exposure. **E** Schematic illustration of the preclinical hemostatic model in a rabbit. Created in BioRender. Park, K. (2026). https://BioRender.com/y3jedza. **F** Optical photographs of the gauze and the STAT hydrogel groups in rabbit spleen and liver hemorrhage models (*n* = 3: *n* is the sample size for each group). **G** SEM image of gauze group surface after hemostasis experiment (scale bars: 5 µm). **H** SEM image of the STAT hydrogel group surface after hemostasis experiment (scale bars: 100 µm, 5 µm, respectively) (*n* = 5: *n* is the sample size for each group). **I** Photographs showing blood loss after hemostasis. **J** Accumulated blood loss comparisons among liver and spleen treatments (*n* = 3: *n* is the sample size for each group). Data are presented as mean ± SEM. Statistical analysis was performed using a two-sided Student’s *t*-test. (*P < 0.05, **P < 0.01, ***P < 0.001, and ****P < 0.0001)*. ns, not significant*. Exact *p*-values are reported in the Source Data.

To assess the in vivo efficacy and suitability of the hydrogel as a wound dressing, we prepared it in patch form and evaluated its performance using a murine model. Initially, the biocompatibility of the STAT hydrogel patch was examined through histological analyses and hepatotoxicity assessments. The patches were subcutaneously implanted into mice, which were sacrificed seven days post-surgery. A sham group that underwent incision without patch implantation was also included for comparison. The implantation sites were visually monitored immediately after surgery and on day 7 (Supplementary Fig. 6). Photographs revealed no visible signs of acute local inflammation (e.g., swelling, erythema, blistering, infiltration, or ulceration) in the areas where the patches were implanted. To further investigate tissue responses, histological staining (Hematoxylin and Eosin (H&E) and Masson’s trichrome (MT)) and immunohistochemical Toluidine blue (TB) staining were conducted on tissues adjacent to the implant (Supplementary Fig. 7B). The H&E and MT results indicated normal tissue architecture with no observable cytotoxicity, while TB staining revealed no significant increase in inflammatory cell infiltration compared to the sham group. Additionally, hepatotoxicity was evaluated by measuring serum alanine aminotransferase (ALT) and aspartate aminotransferase (AST) levels before implantation and seven days afterward in normal, sham, and patch-implanted groups. As shown in Fig. S7C, there were no statistically significant differences in ALT or AST levels among the groups, suggesting that the hydrogel patch does not induce systemic liver toxicity. Collectively, these results confirm that the STAT hydrogel patch exhibits favorable biocompatibility and is suitable for use as a wound dressing material in vivo. Following verification of its biocompatibility, in vivo experiments were conducted using both small (mouse) and large (rabbit) animal models to assess the hemostatic performance of the hydrogel. For screening of hemostatic performance, a mouse liver puncture model was used to assess whether the hydrogel could achieve effective hemostasis under in vivo conditions (Fig. 5A). In the untreated group, bleeding continued without signs of clot formation throughout the observation period, resulting in progressive blood loss. In the gauze-treated group, partial absorption of blood occurred; however, noticeable bleeding persisted even after 40 seconds. In contrast, the STAT hydrogel-treated wounds rapidly formed a stable clot, with coagulation initiated within a few seconds and near-complete hemostasis (Fig. 5B). Quantitative analysis further supported these observations (Fig. 5C). The STAT hydrogel group exhibited a dramatic reduction in total blood loss compared to both the untreated and gauze groups. Furthermore, the hemostatic time was shortened to just a few seconds. These results highlight the superior hemostatic efficacy of the hydrogel. In addition, histological analysis of the puncture site confirmed initial clot sealing at day 0 and substantial reduction of the defect space with progressive repair tissue formation by day 7 (Supplementary Fig. 16B), suggesting that STAT not only achieves rapid hemostasis but also supports subsequent wound closure in liver tissue. To investigate the hemostatic mechanism, the surfaces of gauze and STAT hydrogel were examined via SEM (Fig. 5D). The gauze surface exhibited only limited adhesion of RBCs and platelets, whereas the STAT hydrogel surface displayed dense aggregates of RBCs and platelets embedded within a compact fibrin network. After seven days, the STAT hydrogel remained in place without detachment, indicating stable adhesion at the implantation site. In contrast, the gauze group exhibited undesirable outcomes. Although the gauze remained positioned over the liver, it became entangled with surrounding tissues due to scab formation and inter-tissue interactions. This entanglement ultimately led to undesirable adhesion between the gauze and adjacent organs (Supplementary Fig. 8). This observation suggests that the TA in the STAT hydrogel promotes rapid aggregation of blood components and formation of a hemostatic network. Next, the hemostatic efficacy was assessed in a preclinical large-volume bleeding model using rabbit spleen and liver injury models. For rigorous benchmarking against clinically established hemostatic materials, we additionally performed comparative hemostasis experiments using two FDA-approved standards: fibrin glue as a representative liquid-type sealant and TachoSil as a patch-type hemostatic dressing (Fig. 5E and Supplementary Fig 4, Movies 4, 5). The spleen, which receives approximately 20–25% of the cardiac output and has one of the highest blood flow rates per tissue mass, undergoes massive hemorrhage upon injury. Such bleeding is difficult to control due to its dense microvascular network, high perfusion pressure, and soft parenchymal tissue that resists compression. The liver dissection model mimics extensive organ damage from trauma or surgery, resulting in continuous bleeding over a large area and requiring broad coverage. In both the spleen and liver injury models, applying gauze, fibrin glue, and TachoSil patch failed to achieve reliable hemostasis: even after 3 min of compression, active bleeding persisted from the cut surface (Fig. 5F). In contrast, the STAT hydrogel formed an immediate conformal seal on the wet tissue upon application, rapidly facilitating clot formation at the interface and stopping bleeding within seconds. In the spleen model, incision with a scalpel resulted in persistent surface bleeding that could not be controlled by gauze but was immediately sealed by the STAT hydrogel. Similarly, in the liver dissection model, bleeding continued despite gauze compression. In contrast, the STAT hydrogel adhered firmly to the irregular and large wound site, maintaining a stable hemostatic state throughout the observation period. SEM analysis of the interaction between the bleeding site and the hemostatic material revealed that gauze surfaces retained only a small number of platelets and RBCs (Fig. 5G), whereas the STAT hydrogel surfaces displayed abundant platelets and RBCs tightly bound within a dense fibrin mesh (Fig. 5H). This confirms that the STAT hydrogel induces rapid TA-mediated coagulation and thrombus network formation. Additionally, residual blood volume was compared among bleeding sites treated with gauze, fibrin glue, a TachoSil patch, or the STAT hydrogel (Fig. 5I and Supplementary Fig. 17A). The gauze, fibrin glue, and TachoSil patch -treated group retained substantial amounts of blood, whereas the STAT hydrogel-treated group left only negligible traces. Quantitative analysis demonstrated that residual blood volume in the STAT hydrogel group was markedly reduced compared to the other groups (Fig. 5J and Supplementary Fig. 17B). Overall, the STAT hydrogel integrates strong tissue adhesion with TA-driven rapid coagulation, enabling effective hemostasis even in high-flow and large-area bleeding conditions. These findings suggest that the STAT hydrogel has strong potential as an emergency hemostatic patch for a variety of clinical scenarios.

### Injectable STAT hydrogel for wound closure and healing

**Fig. 6:**
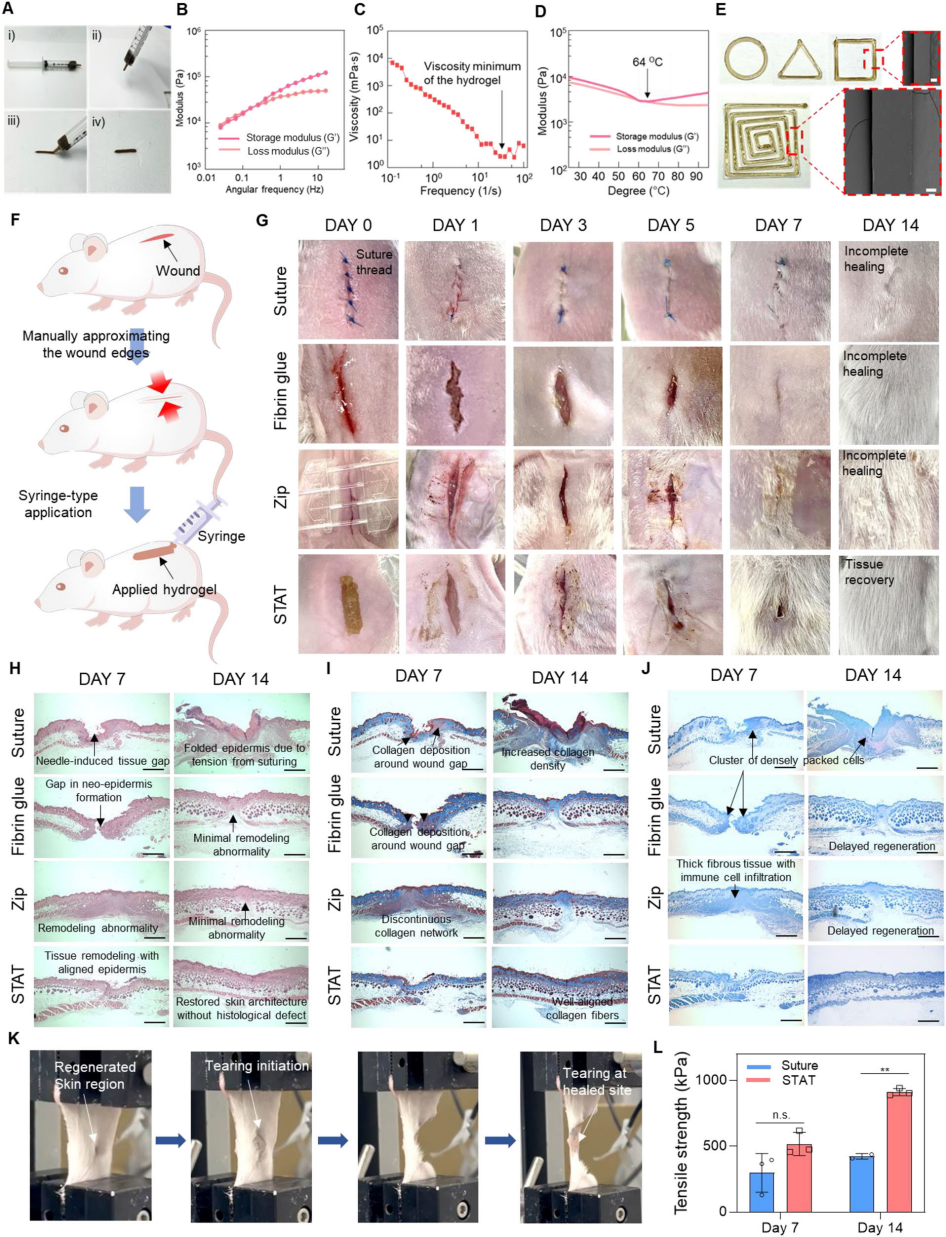
Injectable STAT hydrogel for wound closure and healing. **A** Sequential optical image of the use of a syringe-type hydrogel. **B** Storage modulus (G′) and loss modulus (G″) as a function of angular frequency. **C** Viscosity as a function of shear rate. **D** Storage modulus (G′) and loss modulus (G″) as a function of temperature. **E** 2D-printing performance of various shapes with the STAT hydrogel through 400 μm diameter nozzles, and SEM images. (scale bar: 100 µm) (*n* = 3: *n* is the sample size for each group). **F** Schematic illustration of skin incision wounds sealing by the STAT hydrogel. Created using Adobe Illustrator. **G** Photographs of the wound area after treatment with suture, fibrin glue, Zip band and STAT hydrogel, respectively (scale bar:1 cm). **H**–**J** H&E, MT and TB staining images of the repaired skin after 7 and 14 days (scale bar: 100 µm) (*n* = 4: *n* is the sample size for each group). **K** Tensile test images of the repaired skins (suture) after 14 days (**L**) The ultimate tensile strength of the healed skin in the suture and the STAT hydrogel groups on 7 and 14 days post-operation, respectively (*n* = 3: n is the sample size for each group). Data are presented as mean ± SEM. Statistical analysis was performed using a two-sided Student’s t-test. (*P < 0.05, **P < 0.01, ***P < 0.001*,* and ****P < 0.0001*). ns, not significant*. Exact *p*-values are reported in the Source Data.

To leverage the shear-thinning properties of the STAT hydrogel for wound closure and healing, its printability and syringe-based injectability were first evaluated. As shown in sequential optical images (Fig. 6A), the STAT hydrogel could be smoothly extruded through a standard medical syringe without clogging (ii), forming a continuous filament upon deposition (iii) and maintaining its shape after release (iv). These demonstrations were performed using syringe extrusion to evaluate the injectability and continuous filament formation of the hydrogel under clinically relevant conditions. These results demonstrate that the STAT hydrogel possesses excellent injectability and shape fidelity, supporting the feasibility of syringe-type application for on-demand wound sealing and minimally invasive delivery. The STAT hydrogel was also characterized at room temperature (25 °C) to evaluate its viscoelastic stability under clinically relevant conditions. Frequency sweep measurements revealed that the STAT hydrogel maintained a stable elastic-dominant behavior storage modulus (G′) consistently higher than loss modulus (G″) across the tested range (Fig. 6B), demonstrating sufficient mechanical integrity to support syringe-based extrusion and stable performance. To further substantiate these observations, rheological analysis was carried out to quantitatively characterize the shear-thinning behavior of the STAT hydrogel. Under frequency sweep conditions, the viscosity decreased with increasing frequency, reaching a minimum at approximately 100 Hz, indicating the characteristic relaxation frequency of the STAT hydrogel network (Fig. 6C). This point served as a key indicator for establishing the minimum shear stress required for extrusion through a nozzle, and was subsequently used to optimize the printing conditions. By fixing the frequency and varying the temperature, the G′ and G″ were measured (Fig. 6D), and the crossover point was identified. Because this crossover represents the sol–gel transition temperature where the material shifts from a liquid-like to a solid-like state, it was selected as the optimal printing temperature. Accordingly, the printing process was conducted at 64 °C under atmospheric pressure. Under these optimized conditions, the STAT hydrogel could be stably printed into various geometries (e.g., circle, triangle, square, spiral), all of which maintained structural integrity without collapse, demonstrating a high resolution of 400 µm. This experiment was performed to demonstrate the structural printability and shape fidelity of the hydrogel using a low-resolution 3D printing process. High-magnification SEM images further confirmed that the printed constructs exhibited a uniform and smooth surface morphology (Fig. 6E and Supplementary Fig. 9). The wound closure capability of the STAT hydrogel was then assessed in a mouse dorsal skin incision model (Fig. 6F). After approximating the wound edges, the hydrogel was injected via syringe to serve as the STAT hydrogel-based sealant. For in vivo wound treatment, the hydrogel was applied via syringe extrusion rather than 3D printing, as precise architectural patterning is not required for wound coverage, and syringe delivery better reflects clinically relevant application conditions. Unlike conventional sealants that primarily provide mechanical closure, the STAT hydrogel simultaneously adhered to the tissue and retained a moist microenvironment at the wound site. Such moisture retention is widely recognized to accelerate re-epithelialization, reduce scab formation, and support cell migration, thereby creating favorable conditions for effective wound regeneration. Four groups were compared: suture, fibrin glue, Zip-type wound closure band, and the STAT hydrogel. Observation of the healing process over 14 days (Fig. 6G) showed that all groups exhibited partial wound opening up to day 7. However, the STAT hydrogel group maintained nearly complete closure by day 7 and demonstrated complete recovery by day 14. To evaluate whether this macroscopic recovery was accompanied by proper tissue-level regeneration, histological analysis was carried out (Fig. 6H–J). H&E staining revealed distinct differences among treatment groups (Fig. 6H). The suture group exhibited needle-induced tissue gaps and epidermal folding caused by mechanical tension. In contrast, the fibrin glue group showed insufficient mechanical integrity by day 7, resulting in unsealed wound edges and persistent tissue gaps. The Zip band-treated wounds achieved relatively rapid closure by day 7, but abnormal epidermal architecture and cell infiltration were apparent. In contrast, the STAT hydrogel group displayed aligned epidermal layers and complete restoration of skin architecture by day 14, without detectable histological defects. Masson’s trichrome staining further highlighted the superiority of the STAT hydrogel treatment (Fig. 6I). The suture and fibrin glue groups showed discontinuous collagen deposition, while the Zip band group exhibited irregular collagen alignment. In contrast, the STAT hydrogel group demonstrated dense, continuous, and well-organized collagen fibers, indicative of effective matrix remodeling. Toluidine blue staining was used to assess inflammatory cell responses (Fig. 6J). Excessive infiltration of inflammatory cells was observed in the suture and Zip band groups, accompanied by delayed regeneration. In contrast, the STAT hydrogel-treated wounds showed minimal inflammatory cell presence, suggesting that the material suppresses unnecessary immune reactions and establishes a stable microenvironment conducive to tissue regeneration. The mechanical strength of the regenerated skin was evaluated via tensile testing (Fig. 6K). When the healed skin was subjected to tensile loading, the STAT hydrogel-treated group exhibited markedly improved resistance to tearing compared with the suture group. Quantitative analysis further confirmed that the breaking strength of the STAT hydrogel-treated skin was significantly higher than that of sutured skin, demonstrating that the STAT hydrogel application led to more effective mechanical regeneration of the wound tissue (Fig. 6L). For reference, tensile strength was also compared with uninjured native dorsal skin harvested from healthy mice. As expected, healed tissues at day 14 remained weaker than native dermis due to ongoing remodeling; however, STAT-treated wounds exhibited significantly closer recovery toward the native baseline than the suture control (Supplementary Fig. 19). Collectively, these results demonstrate that the STAT hydrogel holds significant potential as a next-generation material for effective wound closure and accelerated healing in clinical settings.

## Discussion

In this study, we developed a STAT hydrogel with strong tissue adhesion, antibacterial activity, and rapid hemostatic capability. These properties enable its application in diverse clinical scenarios. The incorporation of SA-DOPA introduced abundant catechol groups, which can be oxidized into o-quinone under ambient conditions. This transformation enables immediate covalent bonding with nucleophilic groups on tissue surfaces, thereby achieving strong adhesion without the need for additional activation steps. In parallel, the hydrogel’s TA component not only provided potent antibacterial effects by disrupting bacterial cell membranes but also promoted rapid blood clotting through protein aggregation and fibrin network formation. In vitro antibacterial assays confirmed effective inhibition of bacterial growth, while cytocompatibility and hemocompatibility tests verified that the hydrogel is safe for direct contact with living tissues and blood. These properties are essential for wound sealing materials, where infection control, tissue compatibility, and non-hemolytic behavior are critical prerequisites. The hydrogel’s hemostatic performance was validated in both small and large animal bleeding models. In a mouse liver puncture model, the hydrogel achieved rapid cessation of bleeding within seconds and significantly reduced total blood loss compared to gauze. In a rabbit massive bleeding model involving spleen incision and liver dissection, the hydrogel outperformed gauze by instantly sealing the bleeding surface and maintaining hemostasis over extensive wound areas, with minimal residual blood loss. Furthermore, the shear-thinning property of the hydrogel enabled precise extrusion-based printing and direct injection into irregular wound sites. In a murine dorsal skin incision model, the hydrogel effectively approximated wound edges and maintained a moist healing environment, resulting in faster and complete wound closure compared to conventional sutures, fibrin glue, and Zip band. Collectively, these findings demonstrate that the TA-based hydrogel combines strong wet adhesion, antibacterial activity, rapid hemostatic action, and wound healing in a single platform. By eliminating the need for lengthy pre-activation steps, the hydrogel uniquely integrates instant adhesion with multifunctionality. This makes it a versatile and effective next-generation bioadhesive for applications such as emergency bleeding control, gastrointestinal leak sealing, and accelerated tissue regeneration.

## Methods

### Materials

The chemicals were purchased and used without further purification. PVA (MW 89,000–98,000), TA, SA, DOPA, AA, borax, 2,2′-Azobis(2-methylpropionamidine) dihydrochloride, N,N′-Bis(acryloyl)cystamine were purchased from Sigma Aldrich, USA. EDC and NHS were purchased from Thermo Fisher Scientific, USA.

### Synthesis of SA-DOPA

To prepare SA-DOPA, 6.67 × 10^−^^5 ^M of SA was dissolved in 58 mL of PBS adjusted to pH 5.5 using approximately 18 µL of HCl. The solution was stirred with 0.051 M of EDC and 0.051 M of NHS at room temperature for 45 m. Subsequently, 0.108 M of DOPA was added, and the reaction was allowed to proceed overnight under a nitrogen atmosphere with continuous stirring. The resulting SA-DOPA was precipitated in ethanol and dialyzed for three days. The dialyzed product was then lyophilized to obtain the final SA-DOPA.

### Fabrication of the STAT Hydrogel

For hydrogel fabrication, a solution of poly(vinyl alcohol) (PVA, 18.18% w/v, 3.64 g in 20 mL DI water) was prepared, and SA–DOPA (50 mg, 0.05 g) was added to the solution. The mixture was stirred at 100 °C for 1 h. Subsequently, 2 mL of borax solution (2 wt%) was added, followed by tannic acid (TA, 9.09% w/v), and the solution was stirred for an additional 2 h. The resulting solution was cast onto a glass plate mold and frozen at −16 °C for 12 h, followed by thawing for 12 h at room temperature. The hydrogel was sequentially dried at 37 °C and then at 100 °C, each for 1 h. To incorporate a PAA network, the dried hydrogel was immersed in an acrylic acid (AA) precursor solution containing acrylic acid (30 wt%), N,N′-bis(acryloyl)cystamine (0.03 wt%), and 2,2′-azobis(2-methylpropionamidine) dihydrochloride (0.15 wt%) in DI water for 2 h. The hydrogel was then heated at 70 °C for 30 min to initiate polymerization. Finally, the hydrogel was washed three times with DI water and soaked overnight in DI water to complete the fabrication process.

### Borax-mediated control of catechol oxidation

To validate borax-mediated control of catechol oxidation, SA-DOPA solutions (1 mg mL⁻¹ in PBS) were incubated at 37 °C under three conditions: (i) without borax (static), (ii) with borax (5 mM, static), and (iii) with borax (5 mM) under continuous mixing (800 rpm). After incubation for 15 min in ambient air, UV–vis spectra (230–500 nm) were recorded. Quinone formation was quantified by extracting the characteristic absorbance values at ~280 nm (catechol) and ~400 nm (o-quinone band), and by calculating the A400/A280 ratio.

### Preparation of PVA hydrogel

PVA hydrogel was prepared using the freeze–thaw method. Briefly, 4000 mg of PVA was dissolved in 20 mL of DI water under constant stirring at 100 °C for 1 h until a clear homogeneous solution was obtained. The resulting solution was then subjected to a freeze-thaw cycle consisting of freezing at −20 °C for 12 h followed by thawing at room temperature for 4 h. The hydrogel obtained after one freeze–thaw cycle was used for subsequent experiments.

### Characterizations

Chemical characterization of the hydrogel was performed using X-ray Photoelectron Spectroscopy (XPS) system (K-alpha, Thermo Scientific, USA) and Fourier Transform Infrared Spectroscopy (FT-IR, Vertex 70, Bruker, USA). For XPS analysis, freeze-dried hydrogel samples were prepared, and a circular area with a diameter of 400 μm was examined using a spectrometer equipped with an Al K-α X-ray source. FT-IR spectra were recorded over the range of 4000 to 650 cm^−1^. The thermal stability and composition of the freeze-dried hydrogel samples were assessed using Thermogravimetric Analysis (TGA, SDT Q600, TA Instruments, USA). The analysis was conducted under a continuous nitrogen flow, with a heating rate of 10 °C/min, spanning a temperature range from 30 to 1400 °C. To evaluate the crosslinking efficiency of the hydrogel, a gel fraction test was conducted. Hydrogels were prepared with dimensions of 10 × 10 mm² and dried at 37 °C for 24 h. Once the hydrogels reached a stable minimum weight, the dried samples were weighed (W_o_) and then immersed in DI water for 24 h. The swollen samples were subsequently dried again at 37 °C for 24 h, and their masses were remeasured (W_e_). The gel fraction was calculated using the formula: gel fraction (%) = (W_e_/W_o_) × 100%. Additionally, a water retention test was carried out at room temperature with a humidity level of 55–60%. The weight of the hydrogel samples was recorded at various time points, and water retention was determined using the equation: water retention (%) = (W_s_–W_d_)/(W_e_-W_d_). Here, W_e_ and W_s_ represent the weights of the hydrogel at equilibrium and at specific time points, respectively, while W_d_ denotes the weight of the dried hydrogel.

### Amine blocking of porcine skin

To block free amine groups on the porcine skin surface, the porcine skin was treated with a 10 nM solution of sulfo-NHS-acetate (Thermo Scientific, USA) prepared in PBS. The porcine skin was immersed in the blocking solution and incubated for 1 h at room temperature. Following the incubation, the porcine skin was thoroughly washed with fresh PBS 3–5 times (5 min per cycle) to ensure the complete removal of any unreacted reagents.

### Adhesion force tests

The adhesion strength of the hydrogel to porcine skin was tested by performing a lap shear test (ASTM F2255) and a 180-degree peel test (ASTM F2256). To evaluate shear strength, samples with an adhesion area measuring 2.5 cm in width and 1 cm in length were prepared and subjected to a standard lap-shear test (ASTM F2255). The tests were conducted using a mechanical testing machine (MultiTest 2.5-DV, Mecmesin, UK) equipped with a 50 N load cell. A constant tensile speed of 50 mm min^−^^1^ was maintained throughout all tests. Shear strength was calculated by dividing the maximum force by the adhesion area. To evaluate the interfacial toughness, adhesive samples with a width of 2.5 cm were prepared and subjected to a 180-degree peel test (ASTM F2256). The tests were carried out using a mechanical testing machine (MultiTest 2.5-DV, Mecmesin, UK) equipped with a 50 N load cell. A consistent peel rate of 50 mm/min^−^^1^ was maintained throughout the tests. As the peeling process reached equilibrium, the force measured stabilized at a constant value. The interfacial toughness was calculated by dividing twice this constant force by the width of the sample (2.5 cm). A polyethylene terephthalate film (thickness 50 µm; Goodfellow) was used as a robust support for the tissue and hydrogel, applied with a cyanoacrylate adhesive (Krazy Glue). The cross-sectional view images of porcine skin and the hydrogel were observed using SEM (JSM-IT-500HR, JEOL, JAPAN).

### Sorbitol-triggered detachment of hydrogel

To evaluate the on-demand detachment of the hydrogel from the porcine skin, a 0.1 M solution of D-sorbitol (Sigma-Aldrich, USA) was prepared in PBS. A gauze pad was thoroughly saturated with the 0.1 M D-sorbitol solution and placed directly onto the hydrogel adhered to the porcine skin. After incubating for 3 min at room temperature to allow the sorbitol to diffuse into the hydrogel, the hydrogel was gently detached from the porcine skin surface.

### Mechanical characterizations

All tensile tests on the hydrogels were conducted using a mechanical testing machine (MultiTest 2.5-DV, Mecmesin, UK) equipped with a 50 N load cell, operating at a speed of 50 mm/min. The sample size was approximately 10 × 10 × 1 mm^3^. Tensile compression cyclic tests were conducted at a speed of 10 mm/min. Self-healing efficiency was calculated by comparing the ultimate tensile strengths of the samples before and after self-healing.

$${{\rm{Healing\; efficiency}}} \,=\, \frac{N^{\prime} }{N}\times 100\, (\%),$$

where N’ is the fracture stress before self-healing, and N is the fracture stress after self-healing. To investigate the rheological properties of the hydrogels, a rheometer (MCR 102 and MCR 702e, Anton Paar GmbH, Austria) was utilized. The hydrogels were placed on a parallel plate with a diameter of 25 mm, maintaining a gap of approximately 1 mm between the plates. The G′ and G″ were evaluated by performing temperature scans from 25 to 95 °C at a constant frequency of 10 rad/s and a shear strain of 1%. The shear-thinning behavior of the hydrogels was assessed at 95 °C by measuring the shear viscosity over a logarithmic range of shear rates from 0.1 to 100 rad/s. Furthermore, to examine the stress relaxation properties of the hydrogels, the stress was measured as a function of time while applying a constant strain of 15%. These rheological measurements provide valuable insights into the viscoelastic behavior and flow characteristics of the developed hydrogels.

### Ex vivo gastric perforation test

Fresh porcine stomachs were obtained from a local slaughterhouse and immediately transported to the laboratory on ice. The stomachs were carefully cleaned and rinsed with sterile saline solution. To establish a gastric perforation model, a 1 × 1 cm^2^ incision was created on the outer surface of each stomach using a surgical scalpel. For visualization and monitoring of the internal gastric environment throughout the experiment, a rigid endoscope (4 mm 0 Degree Hopkins Laparoscopic Rigid Scope, Karl Storz GmbH & Co. KG, Germany) coupled with a portable monitor (C-MAC Monitor 8404 ZX, Karl Storz GmbH & Co. KG, Germany) was utilized. The prepared STAT hydrogel (3 × 3 cm^2^) was carefully attached to a PC film (Thickness: 3 mm) for subsequent endoscopic application. The STAT hydrogel-laden PC film was grasped using forceps and carefully introduced into the stomach through the perforation site. Under endoscopic guidance, the STAT hydrogel was precisely positioned to cover the perforation. Subsequently, the PC film was gently removed using the forceps, leaving the STAT hydrogel adhered to the inner surface of the stomach. Following the deployment of the STAT hydrogel, the endoscope was employed to visually assess the adhesion and conformity of the STAT hydrogel to the perforation site. High-resolution images and videos were captured to document the hydrogel’s ability to effectively seal the gastric perforation.

### Long-term storage stability and anti-oxidation evaluation

To investigate the storage stability and prevent oxidative degradation of the STAT hydrogel, an oxygen-free storage system was employed. The hydrogel was placed in a 250 mL multi-neck flask, which was then hermetically sealed with rubber septa. Nitrogen (N_2_) gas was purged into the flask at a flow rate of 5 L/min for 1 min to displace the internal air (approximately 20 volume exchanges), ensuring an inert atmosphere. The flask was then completely sealed to maintain the oxygen-free condition, and the samples were stored for 7 days at room temperature. The stability of the stored hydrogels was evaluated by comparing their properties at day 0 and day 7. The anti-oxidation effect was quantified by measuring the normalized brightness of the hydrogels at different reaction times (0 to 20 min). Additionally, the preservation of adhesive performance after 7 days was assessed through lap shear tests using porcine skin to determine the adhesive strength.

### Standardized preparation of STAT hydrogel via automated mechanical kneading

To ensure structural homogeneity and batch-to-batch reproducibility, the STAT hydrogel was prepared using a custom-built automated mechanical kneading system (Supplementary Fig. 8A), replacing conventional manual manipulation. The preparation was conducted within a thermal chamber maintained at 37 °C to stabilize the crosslinking kinetics. The apparatus was programmed to execute a continuous stretching-and-folding motion following a circular trajectory with a diameter of 6 cm. The system operated at a frequency of 0.3 Hz (3 cycles per 10 seconds), which corresponds to a constant linear kneading speed of approximately 5.66 cm/s. This standardized mechanical stimulation provided uniform shear stress throughout the hydrogel matrix, ensuring a consistent distribution of crosslinks. All samples were processed under these identical spatio-temporal conditions to eliminate operator-dependent variability and to guarantee the reliability of the subsequent physical and adhesive characterizations.

### In vitro antibacterial assay

Prior to the experiments, all materials underwent sterilization in an autoclave at 121 °C for a duration of 15 m. The Gram-negative bacteria E. coli and the Gram-positive bacteria S. aureus were initially cultured in 10 mL of Luria Bertani (LB) broth within an incubator set at 37 °C for a period of 24 h. To ascertain the concentrations of the stock bacterial cultures, the plate counting method was employed, utilizing 3 M Petrifilm count plates. The inhibition zone assay was conducted using bacterial suspensions that were diluted to achieve a concentration of 10^5^ CFU mL^−1^. A 100 μL aliquot of the diluted bacterial suspension was evenly spread across the surface of an LB Agar High Salt Plate, followed by the placement of a hydrogel sample on the plate. The experimental groups were as follows: (1) negative control, (2) PVA, (3) PVA/SA-DOPA, (4) PVA/SA-DOPA/TA/Borax, and (5) STAT hydrogel. Each group of plates was incubated within a chamber maintained at 37 °C for a duration of 12 h. Following incubation, the diameter of the inhibition zone was measured using a ruler. The width of the inhibition zone was determined using the following equation:

$${{\rm{Inhibition}}}\; {{\rm{zone}}}\; {{\rm{width}}}=\frac{({d}_{2}-{d}_{1})}{2}\,({{\rm{mm}}})$$

where d_1_ represents the diameter of the hydrogel sample, and d_2_ represents the diameter of the antibacterial zone. All experiments were performed in quadruplicate.

### In vitro biocompatibility assessment

To investigate the biocompatibility and cellular response to the STAT hydrogel, 5 × 5 mm² samples were prepared. The STAT hydrogel samples were placed in a two-chamber transwell system (pore size: 8 μm; Corning Inc.) to enable co-culture with NIH-3T3 fibroblasts (CRL-1658, American Type Culture Collection, USA). The cells were seeded at a concentration of 0.5 × 10⁵ cells/mL in 6-well plates, each containing 1.5 mL of high-glucose Dulbecco’s Modified Eagle’s Medium (DMEM, 11965092, Gibco, USA) supplemented with 10% fetal bovine serum (A5670701, Gibco, USA) and 1% penicillin-streptomycin (15070063, Gibco, USA). The STAT hydrogel was placed in the upper transwell insert, while NIH-3T3 fibroblasts were cultured in the lower chamber, ensuring that cells were not in direct physical contact with the hydrogel but were exposed to soluble factors diffusing through the porous membrane. Two complementary assays were employed to assess cell viability: the Live/Dead assay (L3224, Invitrogen, USA) and the CCK-8 assay (Thermo Scientific, USA). For the Live/Dead assay, cells were stained according to the manufacturer’s instructions, and fluorescence images were captured using an inverted fluorescence microscope (IX81, Olympus, Japan) at 10× magnification. The relative fluorescence intensity was measured to evaluate differences in cell viability between day 1 and day 3. In the CCK-8 assay, cells were cultured for 1 and 3 days post-seeding. At each time point, 100 µL of CCK-8 solution was introduced into each well and incubated for 2 h at 37 °C. A microplate reader was used to measure the absorbance at 450 nm, which correlates with cell viability. The relative cell viability was determined and compared between the different time points. To assess cell morphology, the aspect ratio of the cells, defined as the ratio between the major and minor cell axes, was calculated. For this, ~50 cells were quantified per well from three random fields (*n* = 3 independent wells). Fluorescent staining techniques were used to visualize the cytoskeleton and cell nuclei. NIH-3T3 cells were stained with Alexa 594-conjugated phalloidin (Thermo Scientific, USA) to label the actin filaments and DAPI (Thermo Scientific, USA) to stain the cell nuclei. The cells were fixed with 4% paraformaldehyde for 15 m at room temperature, followed by permeabilization with 0.1% Triton X-100 in PBS for 5 m. The cells were then incubated with DAPI solution for 5 m at room temperature in the absence of light. After rinsing with PBS, fluorescence images were acquired using a confocal microscope (LSM 980, Carl Zeiss, Germany). The ImageJ/FIJI software was utilized for image analysis and quantification.

### Hemolysis Assay

The hemolytic potential of the hydrogel samples was evaluated using fresh horse blood (MB-H1880, Kisan Bio Inc., Korea). The blood was centrifuged at 300 x *g* for 10 m using a cryogenic centrifuge. The supernatant was then aspirated, and the remaining erythrocytes were washed three times with PBS. Hydrogel samples measuring 10 × 10 mm² were immersed in PBS for 72 h at 37 °C to obtain hydrogel-conditioned PBS. A volume of 0.1 mL of the centrifuged erythrocytes was added to 0.9 mL of the hydrogel-conditioned PBS. To assess hemolysis, the erythrocyte solutions were centrifuged at 13,000 x *g* for 15 m. The absorbance of the supernatants was measured at 540 nm using a spectrophotometer to quantify the extent of hemolysis induced by the hydrogel samples.

### In vitro hemostatic assays

Female Institute of Cancer Research (ICR) mice were anesthetized, and whole blood was collected. The collected blood was decalcified using sodium citrate. Prior to the experiment, the decalcified blood was treated with calcium chloride. A volume of 1 mL of the prepared blood was loaded onto a 10 × 10 mm² hydrogel placed in a plate well. To collect uncoagulated blood, 5 mL of DI water was added to the well. Subsequently, the absorbance of the DI water was measured at 540 nm using a spectrophotometer.

### In vitro platelet-rich plasma (PRP) interaction assay

Mouse whole blood was collected into tubes preloaded with 3.2% sodium citrate (blood:citrate = 9:1, v/v) and processed immediately at room temperature. To obtain platelet-rich plasma (PRP), anticoagulated blood was centrifuged at 200 × *g* for 10 min (no brake). The upper plasma layer was carefully aspirated without disturbing the buffy coat and transferred to a new tube as PRP. For all PRP-based assays, PRP was gently mixed by slow inversion prior to use to minimize unintended platelet activation. Square hydrogel specimens (0.5 × 0.5 cm²) of either PVA hydrogel or STAT were placed in a sterile plate. PRP (50 µL) was gently dropped onto the surface of each hydrogel and incubated under static conditions at 37 °C for 5 min in a humidified chamber. After incubation, the supernatant PRP was carefully recovered without disturbing the hydrogel surface, and the remaining platelet number in the recovered PRP was quantified by manual counting using a hemocytometer. Platelet counts were normalized to a PRP-only control incubated under identical conditions in the absence of hydrogel contact (defined as 100%).

### In vivo biocompatibility test

In vivo biocompatibility assessment to investigate the biocompatibility of the STAT hydrogels in a living system, an in vivo study was conducted using a subcutaneous implantation model in mice. Female ICR mice (7 weeks old) were obtained from a commercial breeder (Orient Bio Inc., South Korea) and acclimated for a week under semi-specific pathogen-free conditions with a controlled 12 h light/dark cycle. The animal study protocol was approved by the Institutional Animal Care and Use Committee (IACUC) at Yonsei University (approval number: IACUC-A-202407-1889-02), ensuring compliance with the guidelines for the humane care and use of laboratory animals. Surgical procedures were performed under general anesthesia induced by the intraperitoneal injection of a ketamine/xylazine cocktail. The dorsal region of each mouse was shaved and disinfected with povidone-iodine solution. A small incision (approximately 10 mm) was made on the left side of the back to create a subcutaneous pocket. The pre-sterilized hydrogel samples (10 × 10 × 1 mm^3^) were carefully inserted into the pockets, and the incisions were closed with absorbable sutures (Vicryl 5-0, Ethicon, USA). The mice were monitored daily for any signs of discomfort, inflammation, or wound dehiscence during the 7 days post-implantation period. At the end of the observation period, the mice were humanely euthanized, and the implanted hydrogels, along with the surrounding tissue,s were surgically harvested. The excised samples were immediately fixed in 4% paraformaldehyde for 24 h at ambient temperature to preserve the tissue morphology. Following fixation, the samples were processed using a standard histological technique, which involved dehydration, clearing, and infiltration with paraffin wax in an automated tissue processor (ASP300S, Leica Biosystems, Germany). The processed tissues were then embedded in paraffin blocks (Histo Core Arcadia, Leica Biosystems, Germany) for sectioning. Thin tissue sections (4 µm) were obtained using a rotary microtome (RM2255, Leica Biosystems, Germany) and mounted on glass microscope slides. To visualize the tissue morphology and assess the local tissue response, the sections were stained with three different histological stains: H&E for general tissue structure, TB for mast cells, and MT for collagen deposition. The stained sections were examined under a light microscope to evaluate the biocompatibility of the implanted hydrogels based on the presence of inflammatory cells, foreign body giant cells, and fibrous capsule formation around the implants.

### In vivo hemostatic efficacy of hydrogels in a mouse liver injury model

The hemostatic effects of the STAT hydrogel were examined in a mouse model with liver hemorrhage using a previously reported method^55^. All animal experiments were conducted in accordance with the guidelines of the Institutional Animal Care and Use Committee (IACUC) and were approved under the protocol number [approval number: IACUC-A-202407-1889-02]. Seven-week-old female ICR mice were obtained from Orient Bio Inc. and were anesthetized using a ketamine (100 mg/kg) and xylazine (40 mg/kg) mixture, administered via intramuscular injection. Analgesics, specifically ketoprofen at 1 mg/kg, were administered via subcutaneous injection to minimize pain and distress. An incision was made in the abdominal area, and sterilized filter papers were placed beneath the liver. Bleeding was induced using an 18G needle, and the damaged area was immediately covered with either gauze or STAT hydrogel. An untreated group was also included as a control. The blood-absorbed filter papers were collected at the 150 s time point. The collected filter papers were then weighed to measure the amount of blood. Humane endpoints were established, and mice were sacrificed using CO_2_ gas when necessary. The study design included randomization and blinding to reduce bias, ensuring that the allocation of treatments and the assessment of outcomes were conducted without prior knowledge of group assignments. After the experiment, the gauze and the STAT hydrogels were subjected to SEM (JSM-IT-500HR, JEOL, Japan) imaging to observe their morphology and interaction with the blood components.

### In vivo preclinical hemostatic model in rabbits‘ spleens and livers

An in vivo preclinical hemostatic model was established using rabbits’ spleens and livers to evaluate the hemostatic efficacy of the STAT hydrogels. The rabbit experiments were performed using specific pathogen-free (SPF) New Zealand White rabbits sourced from Dooyeol Biotech Co., Ltd. (Seoul, Republic of Korea). Upon arrival, animals were individually housed in stainless-steel cages and allowed a two-week adaptation period under the supervision of veterinary staff at the Teaching Hospital of Chungnam National University College of Veterinary Medicine, with ad libitum access to standard chow and water. At the designated experimental endpoint, animals were deeply anesthetized and humanely euthanized by slow intravenous infusion of 10% potassium chloride solution in accordance with approved institutional protocols. The liver and spleen of each rabbit were exposed through a midline laparotomy incision. In both the liver hemostasis model and the spleen hemorrhage model, a linear wound measuring 5 mm in length and 3 mm in depth was created using a scalpel. Following approximately one second of uncontrolled bleeding, gauze and the STAT hydrogels were applied to the bleeding wounds, and the amount of blood loss was measured. Blood loss was quantified by measuring the weight of the absorbed materials used during the procedure. After the experiment, the gauze, fibrin glue (Tisseel, Baxter, USA), TachoSil patch (Takeda, Japan), and the STAT hydrogels were subjected to SEM (JSM-IT-500HR, JEOL, Japan) imaging to observe their morphology and interaction with the blood components.

### 3D printing

The 3D printing was performed using an EZROBO-5GX printer equipped with an AD3300C dispenser (Iwashita, Japan), which included a nozzle and a temperature controller. The ink was heated to 65 °C, and a 400 μm nozzle was utilized. The designs for the printed structures were created using the Ez-EDITOR robot software.

### In vivo wound closure tests

For in vivo wound closure evaluation, the efficacy of syringe-type STAT hydrogels in facilitating wound closure and healing was investigated using a mouse skin incision model^77^. Following depilation and iodine sterilization of the mouse dorsum, incisions measuring 2.0 cm in length were created on the back of each experimental group. The STAT hydrogels were applied to seal the incisions, with traditional medical sutures, fibrin glue (Greenplast Q prefilled Syringe Kit, Green Gross, Korea), and Zip band (Pullband Suture Kit, SURGINUS, Korea) employed as control interventions. Digital images of the wound sites were captured on days 0, 1, 3, 5, 7, and 14 post-surgery. The mice were euthanized at 7 and 14 days post-operation. Adjacent skin tissues were fixed in 4% paraformaldehyde and subjected to H&E, TB, and MT staining. To assess remodeling, tissue segments in the shape of incision-centric rectangles (2 cm × 3 cm) were excised at different healing stages and underwent tensile testing. The entire experimental protocol was replicated three times to ensure the reliability of the results.

### Statistical analysis

Statistical analyses were carried out with GraphPad Prism (GraphPad Software Inc., USA) and Origin (OriginLab Corporation). All quantitative data are expressed as mean ± standard deviation (SD). Normality of data distributions was first confirmed by the Shapiro–Wilk test before applying parametric methods. When two independent groups were compared, a two-tailed unpaired Student’s t-test was used. For datasets involving three or more groups or repeated time-point measurements, two-way ANOVA with Tukey’s post hoc correction was employed. The number of biological replicates (*n*) and the statistical approach used for each experiment are specified in the respective figure legends. Differences were considered statistically significant at *p < 0.05, **p < 0.01, ***p < 0.001, and ****p < 0.0001; ns indicates no significant difference.

### Reporting summary

Further information on research design is available in the Nature Portfolio Reporting Summary linked to this article.

## Supplementary information

Supplementary Information

Descriptions of Additional Supplementary Files

Supplementary Movie 1

Supplementary Movie 2

Supplementary Movie 3

Supplementary Movie 4

Supplementary Movie 5

Reporting Summary

Transparent Peer Review file

## Source data

Source data

## Data Availability

The data generated in this study are provided within the article and its supplementary files. Any additional requests for information can be directed to and will be fulfilled by the corresponding author. Source data are provided with this paper.
