# Supplementary material for: Quinone-mediated, tissue-adaptive double-network hydrogel for instant hemostasis and wet-tissue adhesion
Source: Nat Commun. 2026 Apr 22;17:5526. doi: 10.1038/s41467-026-72068-6 (PMC13287715; doi:10.1038/s41467-026-72068-6)
Supplement: Supplementary file 1 — Supplementary Information [file 41467_2026_72068_MOESM1_ESM.pdf]

Supporting Information

Quinone-Mediated, Tissue-Adaptive Double-Network Hydrogel for Instant Hemostasis and Wet-Tissue Adhesion

Tae Young Kim[1,†], Kayoung Son[1,†], Chang-Hwan Moon[2], Keun-Young Yook[1], Soo A Kim[1], Hyein Ham[1], Yurim Lee[1], Soo In Lee[1], Yejin Jo[1], Yunlong Yu[3], Dae-Hyun Kim[4], Jungmok Seo[1]*

[1]School of Electrical and Electronic Engineering, Yonsei University, 50 Yonsei-ro, Seodaemun-gu, Seoul 03722, Republic of Korea

[2]Department of Veterinary Surgery, College of Veterinary Medicine, Gyeongsang National University, 501, Jinjudae-ro, Jinju-si Gyeongsangnam-do, 52828 Republic of Korea

[3]Institute of Burn Research, Southwest Hospital, Army Medical University (Third Military Medical University), Chongqing 400038, P. R. China

[4]Department of Veterinary Surgery, Chungnam National University College of Veterinary Medicine, 99, Daehak-ro, Yuseong-gu, Daejeon, 34134 Republic of Korea

Correspondence should be addressed to J.S. (E-mail: jungmok.seo@yonsei.ac.kr).

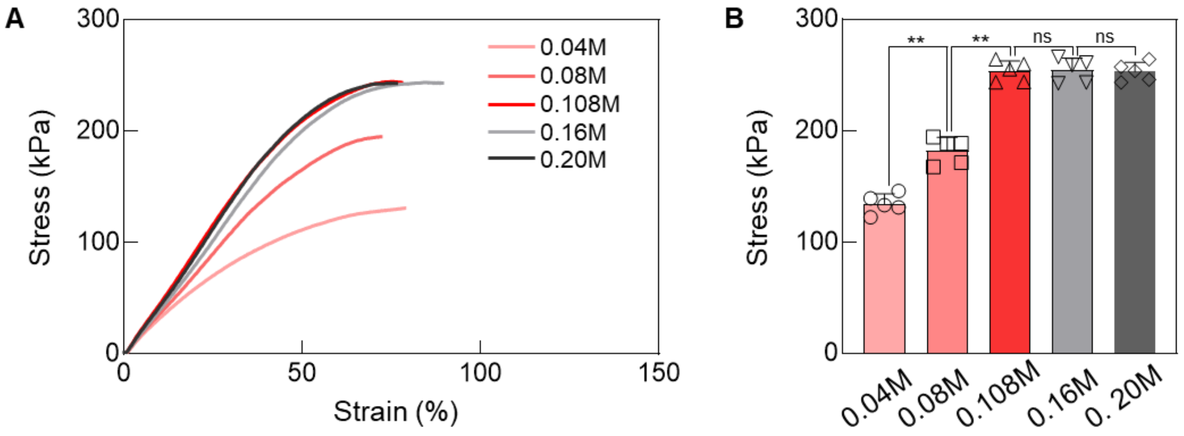

Fig. S1. Optimization of SA-DOPA concentration for STAT hydrogel formation. (A) Representative compressive stress-strain curves of hydrogels prepared with increasing SA-DOPA concentrations. (B) Comparison of maximum compressive stress as a function of SA-DOPA content, showing saturation beyond 0.108 M (n=5: n is the sample size for each group). Data are presented as mean ± SEM. Statistical analysis was performed using a two-sided Student's t-test. *(*P < 0.05, **P < 0.01, ***P < 0.001, and ****P < 0.0001). ns, not significant.*

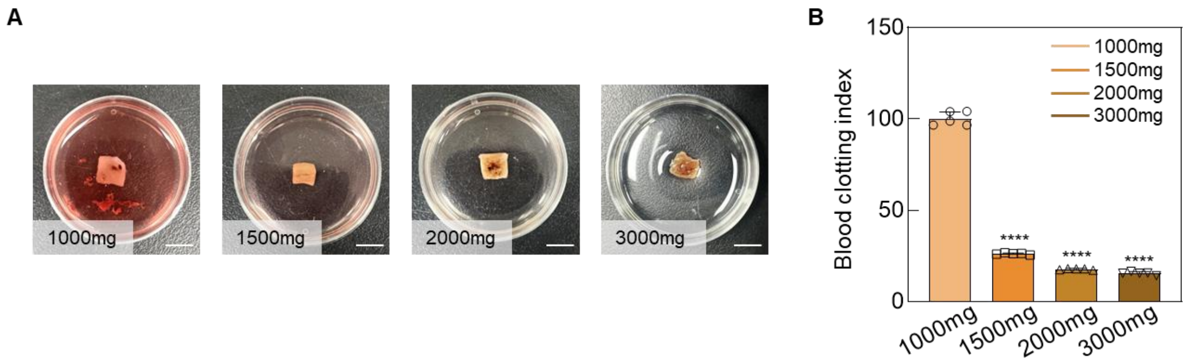

Fig. S2. Optimization of TA concentration for hemostatic performance. (A) Representative photographs of blood coagulation behavior on hydrogels containing increasing TA amounts. (B) Blood clotting index (BCI) as a function of TA dosage, showing saturated coagulation enhancement beyond 2000 mg (n=5: n is the sample size for each group). Data are presented as mean ± SEM. Statistical analysis was performed using a two-sided Student's t-test. *(*P < 0.05, **P < 0.01, ***P < 0.001, and ****P < 0.0001). ns, not significant.*

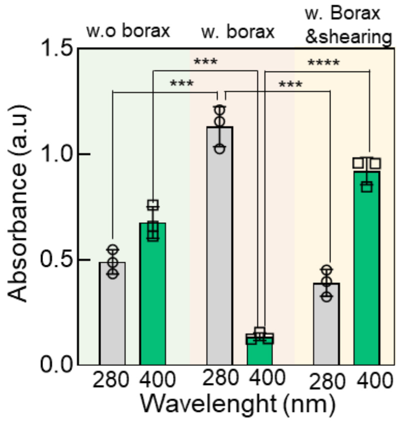

Fig. S3. UV–vis analysis of catechol oxidation in SA-DOPA hydrogels prepared with or without borax (n=3: n is the sample size for each group). Data are presented as mean ± SEM. Statistical analysis was performed using a two-sided Student's t-test.

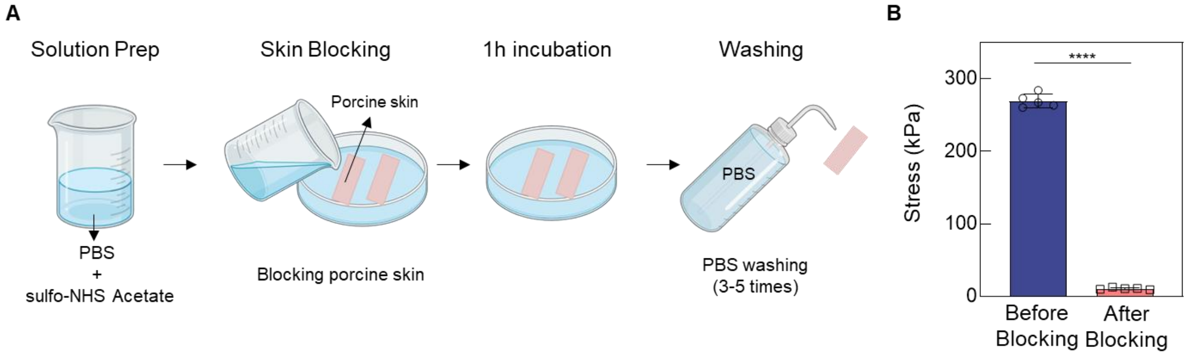

Fig. S4. Tissue amine functional group blocking assay for adhesion mechanism. (A) Schematic of sulfo-NHS acetate pre-treatment to block surface amines on porcine skin prior to hydrogel application. Created using Adobe Illustrator. (B) Quantification of STAT adhesion retention after PBS rinsing, showing significantly reduced adhesion on amine-blocked tissue (n=5: n is the sample size for each group). Data are presented as mean ± SEM. Statistical analysis was performed using a two-sided Student's t-test. *(*P < 0.05, **P < 0.01, ***P < 0.001, and ****P < 0.0001). ns, not significant*

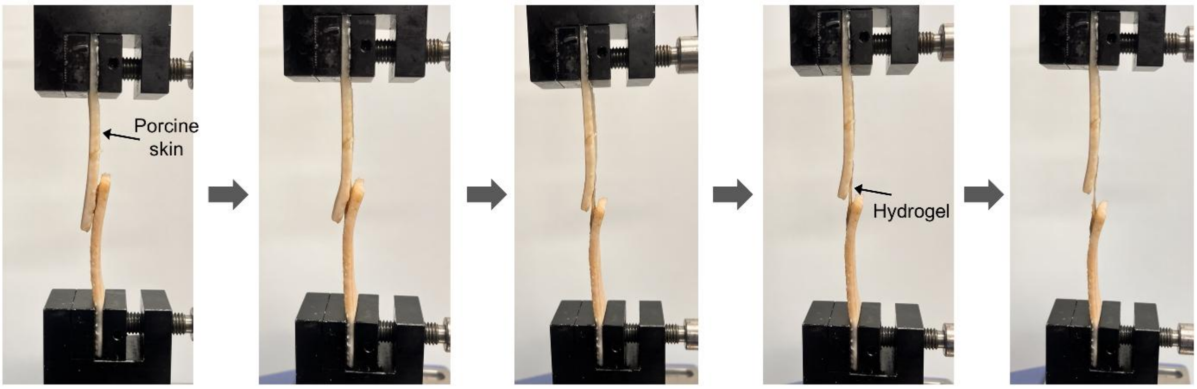

Fig. S5. Sequential image showing photographs of the lap shear test of the STAT hydrogel.

A B

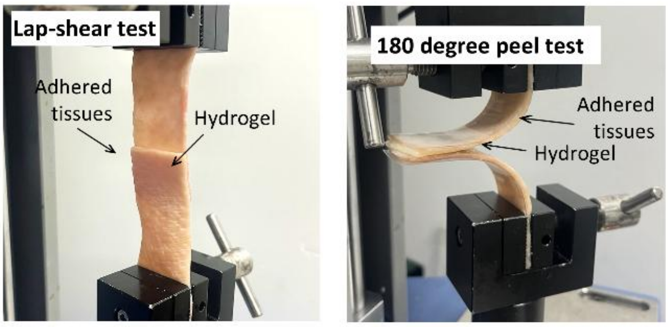

Fig. S6. The STAT hydrogel adhesion testing on porcine skin. (A) Configuration for lap shear test (B) Configuration for 180-degree peel test.

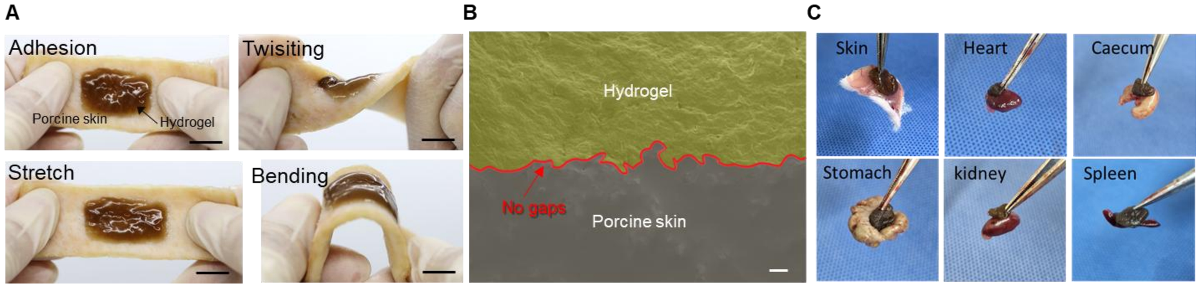

Fig. S7. The STAT hydrogel adhesion on porcine skin and mouse organs. (A) Photographs of the STAT hydrogel conformally attached to a porcine skin (scale bars: 1 cm) (B) SEM image of the STAT hydrogel adhered to a porcine skin surface (scale bar: 50 µm) (C) Optical image of holding mouse organs adhered to the STAT hydrogel.

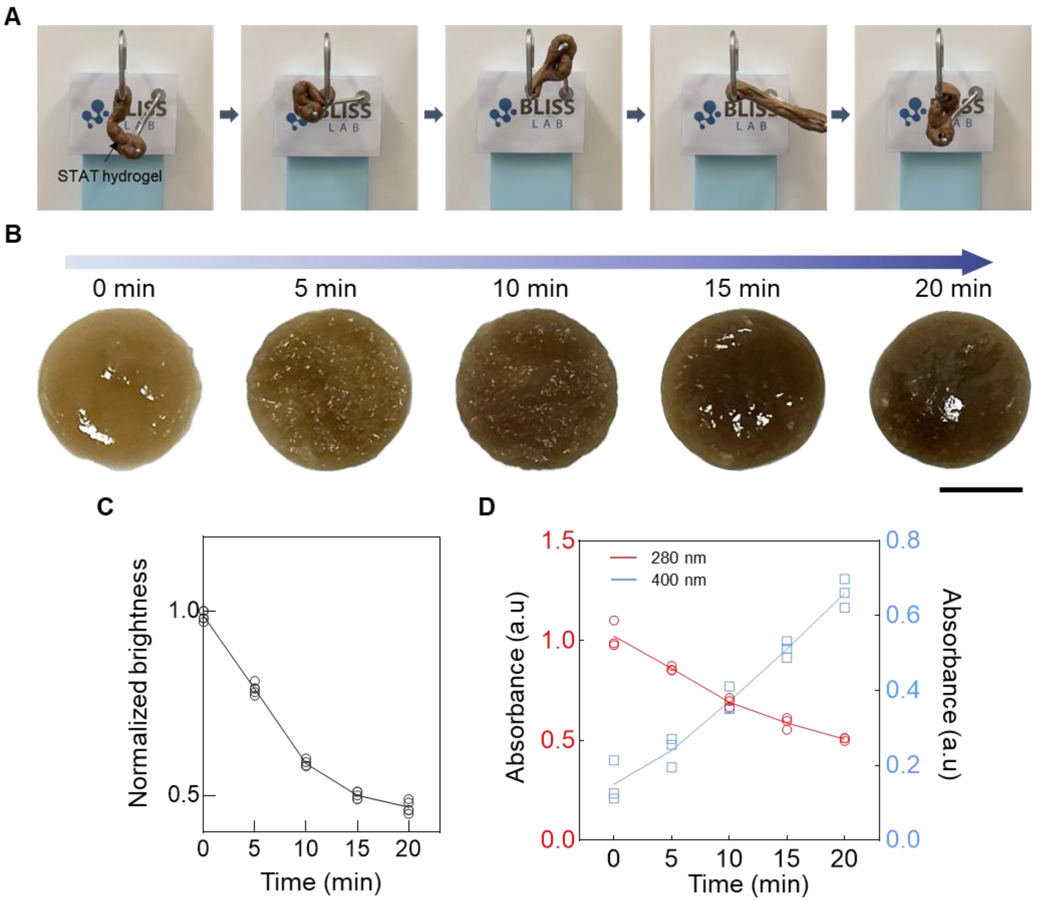

Fig. S8. Time-dependent catechol oxidation during hydrogel mixing. (A) Representative photograph image of mechanical mixing. (B) Representative photographs showing progressive color darkening of the hydrogel as a function of mixing time, indicating gradual oxidation of catechol groups. (C) Quantification of hydrogel color intensity over mixing duration, confirming a time-dependent increase in oxidative conversion (n=5: n is the sample size for each group). (D) UV-vis absorbance spectra of the hydrogel solution collected at different mixing times, demonstrating the emergence of characteristic oxidation-associated peaks, consistent with catechol-to-quinone transformation during mixing (n=3: n is the sample size for each group). *(*P < 0.05, **P < 0.01, ***P < 0.001, and ****P < 0.0001). ns, not significant.*

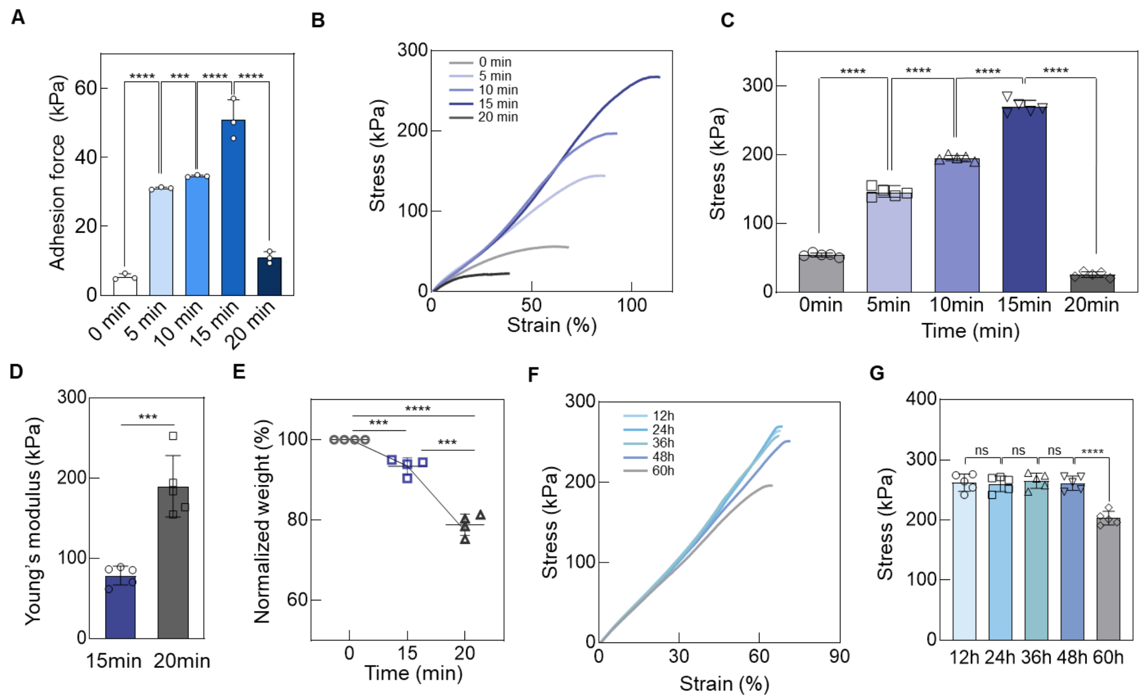

Fig. S9. Oxidation-driven mechanical evolution of STAT hydrogels during mixing and storage. (A) Time-dependent adhesion force of STAT hydrogels measured by rheometry (n=3: n is the sample size for each group). Data are presented as mean ± SEM. Statistical analysis was performed using a two-sided Student's t-test. (B) Representative stress-strain curves of STAT hydrogels collected at different mixing times (n=5: n is the sample size for each group). (C) Quantitative analysis of mechanical performance as a function of mixing time (n=5: n is the sample size for each group). Data are presented as mean ± SEM. Statistical analysis was performed using a two-sided Student's t-test. (D) Young's modulus of STAT hydrogels measured under varying mixing conditions (n=5: n is the sample size for each group). Data are presented as mean ± SEM. Statistical analysis was performed using a two-sided Student's t-test. (E) Hydrogel weight change during continuous mixing, revealing gradual mass reduction due to water evaporation (n=4: n is the sample size for each group). Data are presented as mean ± SEM. Statistical analysis was performed using a two-sided Student's t-test. (F) Stress-strain profiles of STAT hydrogels (15 min mixing) after storage at room temperature for different

durations. (G) Quantification of mechanical properties following room-temperature storage (n=5: n is the sample size for each group). Data are presented as mean ± SEM. Statistical analysis was performed using a two-sided Student's t-test. *(*P < 0.05, **P < 0.01, ***P < 0.001, and ****P < 0.0001). ns, not significant.*

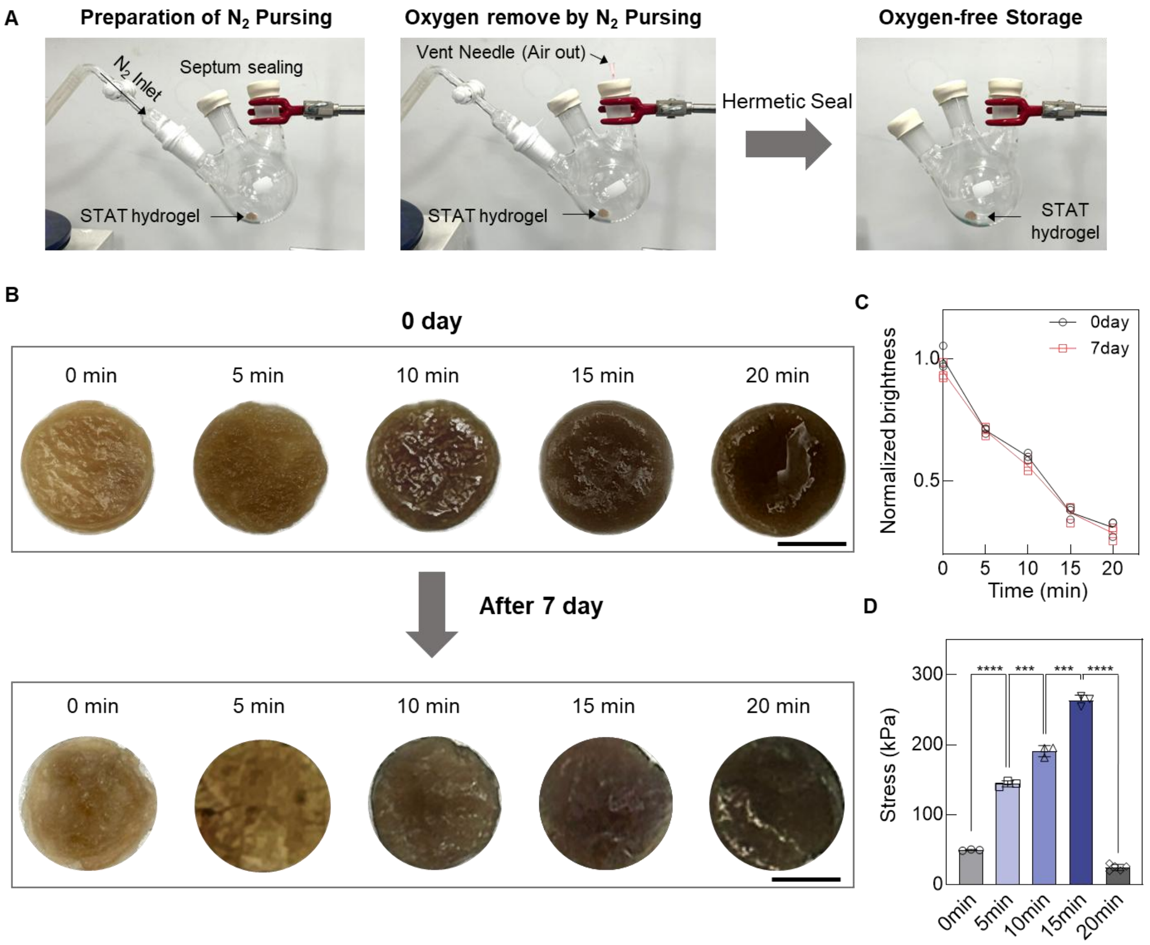

Fig. S10. Suppression of hydrogel oxidation under N_2 storage. (A) Photographs showing N_2 purging and sealed storage of STAT hydrogels to limit oxygen exposure. (B) Representative images of STAT hydrogels after 1 week of storage under different conditions, revealing reduced color darkening under N_2 atmosphere. (C) Quantification of hydrogel color intensity after storage, confirming attenuated oxidation with N_2 protection (n=3: n is the sample size for each group). (D) Mechanical/adhesion performance measured after 1 week of storage, demonstrating preservation of hydrogel properties under oxygen-limited conditions (n=3: n is the sample size for each group). Data are presented as mean ± SEM. Statistical analysis was performed using a two-sided Student's t-test. (*$P < 0.05$, **$P < 0.01$, ***$P < 0.001$, and ****$P < 0.0001$). ns, not significant.

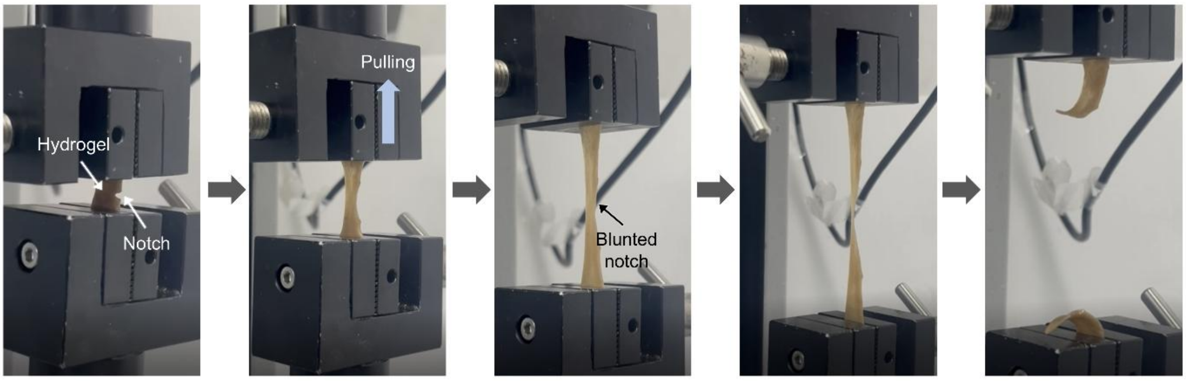

Fig. S11. Sequential image showing elongation of the notch-introduced STAT hydrogel.

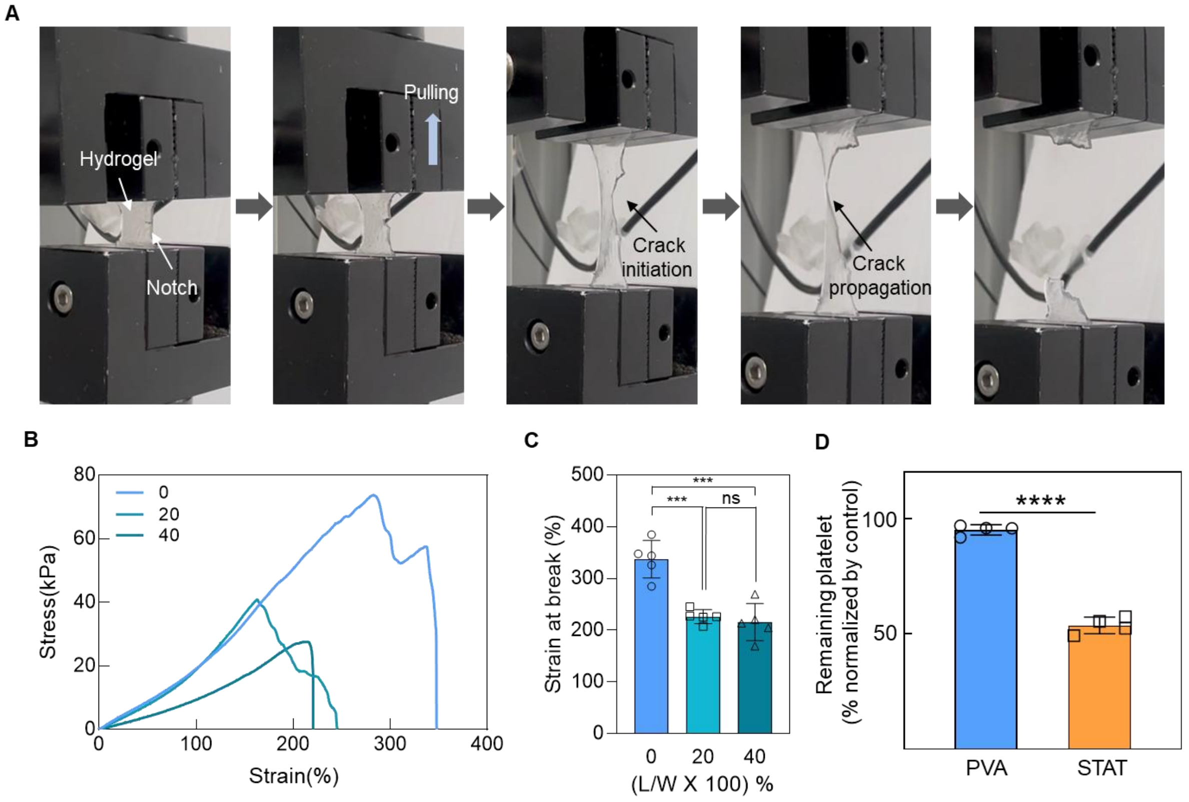

Fig. S12. Notch tolerance and platelet association of STAT hydrogels. (A)Sequential image showing elongation of the notch-introduced PVA hydrogel. (B) Strain at break values of the PVA hydrogel with varying notch lengths. (C) Statistical analysis of the strain-at-break of the notch-introduced PVA hydrogel (n=5: n is the sample size for each group). Data are presented as mean ± SEM. Statistical analysis was performed using a two-sided Student's t-test. (D) Remaining platelets in PRP supernatant after 5 min contact with PVA or STAT hydrogels. STAT exhibited significantly enhanced platelet association compared with PVA (n=4: n is the sample size for each group). Data are presented as mean ± SEM. Statistical analysis was performed using a two-sided Student's t-test. *(*P < 0.05, **P < 0.01, ***P < 0.001, and ****P < 0.0001). ns, not significant.*

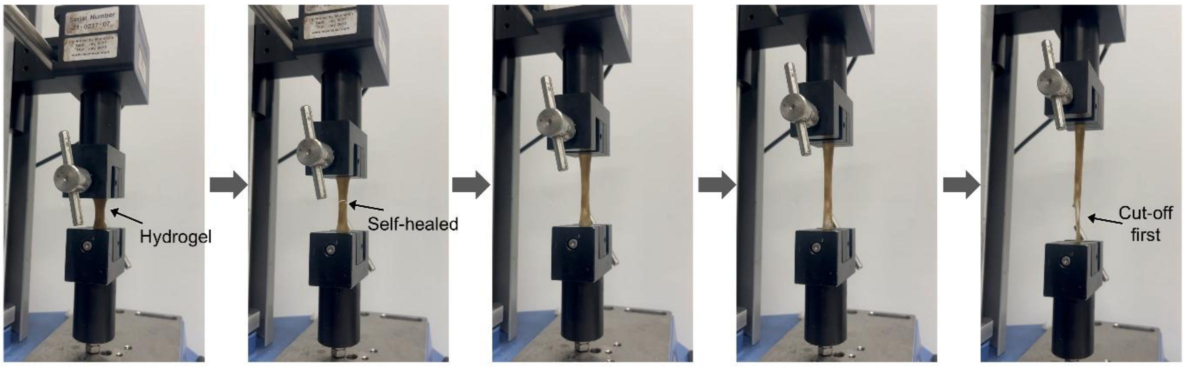

Fig. S13. Sequential image showing self-healing test of the STAT hydrogel.

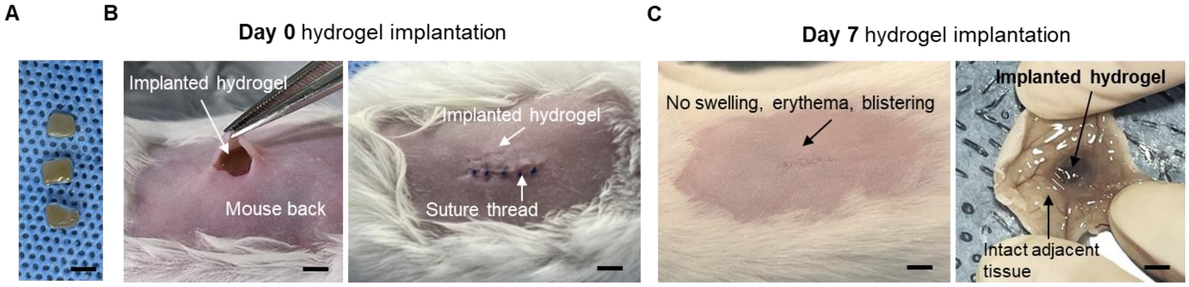

Fig. S14. Optical images of the STAT hydrogel implanted in a mouse's subcutaneous tissue. (A) Hydrogel samples prepared for implantation. (B) Optical photographs of the STAT hydrogel implantation procedure. (C) Seven days post-implantation, showing subcutaneous skin and suture site near the STAT hydrogel (Scale bars: 5 mm).

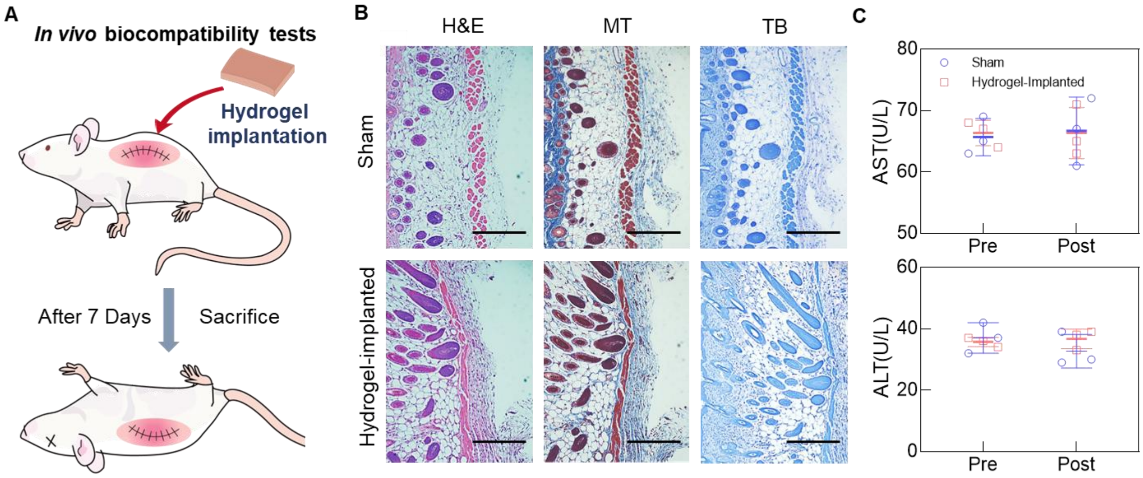

Fig. S15. ***In vivo*** **biocompatibility tests of the STAT hydrogel.** (A) Schematics of subcutaneous implantation of the STAT hydrogel. Created using Adobe Illustrator. (B) Histological analyses after 7 days of implantation: H&E, nuclei were stained blue, and the cell cytoplasm was stained pink; MT, collagen was stained in blue, nuclei were stained in black, and the cytoplasm was stained in red; TB, inflammatory cell responses were assessed, with inflammatory cells stained in blue (scale bars: 100 μm). (C) Hepatotoxicity evaluation with measurement of serum level of ALT and AST (n=3: n is the sample size for each group).

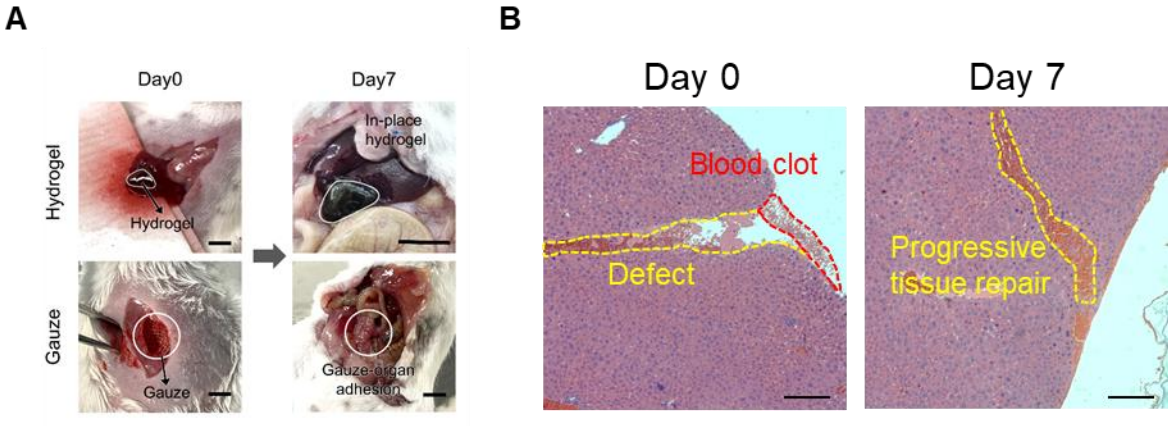

Fig. S16. Photographs showing the STAT hydrogel and the gauze attached to liver tissue. (A) Comparison of the STAT hydrogel and the gauze on the liver bleeding site on Day 0 and Day 7 (scale bar: 5 mm). (B) H&E staining images of the repaired skin after 7 days (scale bar: 100 μm).

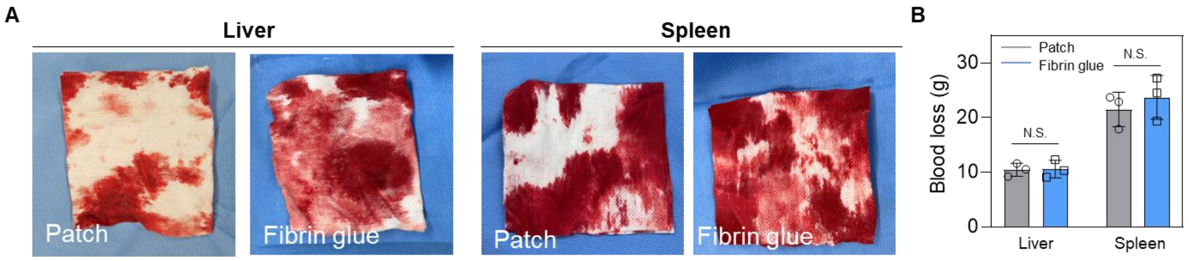

Fig. S17. Hemostatic performance in a rabbit model. (A) Photographs showing blood loss after hemostasis. (**B**) Accumulated blood loss comparisons among liver and spleen treatments (n=3: n is the sample size for each group). Data are presented as mean ± SEM. Statistical analysis was performed using a two-sided Student's t-test. *(*P < 0.05, **P < 0.01, ***P < 0.001, and ****P < 0.0001). ns, not significant.*

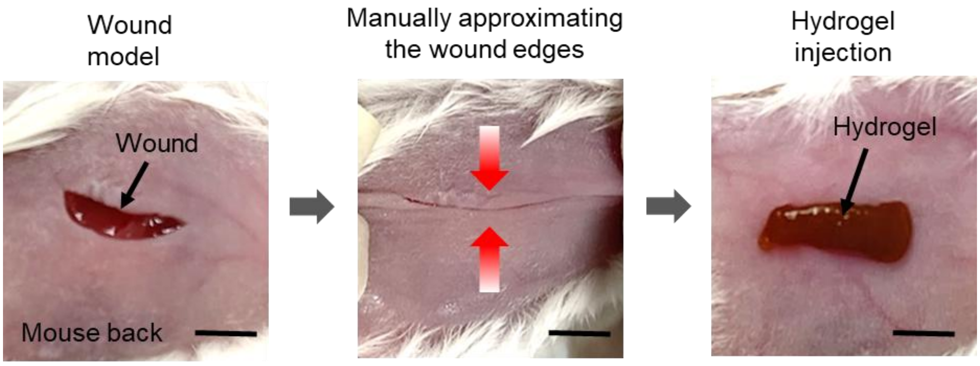

Fig. S18. Wound closure process using syringe-type STAT hydrogel application. The method of wound closure using syringe-type STAT hydrogel.

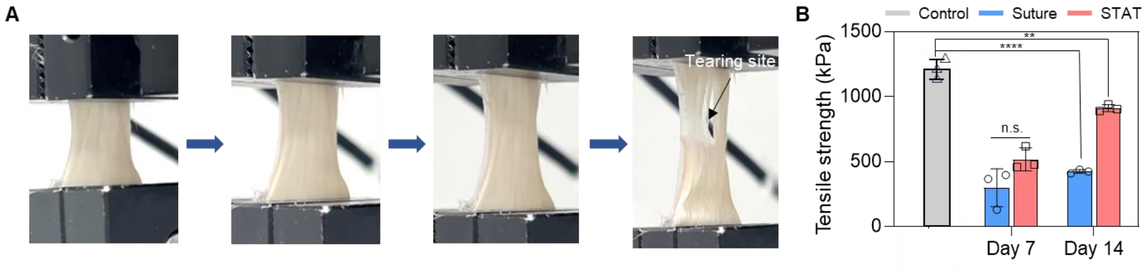

Fig. S19. Tensile strength recovery of healed dermal tissue. (A) Representative photographs of tensile testing of excised dorsal skin samples. (B) Tensile strength comparison of uninjured native dermis and healed wounds treated with suture or STAT at day 14 (n=3: n is the sample size for each group). Data are presented as mean ± SEM. Statistical analysis was performed using a two-sided Student's t-test. *(*P < 0.05, **P < 0.01, ***P < 0.001, and ****P < 0.0001). ns, not significant.*

Table

Material	Adhesion Strength [kPa] (shear test)	Hemostatic Time [s]	Printability	Special feature	Refs
STAT hydrogel	273	~ 10 s	○	Quinone-mediated instant wet-tissue adhesion (no EDC/NHS, no external activation)	
DNAgel (DNA hydrogel band-aid)	5.29 ± 0.80	NR (time-to-hemostasis not reported as a single 'seconds' endpoint)	X	NETs-mimicking ultra-swelling hemostatic hydrogel (platelet/RBC enrichment)	1
PVA/TA/PAA hydrogel (TA-incorporated PVA/PAA double-network hydrogel patch)	31	~ 10 s	○	Nonswellable, self-healable tough DN hydrogel with conformal covalent tissue adhesion	2
SSAD-Patch (multilayer hydrophobic/hydrophilic patch)	22.2 ±2.4	NR (reported as sealing within 10 s; not hemostasis-time endpoint)	X	Pressure-triggered wet tissue sealing patch for rapid hemorrhage control	3
ES gel (ε-polylysine + Tetra-PEG-SS injectable adhesive hydrogel)	29.4 ± 7.4	~ 8 s	X	Ultrafast, antibacterial, coagulation-independent hemostatic gel (large-animal/anticoagulated)	4

Supplementary Table 1. Rapid Hemostasis and Bioadhesive Hydrogel for Wound Healing

Reference

1. Ye R, et al. Neutrophil extracellular traps-inspired DNA hydrogel for wound hemostatic adjuvant. Nature Communications 15, 5557 (2024).

2. Park J, et al. A mechanically resilient and tissue-conformable hydrogel with hemostatic and antibacterial capabilities for wound care. Advanced Science 10, 2303651 (2023).

3. Liu S, et al. A nature-derived, hetero-structured, pro-healing bioadhesive patch for high-performance sealing of wet tissues. Advanced Materials 36, 2309774 (2024).

4. Yang Y, et al. An injectable hydrogel with ultrahigh burst pressure and innate antibacterial activity for emergency hemostasis and wound repair. Advanced Materials 36, 2404811 (2024).
