# Supplementary material for: Quinone-mediated, tissue-adaptive double-network hydrogel for instant hemostasis and wet-tissue adhesion
Source: Nat Commun. 2026 Apr 22;17:5526. doi: 10.1038/s41467-026-72068-6 (PMC13287715; doi:10.1038/s41467-026-72068-6)
Supplement: Supplementary file 2 — Descriptions of Additional Supplementary Files [file 41467_2026_72068_MOESM2_ESM.pdf]

Description of Additional Supplementary Files

Supplementary Movie 1. Quinone-mediated tissue adhesion of oxidized STAT hydrogel.

Video demonstrated enhanced adhesion of oxidized STAT hydrogel to porcine skin compared to pre-oxidized hydrogel, supporting quinone-mediated covalent bonding at the tissue interface.

Supplementary Movie 2. Sorbitol-triggered detachment of STAT hydrogel.

Video demonstrating controlled reduction of adhesion following application of a sorbitol-based triggering solution, enabling gentle detachment without disruption of the tissue surface.

Supplementary Movie 3. Endoscopic application of STAT hydrogel.

Demonstration of hydrogel delivery and attachment to an artificial gastric perforation in an ex vivo porcine stomach model under real-time endoscopic guidance.

Supplementary Movie 4. Hemostatic performance in a rabbit spleen injury model.

Demonstration of rapid hemostasis following application of the STAT hydrogel to a high-flow spleen injury, compared with gauze, fibrin glue, and a TachoSil patch.

Supplementary Movie 5. Hemostatic performance in a rabbit liver dissection model.

Demonstration of effective sealing and bleeding control by the STAT hydrogel in a large-area liver injury model, compared with gauze, fibrin glue, and a TachoSil patch.
