# Supplementary material for: Quinone-mediated, tissue-adaptive double-network hydrogel for instant hemostasis and wet-tissue adhesion
Source: Nat Commun. 2026 Apr 22;17:5526. doi: 10.1038/s41467-026-72068-6 (PMC13287715; doi:10.1038/s41467-026-72068-6)
Supplement: Supplementary file 8 — Reporting Summary [file 41467_2026_72068_MOESM8_ESM.pdf]

nature portfolio

Corresponding author(s): Jungmok Seo

Last updated by author(s): Mar 8, 2026

Reporting Summary

Nature Portfolio wishes to improve the reproducibility of the work that we publish. This form provides structure for consistency and transparency in reporting. For further information on Nature Portfolio policies, see our Editorial Policies and the Editorial Policy Checklist.

Statistics

For all statistical analyses, confirm that the following items are present in the figure legend, table legend, main text, or Methods section.

n/a	Confirmed	
☐	☒	The exact sample size (*n*) for each experimental group/condition, given as a discrete number and unit of measurement
☒	☐	A statement on whether measurements were taken from distinct samples or whether the same sample was measured repeatedly
☐	☒	The statistical test(s) used AND whether they are one- or two-sided<br>*Only common tests should be described solely by name; describe more complex techniques in the Methods section.*
☒	☐	A description of all covariates tested
☐	☒	A description of any assumptions or corrections, such as tests of normality and adjustment for multiple comparisons
☐	☒	A full description of the statistical parameters including central tendency (e.g. means) or other basic estimates (e.g. regression coefficient) AND variation (e.g. standard deviation) or associated estimates of uncertainty (e.g. confidence intervals)
☒	☐	For null hypothesis testing, the test statistic (e.g. *F*, *t*, *r*) with confidence intervals, effect sizes, degrees of freedom and *P* value noted<br>*Give P values as exact values whenever suitable.*
☒	☐	For Bayesian analysis, information on the choice of priors and Markov chain Monte Carlo settings
☒	☐	For hierarchical and complex designs, identification of the appropriate level for tests and full reporting of outcomes
☒	☐	Estimates of effect sizes (e.g. Cohen's *d*, Pearson's *r*), indicating how they were calculated

Our web collection on statistics for biologists contains articles on many of the points above.

Software and code

Policy information about availability of computer code

Data collection	XPS and FT-IR were used for chemical characterization; TGA was used to analyze the thermal stability and composition of the hydrogel; MultiTest 2.5-DV was used for mechanical characterization of the hydrogel; SEM confirmed the adhesive properties of the hydrogel; A rheometer was used to analyze the rheological properties of the hydrogels; A SpectraMax plate reader was used to analyze cell viability; Biorender.com was used to create schematic illustrations;
Data analysis	Adobe Illustrator; Autodesk Fusion 360 ; GraphPad Prism 8.0.1;

For manuscripts utilizing custom algorithms or software that are central to the research but not yet described in published literature, software must be made available to editors and reviewers. We strongly encourage code deposition in a community repository (e.g. GitHub). See the Nature Portfolio guidelines for submitting code & software for further information.

Data

Policy information about availability of data

All manuscripts must include a data availability statement. This statement should provide the following information, where applicable:

- Accession codes, unique identifiers, or web links for publicly available datasets
- A description of any restrictions on data availability
- For clinical datasets or third party data, please ensure that the statement adheres to our policy

Source data are provided with this paper. Any additional requests for information can be directed to the corresponding authors

Research involving human participants, their data, or biological material

Policy information about studies with human participants or human data. See also policy information about sex, gender (identity/presentation), and sexual orientation and race, ethnicity and racism.

Reporting on sex and gender	N/A
Reporting on race, ethnicity, or other socially relevant groupings	N/A
Population characteristics	N/A
Recruitment	N/A
Ethics oversight	N/A

Note that full information on the approval of the study protocol must also be provided in the manuscript.

Field-specific reporting

Please select the one below that is the best fit for your research. If you are not sure, read the appropriate sections before making your selection.

☒ Life sciences ☐ Behavioural & social sciences ☐ Ecological, evolutionary & environmental sciences

For a reference copy of the document with all sections, see nature.com/documents/nr-reporting-summary-flat.pdf

Life sciences study design

All studies must disclose on these points even when the disclosure is negative.

Sample size	Sample sizes are clearly reported in the figure legends and are generally n=3. In the animal model, experiments were conducted with n=3 independent replicates.
Data exclusions	No data were excluded
Replication	Replication attempts succeeded on at least two separate occasions
Randomization	The samples were assigned randomly to experimental groups
Blinding	The clinical data were collected blindly

Reporting for specific materials, systems and methods

We require information from authors about some types of materials, experimental systems and methods used in many studies. Here, indicate whether each material, system or method listed is relevant to your study. If you are not sure if a list item applies to your research, read the appropriate section before selecting a response.

Materials & experimental systems

n/a	Involved in the study	
☒	☐	Antibodies
☐	☒	Eukaryotic cell lines
☒	☐	Palaeontology and archaeology
☐	☒	Animals and other organisms
☒	☐	Clinical data
☒	☐	Dual use research of concern
☒	☐	Plants

Methods

n/a	Involved in the study	
☒	☐	ChIP-seq
☒	☐	Flow cytometry
☒	☐	MRI-based neuroimaging

Eukaryotic cell lines

Policy information about cell lines and Sex and Gender in Research

Cell line source(s)	The NIH-3T3 fibroblasts used in the experiment were obtained from the American Type Culture Collection (ATCC), catalog

Cell line source(s)	number CRL-1658, in the USA.
Authentication	Although the document does not explicitly mention cell line authentication, it is generally performed using methods such as Short Tandem Repeat (STR) analysis. The NIH-3T3 cell line used in the experiment was obtained from ATCC, so it is likely that it underwent ATCC's authentication procedures. ATCC is a reputable source known for its rigorous quality control and authentication processes, which ensure the reliability and validity of the cell lines they provide.
Mycoplasma contamination	Declare that the cell line was not test mycoplasma contamination.
Commonly misidentified lines (See ICLAC register)	No Commonly misidentified cell lines were used in the study.

Animals and other research organisms

Policy information about studies involving animals; ARRIVE guidelines recommended for reporting animal research, and Sex and Gender in Research

Laboratory animals	Mouse Liver Injury Model: The study used seven-week-old female ICR mice obtained from Orient Bio Inc. The mice were anesthetized with a ketamine and xylazine mixture and received analgesics to minimize pain.[approval number: IACUC-A-202407-1889-02] Rabbit Spleen and Liver Model: SPF New Zealand White Rabbits were used, obtained from Dooyel Biotech Co., Ltd. The rabbits were housed individually and acclimatized for two weeks before the experiment.[approval number: 202509A-CNU-188]
Wild animals	N/A
Reporting on sex	The study results apply only to female mice. In the study design, only female mice were used, and gender information for rabbits was not collected. This is because the primary focus of the research was on the hemostatic efficacy of STAT hydrogel rather than gender differences. Therefore, a gender-based analysis was not conducted, and this lack of analysis can be justified for the stated reason. The report summary provides data only for mice, and gender information for rabbits is not included.
Field-collected samples	N/A
Ethics oversight	Animal experiments were conducted in accordance with the guidelines set by the Institutional Animal Care and Use Committees (IACUC) at both Yonsei University College of Medicine [approval number: IACUC-A-202407-1889-02] and the Veterinary Medical Teaching Hospital of Chungnam National University[approval number: 202509A-CNU-188].

Note that full information on the approval of the study protocol must also be provided in the manuscript.

Plants

Seed stocks	N/A
Novel plant genotypes	N/A
Authentication	N/A
