# Supplementary material for: Quinone-mediated, tissue-adaptive double-network hydrogel for instant hemostasis and wet-tissue adhesion
Source: Nat Commun. 2026 Apr 22;17:5526. doi: 10.1038/s41467-026-72068-6 (PMC13287715; doi:10.1038/s41467-026-72068-6)
Supplement: Supplementary file 9 — Transparent Peer Review file [file 41467_2026_72068_MOESM9_ESM.pdf]

nature portfolio

Peer Review File

Quinone-Mediated, Tissue-Adaptive Double-Network Hydrogel for Instant Hemostasis and Wet-Tissue Adhesion

Corresponding Author: Professor Jungmok Seo

This file contains all reviewer reports in order by version, followed by all author rebuttals in order by version.

Version 0:

Reviewer comments:

Reviewer #1

(Remarks to the Author)
The manuscript presents a novel high-adhesion hydrogel designed for rapid hemostasis, demonstrating tissue-compatible modulus, excellent adhesion, high strength, and outstanding biocompatibility and antibacterial properties. This hydrogel also exhibits shear-thinning behavior, allowing for injection molding and broad applicability, including rapid hemostasis in vivo. Overall, the data robustly support the authors' claims. I recommend addressing the following issues and suggest minor revisions before considering the manuscript for publication.
Double-Network Hydrogel Clarification: The manuscript describes a complex system involving multiple polymers and dynamic bonds, specifically poly(vinyl alcohol) (PVA)/sodium alginate functionalized with dopamine (SA-DOPA) and poly(acrylic acid) (PAA), dynamically crosslinked by borate and incorporating tannic acid (TA). Could the authors clarify why they categorize PVA-SA-DOPA as the first polymer network and PAA as the second? Would it not be more accurate to describe this as a multi-network structure?
Rationale for PAA Addition: What is the reasoning behind the inclusion of PAA as the "second polymer network"? Additionally, could the authors address any potential side effects associated with the high -COOH content of PAA in the context of hemostatic performance?
Component Selection and Composition: Given the hydrogel's complexity, how were the components and their concentrations determined? A more detailed explanation of the formulation process would enhance the manuscript's clarity. Catechol Oxidation Conditions: The authors mention that catechol can easily oxidize to quinone structures. Are there specific conditions or limitations in this study that address this concern? Furthermore, what is the stability of the quinone formed in this context?
Notch Sensitivity Mechanism: The hydrogel demonstrates excellent notch insensitivity. Could the authors elaborate on the underlying principles or mechanisms contributing to this property?

Reviewer #2

(Remarks to the Author)
In this manuscript, Seo and co-workers present a novel double-network, STAT, and its application as a hemostatic agent. In this article, the authors synthesis, mechanics, biocompatibility, and hemostatic potential of STAT through a series of well-designed in vitro and in vivo experiments. The STAT hydrogel has a young's modulus that falls within the range of soft tissue, antibacterial properties, and demonstrates a degree of biocompatibility. The efficacy of STAT is shown through several in vivo hemorrhagic and wound healing models. The findings are significant; however some several comments need to be addressed.

1. Platelets play an integral role in hemostasis and the clotting cascade. The interactions of platelets with STAT were briefly assessed through SEM imaging. Further in vitro assessment of platelet attachment and activation is needed to begin to further understand the impact of STAT on hemostasis.
2. The authors note the young's modulus of STAT is within the range of healthy soft tissue. Additionally, in Fig 6. the tensile strength of healed dermal tissue with STAT application is measured. However, no comparison to un-injured native dermal tissue has been done.
3. More detail is needed for the dermal injures that were conducted. From the representative images taken in Fig 6. it seems like STAST was removed or fell off the dermal defect at day 1. Was this intentional, was the STAT removed after initial

hemostasis, or did the STAT fall off on Day 1.
4. The in vitro transwell experiments were lacking detail. Were the cells in direct contact with the STAT or were these cells incubated in a solution where the STAT was in the upper transwell chamber? The authors are ambiguous in regard to how fibroblast aspect ratio is measured. For a n=3, were only 3 cells measured or multiple cells over 3 wells?
5. For the liver puncture injuries, STAT was applied to the wound and left on the liver for 7 days. Was any histological analysis of the liver puncture done to assess fibrin accumulation or wound healing?
6. The authors do several tests demonstrating how the catechol state allows for easy removal of STAT from skin. At a wound site, the platelet plug and provisional fibrin matrix are as strong as uninjured dermis, were any experiments done where after a traumatic bleed and clotting the STAT was removed? Would STAT removal after clotting disturb the provisional healing matrix.

Reviewer #3

(Remarks to the Author)
I co-reviewed this manuscript with one of the reviewers who provided the listed reports. This is part of the Nature Communications initiative to facilitate training in peer review and to provide appropriate recognition for Early Career Researchers who co-review manuscripts.

Reviewer #4

(Remarks to the Author)
Referees' Comments to the Authors
1. Executive Summary
The authors propose a "STAT" hydrogel utilizing a double-network of PVA/Alginate-Dopamine/Tannic Acid/Borax/PAA. The central claim is the achievement of "instant" wet adhesion via the partial oxidation of catechol to o-quinone, triggered by "brief gloved kneading". While the in vivo hemostatic data in rabbit models are extensive, the study suffers from a fundamental lack of conceptual novelty and scientific rigor. The manuscript is recommended for rejection because the core adhesive mechanism—controlling the oxidation state of catechol—has been extensively reported in the literature with far greater precision. The proposed "kneading" strategy offers no distinct advantage over existing chemical or enzymatic triggers and, in fact, introduces significant uncontrollability.
2. Primary Ground for Rejection: Lack of Novelty & Methodological Regression
The concept of "Oxidation-Triggered Adhesion" is not novel. The authors frame the conversion of catechol to o-quinone as a novel strategy for "instant" adhesion. However, the modulation of the catechol-to-quinone transition to enhance interfacial bonding (via Schiff base or Michael addition) is a foundational concept in mussel-inspired chemistry that has been investigated for over a decade.
• Existing Literature: Numerous prior studies have demonstrated that controlled oxidation (using precise triggers such as $NaIO_4$, enzymatic oxidation by tyrosinase, or pH switches) can dramatically enhance wet adhesion.
• Regression, Not Advancement: The authors' approach of using "brief gloved kneading" in ambient air to induce this oxidation is, scientifically speaking, a regression. Unlike established methods where the degree of oxidation is precisely tuned to balance cohesion (self-crosslinking) and adhesion (interfacial bonding), "kneading" is a crude, uncontrolled, and qualitative manual process.
Verdict: This work does not present a conceptual leap. It essentially replicates a well-known chemical mechanism using a less precise, manual method. It represents an incremental formulation study rather than the fundamental advance required for Nature Communications.
3. Major Scientific Flaws (Critical Issues)
1. The "Black Box" of Kneading-Induced Activation. The reliance on "kneading" undermines the reproducibility of the study.
• Uncontrolled Variables: The oxidation rate of catechol is strictly governed by oxygen availability, pH, and radical generation. Manual kneading introduces uncontrollable variables (kneading intensity, duration, glove material, ambient humidity).
• Lack of Kinetic Control: The authors fail to provide any kinetic data correlating "kneading energy/time" with the precise concentration of quinone groups. Without this, the synthesis is "art," not science.
2. Speculative Mechanism of "Covalent" Bonding The authors attribute the adhesion to covalent bonding (Michael addition) but provide no direct spectroscopic evidence of these bonds at the tissue interface.
• Insufficient Proof: Figure 2 only characterizes the bulk hydrogel (UV-vis, FTIR). The macroscopic adhesion tests (Lap shear, Peel test) cannot distinguish between covalent bonding and strong physical interactions (hydrogen bonding/cation-n) provided by the Tannic Acid and PVA networks.
• Requirement: To claim a "covalent" mechanism, surface-specific analysis (e.g., interfacial XPS or using model amine surfaces) is mandatory.
3. Inappropriate Controls in Preclinical Models The translational value is overstated due to weak experimental design in the large animal models.
• Weak Comparison: In the severe rabbit hemorrhage models (spleen/liver), the STAT hydrogel is compared only against surgical gauze. Gauze is a passive material known to fail in high-flow bleeding.
• Missing Gold Standard: A manuscript of this caliber must compare the proposed material against active clinical standards (e.g., Fibrin Glue, TachoSil). The fact that Fibrin Glue was used in the mild skin model but excluded from the severe bleeding model suggests a selection bias that inflates the perceived efficacy of the hydrogel.
4. Conclusion
While the multicomponent formulation (DN network) yields tough mechanics, the study's core novelty relies on a mechanism (catechol oxidation) that is already well-understood and is applied here in a scientifically uncontrolled manner ("kneading").

Coupled with the lack of rigorous chemical proof for the interface and weak in vivo controls, the manuscript does not meet the standards of novelty and rigor for Nature Communications.

Version 1:

Reviewer comments:

Reviewer #1

(Remarks to the Author)
This work presents a double-network hydrogel hemostatic adhesive fabricated by incorporating borax and catechol-containing components (tannic acid and dopamine) to facilitate crosslinking, enabling rapid adhesion and hemostasis under mild conditions. The authors systematically characterize the hydrogel's crossli nking structure, mechanical properties, biocompatibility, hemostatic performance, and adhesive capabilities. Overall, this is a well-organized study with substantial experimental data. I recommend acceptance after minor revisions.

Following are points for the authors to consider:
1. Two catechol-containing components—tannic acid and dopamine—are employed in this system. What was the rationale behind using both? Do they serve distinct and complementary roles in the hydrogel formulation?
2. The manuscript lacks specific details regarding the composition of the STAT hydrogel. What is the proportion of SA-DOPA in the formulation? How was this particular composition determined or optimized?
3. The authors attribute the wet adhesion mechanism to the oxidation of catechol to ortho-quinone under ambient conditions, which supposedly enables adhesion without external activation. How was it confirmed that oxidation proceeds specifically to ortho-quinone rather than, for instance, dopamine polymerizing into polydopamine? The UV-vis data presented may not be sufficient to draw this conclusion; additional evidence would strengthen the mechanistic interpretation.
4. In Figure 3a, how was the PVA hydrogel prepared for comparison? While it is understandable that incorporating SA-DOPA increases crosslinking and enhances toughness, conventional PVA hydrogels can also exhibit relatively high mechanical strength. A more detailed discussion or benchmarking would be helpful.
5. To better highlight the advancements of this work, it would be beneficial to include a comparative analysis with other hemostatic adhesive hydrogels reported in the literature, particularly in terms of adhesion strength, hemostatic efficacy, and mechanical properties.
6. Were the hydrogels used for performance testing fabricated via 3D printing or conventional mixing/casting methods? If 3D-printed samples were used, do they exhibit comparable performance to their cast counterparts? Clarifying this point would be important given the emphasis on printability.

Reviewer #2

(Remarks to the Author)
The authors have done a nice job of responding to prior comments.

Reviewer #3

(Remarks to the Author)
I co-reviewed this manuscript with one of the reviewers who provided the listed reports. This is part of the Nature Communications initiative to facilitate training in peer review and to provide appropriate recognition for Early Career Researchers who co-review manuscripts.

Reviewer #4

(Remarks to the Author)
The revised work can be accepted in its current form.

Version 2:

Reviewer comments:

Reviewer #1

(Remarks to the Author)
I am satisfied with the revisions; all concerns have been fully addressed. The added data and clarifications have greatly improved the manuscript. Therefore, I recommend acceptance in its current form.

Open Access This Peer Review File is licensed under a Creative Commons Attribution 4.0 International License, which permits use, sharing, adaptation, distribution and reproduction in any medium or format, as long as you give appropriate credit to the original author(s) and the source, provide a link to the Creative Commons license, and indicate if changes were made.
In cases where reviewers are anonymous, credit should be given to 'Anonymous Referee' and the source.
The images or other third party material in this Peer Review File are included in the article's Creative Commons license, unless indicated otherwise in a credit line to the material. If material is not included in the article's Creative Commons license and your intended use is not permitted by statutory regulation or exceeds the permitted use, you will need to obtain permission directly from the copyright holder.
To view a copy of this license, visit https://creativecommons.org/licenses/by/4.0/

Dear Editors and Referees,

Thank you for your interest and thoughtful comments on our paper, "Quinone-Mediated, Tissue-Adaptive Double-Network Hydrogel for Hemostasis and Wet-Tissue Adhesion", submitted for publication in *Nature Communications.* Please find our explanations and answers below, which hopefully address the issues presented, and they are revised in our newly submitted version of the manuscript. The changes made in the manuscript are highlighted in yellow so that they can be easily traced and will be organized separately as an attachment file. *Characters in red refer to the comments made by reviewers.

Reviewer #1:

The manuscript presents a novel high-adhesion hydrogel designed for rapid hemostasis, demonstrating tissue-compatible modulus, excellent adhesion, high strength, and outstanding biocompatibility and antibacterial properties. This hydrogel also exhibits shear-thinning behavior, allowing for injection molding and broad applicability, including rapid hemostasis in vivo. Overall, the data robustly support the authors' claims. I recommend addressing the following issues and suggest minor revisions before considering the manuscript for publication:

Response: We sincerely thank the reviewer for the positive and constructive evaluation of our manuscript. We appreciate the recognition of the novelty and translational potential of our high-adhesion hydrogel system, including its tissue-compatible mechanical properties, strong adhesion performance, injectability enabled by shear-thinning behavior, as well as its excellent biocompatibility and antibacterial activity. We are encouraged by the reviewer's assessment that the experimental data robustly supports our claims. In response to the minor issues raised, we have carefully revised the manuscript accordingly and have addressed all points in detail in the following responses.

Comment 1. Double-Network Hydrogel Clarification: The manuscript describes a complex system involving multiple polymers and dynamic bonds, specifically poly(vinyl alcohol) (PVA)/sodium alginate functionalized with dopamine (SA-DOPA) and poly(acrylic acid) (PAA), dynamically crosslinked by borate and incorporating tannic acid (TA). Could the authors clarify why they categorize PVA-SA-DOPA as the first polymer network and PAA as the second? Would it not be more accurate to describe this as a multi-network structure?

Response: We thank the reviewer for this insightful comment regarding the classification of our hydrogel as a double-network (DN) system. Although the STAT hydrogel contains multiple polymers and dynamic interactions (PVA, SA-DOPA, TA, and borate bonds), we categorized it as a DN hydrogel based on the network formation sequence and structural hierarchy, consistent with the conventional definition of DN hydrogels. Specifically, the first (primary) network is formed

by the freeze-thaw-crosslinked PVA backbone, which is further reinforced by semi-interpenetration of SA-DOPA and dynamic hydrogen bonding/borate ester crosslinking introduced by TA and borax. These components collectively constitute a single, continuous primary framework rather than an independent secondary network. In contrast, the second network is generated only after infusion and *in situ* polymerization of PAA within the pre-formed primary scaffold, producing an interpenetrating and ductile network that provides energy dissipation and mechanical toughness. This design follows the classical DN architecture in which a rigid first network is combined with a softer, extensible secondary network, as widely reported in DN hydrogel literature (e.g., *Soft Matter*.,2014,10(5), 672-687, *Adv. Sci*., 2023, 10, 2303651). To avoid confusion, we have clarified this distinction in the revised manuscript by emphasizing that the STAT hydrogel represents a reinforced DN hydrogel, where multiple dynamic bonds exist within the primary network, while the secondary network is uniquely attributed to the polymerized PAA phase.

Page 9 (Manuscript)

These borate ester linkages are reversible under physiological conditions, contributing to repeated self-healing behavior.[64] In the third step, PAA was infused and polymerized within the primary network (PVA/SA-DOPA/TA/Borax) to generate the secondary network, completing the DN architecture (PVA/SA-DOPA/TA/Borax/PAA: STAT hydrogel network). Although multiple dynamic interactions are incorporated within the primary scaffold (e.g., TA-mediated hydrogen bonding and reversible borate ester linkages), only PAA forms an independently polymerized secondary interpenetrating network; therefore, the STAT system is best categorized as a reinforced double-network hydrogel rather than a multi-network hydrogel, consistent with the established definition of DN architectures.[55, 65] The incorporation of the PAA network endowed the hydrogel with ductile and energy-dissipating properties, allowing large deformation without fracture, which improves overall mechanical toughness. This design synergistically combines a rigid, energy-dissipating network with a ductile, load-bearing network, providing both high strength and extensibility.[66]

Comment 2. Rationale for PAA Addition: What is the reasoning behind the inclusion of PAA as the "second polymer network"? Additionally, could the authors address any potential side effects associated with the high -COOH content of PAA in the context of hemostatic performance?

Response: We thank the reviewer for this important question regarding the rationale for introducing PAA as the second polymer network and the potential concern about its high –COOH content. PAA was incorporated primarily to serve as a ductile secondary interpenetrating network that enhances the overall mechanical toughness, extensibility, and energy dissipation of the hydrogel, rather than to directly contribute to hemostatic activity. The formation of this secondary network enables the hydrogel to maintain structural integrity under large deformation and wet tissue environments, which is critical for practical wound sealing applications. Regarding possible side effects of the carboxyl-rich PAA phase, our experimental results suggest that PAA does not

negatively impact hemostatic performance. As shown in Fig. 4K, blood clotting efficiency is mainly governed by the presence of tannic acid (TA), which provides strong interfacial interactions and promotes rapid coagulation. Notably, the two TA-containing groups (PVA/TA/Borax/SA-DOPA and PVA/TA/Borax/SA-DOPA/PAA) exhibited similarly enhanced clotting performance, indicating that the inclusion of PAA does not alter or impair the hemostatic capability. Therefore, the primary hemostatic functionality originates from TA-mediated interactions, while PAA mainly plays a mechanical reinforcement role as the secondary network component. We have clarified this rationale in the revised manuscript.

Page 20 (Manuscript)

Only the TA-containing hydrogels showed clear clot formation. The blood clotting index (BCI) was calculated to evaluate hemostatic performance (Fig. 4K). Both the PVA/SA-DOPA/TA/Borax and STAT hydrogels exhibited low BCI values of 56.40 ± 1.5 and 58.40 ± 1.7, respectively. These values were approximately two-fold lower than those of the TA-free controls (PVA and PVA/SA-DOPA), a significant improvement attributed to the procoagulant activity of TA. In addition, an *in vitro* platelet-rich plasma (PRP) interaction assay further showed that STAT significantly promoted platelet association with the hydrogel surface compared with the PVA control (Supplementary Fig. 12D), supporting platelet-involved clot stabilization as a contributing mechanism to the rapid hemostatic performance. Notably, the incorporation of the secondary PAA network did not compromise the coagulation performance, as the TA-containing hydrogels exhibited similarly low BCI values regardless of PAA addition. This confirms that hemostatic activity is primarily governed by TA-mediated interactions, while PAA mainly serves as a mechanical reinforcement network. Collectively, these results demonstrate that the hydrogels possess a unique combination of properties: TA-mediated surface antibacterial activity, excellent cell compatibility, low hemolytic potential, and superior hemostatic capability. This multifaceted functionality makes them promising candidates for wound-healing patches in clinical applications.

Figure 4K

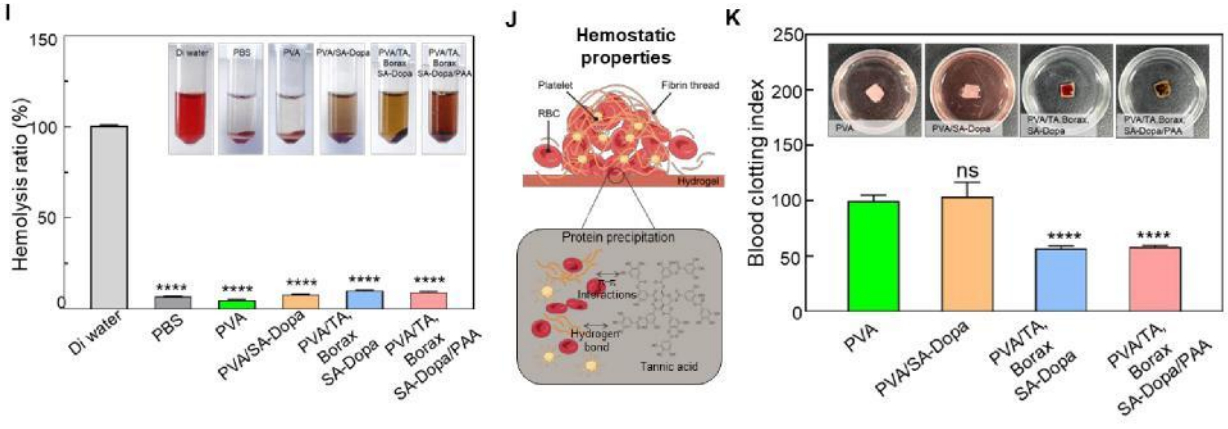

Comment 3. Component Selection and Composition: Given the hydrogel's complexity, how were the components and their concentrations determined? A more detailed explanation of the formulation process would enhance the manuscript's clarity.

Response: We thank the reviewer for this valuable suggestion regarding the formulation rationale and component selection. We agree that a clearer explanation of how the hydrogel composition and concentrations were determined would improve the manuscript. In this study, the concentrations of key functional components were systematically optimized through stepwise experimental screening based on their primary roles in adhesion reinforcement and hemostatic performance. First, the SA-DOPA content was gradually increased to identify the optimal concentration for mechanical strengthening and tissue adhesion. As shown in the newly added Supplementary Fig. 1, the compressive stress increased with SA-DOPA concentration but reached a saturation plateau beyond 0.108 M. Therefore, 0.108 M SA-DOPA was selected for the final formulation, as it provides enhanced interfacial adhesion through catechol-mediated interactions while avoiding unnecessary excess polymer content. Second, the TA concentration was optimized based on its procoagulant contribution to rapid hemostasis. Hemostatic performance was evaluated by the blood clotting index while increasing TA dosage. As presented in Supplementary Fig. 2, clotting efficiency improved markedly with TA addition but exhibited no further significant enhancement beyond 2000 mg, indicating saturation of TA-mediated coagulation effects. Accordingly, 2000 mg TA was chosen as the optimal amount for the STAT hydrogel. Overall, the final formulation was determined by balancing (i) adhesion-promoting SA-DOPA incorporation, (ii) TA-driven hemostatic activity. We have added these optimization results and corresponding explanations to the revised Supplementary Information to improve clarity.

Page 8 (Manuscript)

We used PVA as the backbone polymer. However, PVA hydrogels obtained through the freezing–thawing process are typically brittle and weak, exhibiting poor adhesion to tissue. To enhance tissue adhesion, SA-DOPA was incorporated into the PVA solution. The SA-DOPA concentration was systematically optimized, and 0.108 M was selected as the minimal level achieving saturated mechanical reinforcement (Supplementary Fig. 1). The flexible alginate chains physically entangle with the PVA network, and the catechol groups of DOPA form multiple hydrogen bonds with hydroxyl groups of PVA.

Page 9 (Manuscript)

The catechol groups of SA-DOPA are capable of not only extensive hydrogen bonding with neighboring molecules but also chemical interactions with amine (-NH2) and carboxyl (-COOH) groups, thereby enhancing adhesion to biological substrates.[63] In the second step, TA and borax were introduced to reinforce the network. TA content was also optimized based on hemostatic performance, and 2000 mg was chosen since clotting enhancement saturated beyond this level (Supplementary Fig. 2). TA contains 25 phenolic hydroxyl groups per molecule. These groups are responsible for establishing multiple hydrogen bonds that crosslink the PVA–PVA, PVA–SA-

DOPA, and SA-DOPA–SA-DOPA chains (Fig. 2B, red square box). This dense and reversible network of hydrogen bonds enables rapid reformation upon external damage, thereby imparting self-healing ability to the hydrogel.

Page 2 (Supporting Information, Fig. S1)

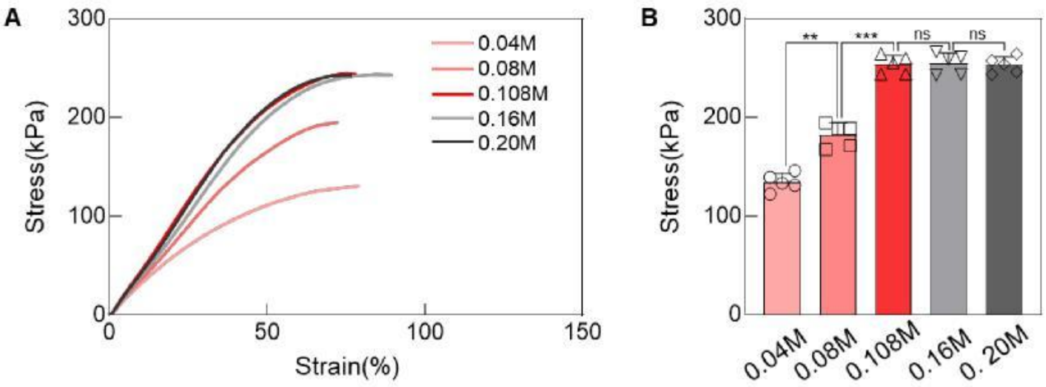

Fig. S1. Optimization of SA-DOPA concentration for STAT hydrogel formation. (A) Representative compressive stress-strain curves of hydrogels prepared with increasing SA-DOPA concentrations. (B) Comparison of maximum compressive stress as a function of SA-DOPA content, showing saturation beyond 0.108 M (n=5). *(*P < 0.05, **P < 0.01, ***P < 0.001, and ****P < 0.0001). ns, not significant.*

Page 3 (Supporting Information, Fig. S2)

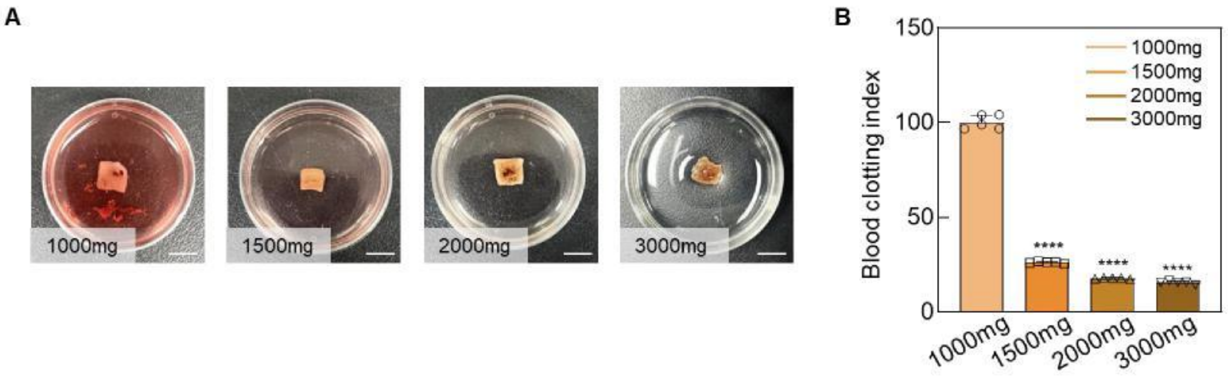

Fig. S2. Optimization of TA concentration for hemostatic performance. (A) Representative photographs of blood coagulation behavior on hydrogels containing increasing TA amounts. (B) Blood clotting index (BCI) as a function of TA dosage, showing saturated coagulation enhancement beyond 2000 mg (n=5). *(*P < 0.05, **P < 0.01, ***P < 0.001, and ****P < 0.0001). ns, not significant.*

Comment 4. Catechol Oxidation Conditions: The authors mention that catechol can easily oxidize to quinone structures. Are there specific conditions or limitations in this study that address this concern? Furthermore, what is the stability of the quinone formed in this context?

Response: We thank the reviewer for this important question regarding catechol oxidation and quinone stability. In the STAT hydrogel, catechol oxidation is not spontaneous or uncontrolled but instead occurs within a well-defined and tunable processing window. Specifically, borax reversibly complexes with catechol groups through borate-diol coordination, reducing the reactivity of free catechols and thereby suppressing premature oxidation during gel fabrication and storage (e.g., Chem. Mater. 2016, 28, 15, 5432–5439, ACS Appl. Mater. Interfaces 2023, 15, 30, 36922–36935). Oxidation is intentionally triggered only upon brief kneading under ambient conditions, which exposes a limited fraction of catechol sites to molecular oxygen as borate complexes partially dissociate (e.g., *ACS Omega* 2021, 6, 8, 5113–5118, *Angew Chem Int Ed Engl.* 2019 Jan 14;58(3):696-714). Under these controlled conditions, only partial catechol-to-quinone conversion occurs (≈15 min), generating sufficient interfacial reactivity for rapid wet-tissue bonding while avoiding excessive bulk over-oxidation. This balance is consistent with widely reported catechol-based adhesive systems, where maintaining a substantial catechol population is critical for durable wet adhesion (e.g.,*Chem. Soc. Rev.*, 2020,49, 433-464). Regarding stability, the quinone species formed in STAT is transient and primarily functions as an interfacial reactive intermediate. Once generated, quinones are rapidly consumed through coupling reactions with tissue nucleophiles or reconverted through dynamic catechol-borate interactions, thereby preventing uncontrolled propagation of oxidation within the bulk network. To further support this mechanism, we performed an additional UV-vis experiment comparing SA-DOPA samples prepared (i) without borax, (ii) with borax under static conditions, and (iii) with borax under continuous mixing at 37 °C. The borax-free group exhibited rapid oxidation, whereas the borax-containing static group showed suppressed oxidation. In contrast, the borax-containing group under mixing conditions displayed promoted catechol-to-quinone conversion, confirming that borax mitigates premature oxidation while enhanced oxygen exposure through mixing or kneading induces controlled partial oxidation (Supplementary Fig. 3). The generated quinone species therefore act primarily as transient intermediates that undergo covalent coupling with nucleophilic groups (amines/thiols) on tissue surfaces, rather than accumulating as unstable reactive species. Finally, after kneading, the oxidation level reached a plateau: when samples were transferred into oxygen-limited (N2-purged) vials, no further darkening was observed during room-temperature storage (Supplementary Fig. 10), confirming that the partially oxidized state remains stable under our experimental conditions.

Page 9 (Manuscript)

This oxidation of catechol to o-quinone is promoted under the mildly basic environment generated by borax and further assisted by dissolved oxygen during the kneading process. Notably, borax can reversibly coordinate with catechol moieties through borate-diol complexation, which helps suppress premature oxidation during static fabrication or storage. Upon mechanical kneading, however, increased exposure to ambient oxygen and partial decomplexation of catechol sites trigger controlled catechol-to-quinone conversion, yielding sufficient interfacial reactivity for rapid wet-tissue bonding. This mechanism was further supported by additional UV-vis analysis showing that borax-containing samples remained largely unoxidized under static conditions,

whereas partial quinone formation was observed only when borax-containing systems were subjected to shearing/mixing (Supplementary Fig. 3).The resulting o-quinone then forms covalent bonds with amine or thiol groups on the tissue surface, which significantly increases adhesive strength. FT-IR spectra confirmed the sequential network formation of the STAT hydrogel.

Page 14 (Manuscript)

In addition, to further confirm that catechol oxidation can be controlled not only during fabrication but also during storage, we examined the effect of limiting oxygen exposure. STAT hydrogels were stored in sealed containers under an N2-purged atmosphere (Supplementary Fig. 10A). After one week, hydrogels stored under ambient conditions exhibited pronounced color darkening, whereas N2-protected samples showed markedly reduced discoloration, indicating attenuated oxidative progression (Supplementary Fig. 10B). Quantitative image analysis confirmed significantly smaller changes in normalized brightness under oxygen-limited storage (Supplementary Fig. 10C). Importantly, STAT hydrogels preserved under N2 maintained their mechanical and adhesive performance after one week, supporting that oxidation reaches a plateau and can be effectively minimized under controlled storage conditions (Supplementary Fig. 10D).

Page 41 (Manuscript)

Borax-Mediated Control of Catechol Oxidation

To validate borax-mediated control of catechol oxidation, SA-DOPA solutions (1 mg mL^{-1} in PBS) were incubated at 37 °C under three conditions: (i) without borax (static), (ii) with borax (5 mM, static), and (iii) with borax (5 mM) under continuous mixing (800 rpm). After incubation for 15 min in ambient air, UV–vis spectra (230–500 nm) were recorded. Quinone formation was quantified by extracting the characteristic absorbance values at ~280 nm (catechol) and ~400 nm (o-quinone band), and by calculating the A400/A280 ratio.

Page 4 (Supporting Information, Fig. S3)

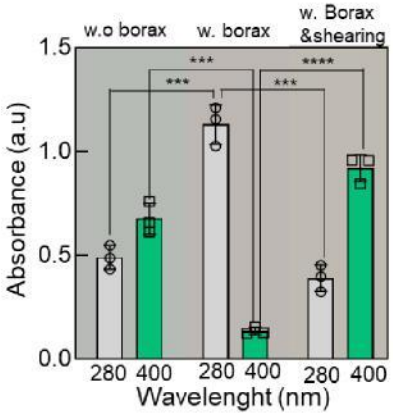

Fig. S3. UV–vis analysis of catechol oxidation in SA-DOPA hydrogels prepared with or without borax (n=3).

Page 11 (Supporting Information, Fig. S10)

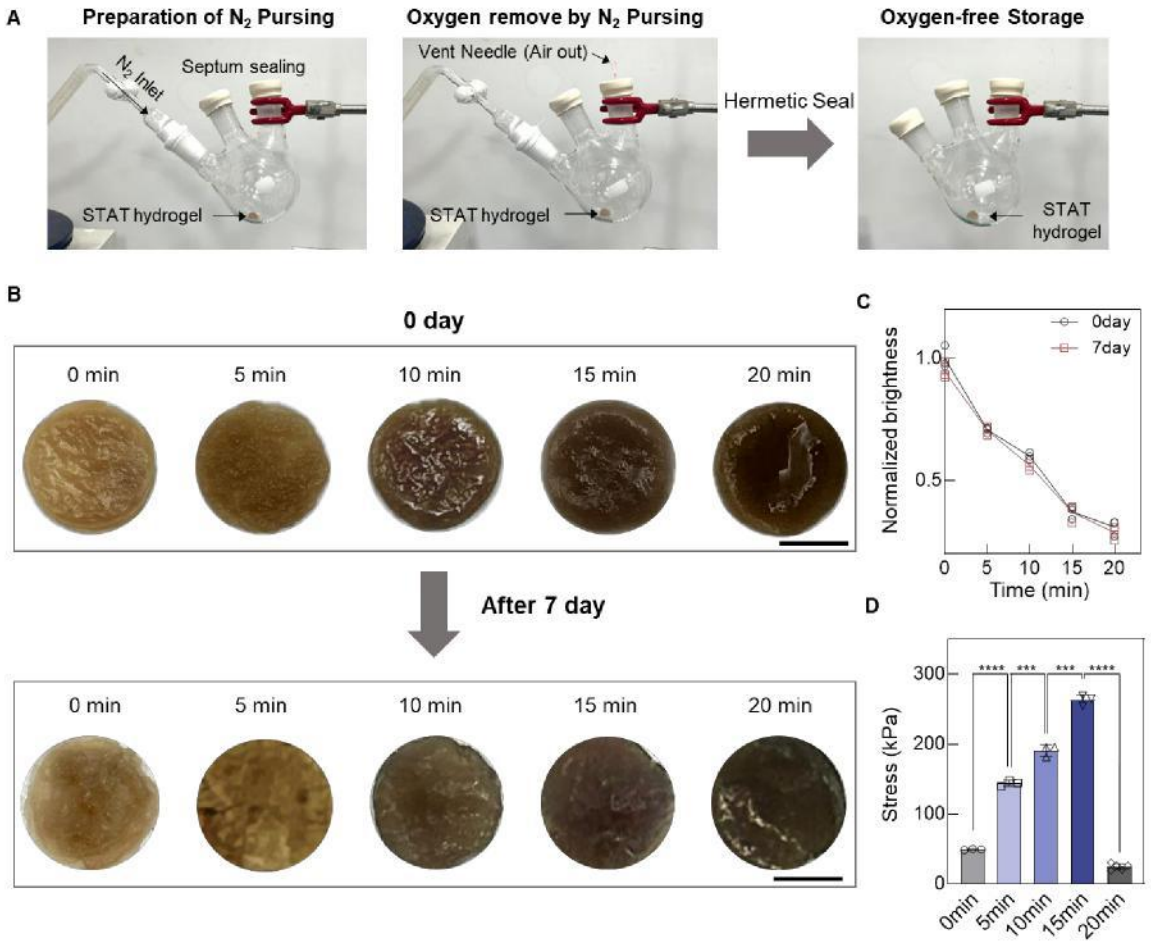

Fig. S10. Suppression of hydrogel oxidation under N2 storage. (A) Photographs showing N2 purging and sealed storage of STAT hydrogels to limit oxygen exposure. (B) Representative images of STAT hydrogels after 1 week of storage under different conditions, revealing reduced color darkening under N2 atmosphere. (C) Quantification of hydrogel color intensity after storage, confirming attenuated oxidation with N2 protection (n=3). (D) Mechanical/adhesion performance measured after 1 week of storage, demonstrating preservation of hydrogel properties under oxygen-limited conditions (n=3). (*P < 0.05, **P < 0.01, ***P < 0.001, and ****P < 0.0001). ns, not significant.

Comment 5. Notch Sensitivity Mechanism: The hydrogel demonstrates excellent notch insensitivity. Could the authors elaborate on the underlying principles or mechanisms contributing to this property?

Response: We thank the reviewer for this insightful question regarding the mechanism underlying the excellent notch insensitivity of the STAT hydrogel. Notch-insensitive behavior in STAT originates from its reinforced double-network structure combined with abundant dynamic sacrificial interactions. As shown in Fig. 3E, F and Supplementary Fig. 11, the STAT hydrogel

retained large elongation (> 600%) even with a notch length of 20% of the sample width, whereas conventional hydrogels typically undergo rapid crack propagation and premature fracture. Mechanistically, high notch tolerance is mainly attributed to two synergistic features. First, the TA-mediated hydrogen-bond network and reversible borate ester linkages serve as sacrificial bonds that break and reform under deformation, effectively dissipating stress concentration at the crack tip. Second, the ductile PAA secondary network maintains structural continuity and provides crack-bridging capability, suppressing sharp crack growth and promoting crack blunting rather than catastrophic propagation. To further support this mechanism, we additionally performed the same notch tensile test on a single-network PVA hydrogel control. In contrast to STAT, the pristine PVA hydrogel exhibited markedly reduced stretchability and fractured readily upon notching (Supplementary Fig. 12), highlighting the critical role of the multi-component dynamic network architecture in achieving notch insensitivity. These results collectively demonstrate that the combination of energy-dissipating sacrificial bonds and a ductile interpenetrating secondary network enables efficient stress delocalization and crack-arresting behavior in STAT hydrogels.

Page 16 (Manuscript)

The PVA single network and SA-DOPA-modified network maintained higher initial stress retention but exhibited gradual decay over time due to insufficient dissipative components. Networks containing TA and borax showed progressive stress relaxation under sustained load due to partial dissociation of hydrogen and borate ester bonds. The STAT hydrogel demonstrated rapid stress relaxation, with stress levels decreasing by half within a few seconds, indicating active network rearrangement via reversible bonding. Notch-insensitive behavior was assessed through tensile tests on samples with varying notch lengths (Fig. 3E, F and Supplementary Fig. 11). Whereas conventional hydrogels fracture readily when notched, our hydrogel maintained elongation beyond 600% even when notched to 20% of its width. This is attributed to the sacrificial bonds from TA and borax that effectively dissipate stress, while PAA chains preserve structural continuity and suppress crack propagation. During tensile deformation, crack blunting rather than sharp propagation was observed. To further validate that this notch tolerance arises from the reinforced dynamic DN architecture, we additionally performed the same notch tensile test using a pristine single-network PVA hydrogel as a control (Supplementary Fig. 12A-C). In contrast to STAT, the PVA hydrogel exhibited pronounced notch sensitivity, with rapid crack initiation and premature fracture upon the introduction of a notch. This comparison highlights that the brittle nature of physically crosslinked PVA alone is insufficient to delocalize stress concentration at the crack tip, whereas the incorporation of dynamic sacrificial interactions (TA/borax) together with the ductile PAA secondary network enables efficient energy dissipation and crack-arresting behavior. Self-healing performance was evaluated by rejoining cut hydrogel specimens and monitoring recovery of mechanical properties over time. As shown in Figure 3G, cut samples adhered within seconds and could be stretched up to 479% after healing.

Page 13 (Supporting Information, Fig. S12)

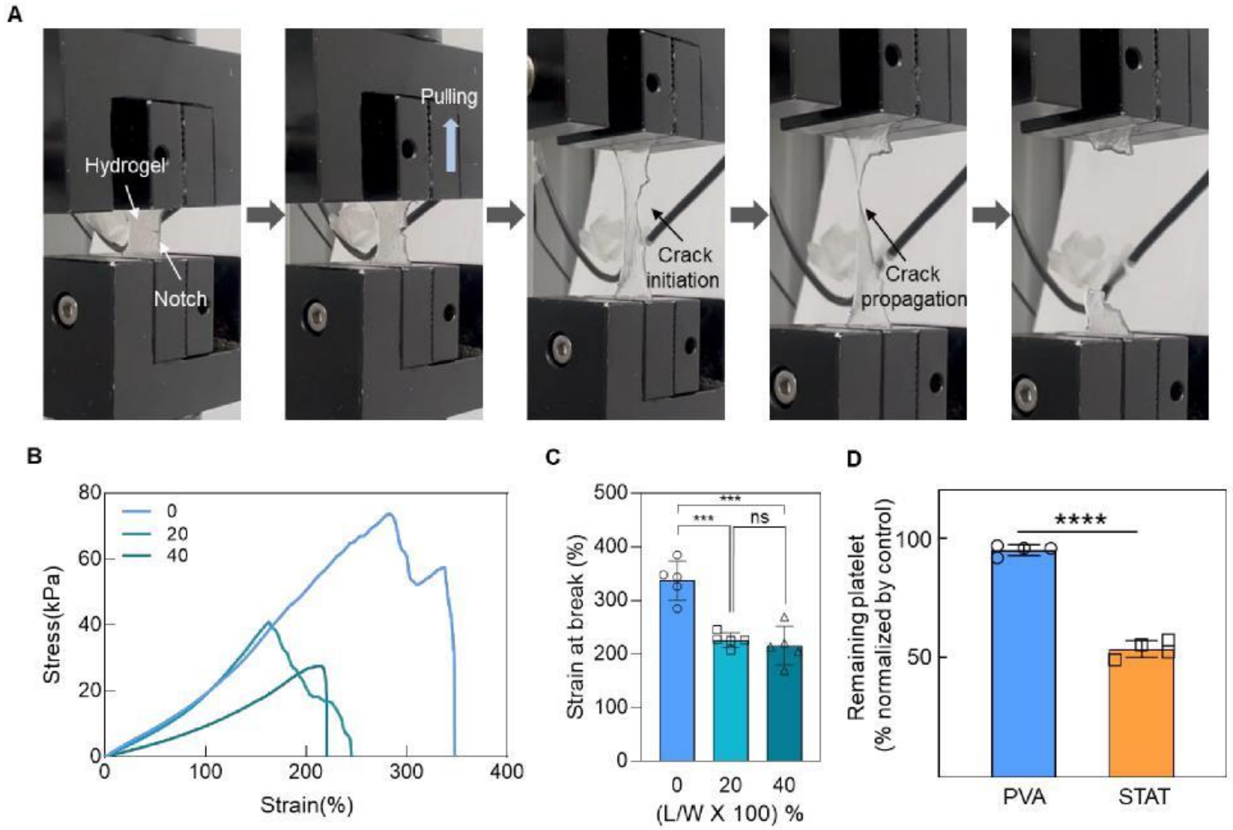

Fig. S12. Notch tolerance and platelet association of STAT hydrogels. (A)Sequential image showing elongation of the notch-introduced PVA hydrogel. (B) Strain at break values of the PVA hydrogel with varying notch lengths. (C) Statistical analysis of the strain-at-break of the notch-introduced PVA hydrogel (n=5). (D) Remaining platelets in PRP supernatant after 5 min contact with PVA or STAT hydrogels. STAT exhibited significantly enhanced platelet association compared with PVA. *(*P < 0.05, **P < 0.01, ***P < 0.001, and ****P < 0.0001). ns, not significant.*

Reviewer #2:

In this manuscript, Seo and co-workers present a novel double-network, STAT, and its application as a hemostatic agent. In this article, the authors synthesis, mechanics, biocompatibility, and hemostatic potential of STAT through a series of well-designed *in vitro* and *in vivo* experiments. The STAT hydrogel has a young's modulus that falls within the range of soft tissue, antibacterial properties, and demonstrates a degree of biocompatibility. The efficacy of STAT is shown through several *in vivo* hemorrhagic and wound healing models. The findings are significant; however some several comments need to be addressed.

Response: We sincerely thank the reviewer for the thoughtful and constructive evaluation of our manuscript. We greatly appreciate the reviewer's recognition of the novelty and translational significance of the STAT double-network hydrogel. We are encouraged that the reviewer finds our *in vitro* and *in vivo* experimental design well-organized and that the overall findings are considered significant. In response to the reviewer's valuable comments and suggestions, we have carefully revised the manuscript to address all concerns in detail. All revisions have been incorporated into the updated version, and point-by-point responses are provided below.

Comment 1. Platelets play an integral role in hemostasis and the clotting cascade. The interactions of platelets with STAT were briefly assessed through SEM imaging. Further *in vitro* assessment of platelet attachment and activation is needed to begin to further understand the impact of STAT on hemostasis.

Response: We thank the reviewer for this important suggestion regarding platelet interactions with STAT. We agree that platelets play a central role in hemostasis, and that additional *in vitro* assessment beyond qualitative SEM images would further strengthen the mechanistic understanding of STAT-induced coagulation. In response, we have performed an additional *in vitro* platelet-rich plasma (PRP) adhesion study to quantitatively evaluate platelet attachment on STAT compared with control hydrogels. PRP extracted from mouse blood was incubated on the hydrogel surfaces for a fixed duration, followed by gentle washing and fluorescence-based quantification of platelet adhesion. The results confirm that STAT promotes significantly enhanced platelet attachment relative to non-hemostatic control hydrogels, supporting the platelet-associated clot formation observed in the SEM images (Fig. 5D). These new results have been included in the revised Supplementary Information (Fig. S12D), providing further evidence that STAT facilitates rapid hemostasis through platelet recruitment and surface-mediated clot stabilization.

Page 39 (Manuscript)

The blood clotting index (BCI) was calculated to evaluate hemostatic performance (Fig. 4K). Both the PVA/SA-DOPA/TA/Borax and STAT hydrogels exhibited low BCI values of 56.40 ± 1.5 and 58.40 ± 1.7, respectively. These values were approximately two-fold lower than those of the TA-free controls (PVA and PVA/SA-DOPA), a significant improvement attributed to the procoagulant activity of TA. In addition, an *in vitro* platelet-rich plasma (PRP) interaction assay further showed

that STAT significantly promoted platelet association with the hydrogel surface compared with the PVA control (Supplementary Fig. 12D), supporting platelet-involved clot stabilization as a contributing mechanism to the rapid hemostatic performance. Notably, the incorporation of the secondary PAA network did not compromise the coagulation performance, as the TA-containing hydrogels exhibited similarly low BCI values regardless of PAA addition. This confirms that hemostatic activity is primarily governed by TA-mediated interactions, while PAA mainly serves as a mechanical reinforcement network. Collectively, these results demonstrate that the hydrogels possess a unique combination of properties: TA-mediated surface antibacterial activity, excellent cell compatibility, low hemolytic potential, and superior hemostatic capability.

Page 50 (Manuscript)

In vitro platelet-rich plasma (PRP) interaction assay

Mouse whole blood was collected into tubes preloaded with 3.2% sodium citrate (blood:citrate = 9:1, v/v) and processed immediately at room temperature. To obtain platelet-rich plasma (PRP), anticoagulated blood was centrifuged at 200 × g for 10 min (no brake). The upper plasma layer was carefully aspirated without disturbing the buffy coat and transferred to a new tube as PRP. For all PRP-based assays, PRP was gently mixed by slow inversion prior to use to minimize unintended platelet activation. Square hydrogel specimens (0.5 × 0.5 cm^2) of either PVA hydrogel or STAT were placed in a sterile plate. PRP (50 µL) was gently dropped onto the surface of each hydrogel and incubated under static conditions at 37 °C for 5 min in a humidified chamber. After incubation, the supernatant PRP was carefully recovered without disturbing the hydrogel surface, and the remaining platelet number in the recovered PRP was quantified by manual counting using a hemocytometer. Platelet counts were normalized to a PRP-only control incubated under identical conditions in the absence of hydrogel contact (defined as 100%).

Page 13 (Supporting Information, Fig. S12)

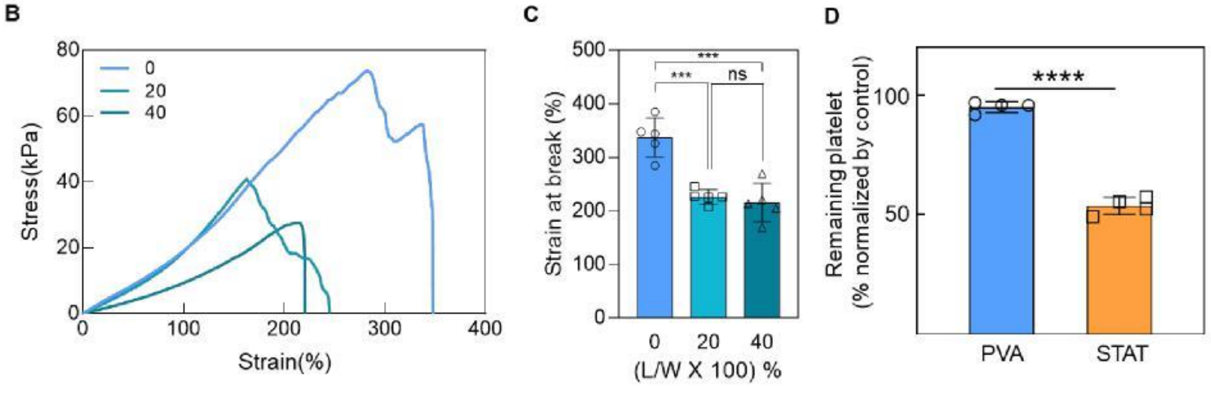

(D) Remaining platelets in PRP supernatant after 5 min contact with PVA or STAT hydrogels. STAT exhibited significantly enhanced platelet association compared with PVA. *(*P < 0.05, **P < 0.01, ***P < 0.001, and ****P < 0.0001). ns, not significant.*

Comment 2. The authors note the young's modulus of STAT is within the range of healthy soft tissue. Additionally, in Fig 6. the tensile strength of healed dermal tissue with STAT application is measured. However, no comparison to un-injured native dermal tissue has been done.

Response:

We thank the reviewer for this valuable suggestion. We agree that comparison with uninjured native dermal tissue provides an important benchmark for interpreting the mechanical recovery of healed skin. In response, we have performed an additional tensile test using uninjured dorsal skin harvested from healthy mice as a native tissue control. As expected, both the suture-treated and STAT-treated healed tissues exhibited lower tensile strength than the uninjured dermis at day 14, reflecting incomplete restoration during the wound remodeling stage. Importantly, however, the STAT-treated group demonstrated markedly improved tensile strength compared with the conventional suture group, showing significantly closer recovery toward the native tissue baseline. Statistical analysis further confirmed a pronounced difference between STAT and the control treatment, highlighting the enhanced wound reinforcement and tissue repair promoted by STAT application. These new results and the corresponding comparison to native dermal tissue have been added to the revised manuscript (Supplementary Fig. 19).

Page 26 (Manuscript)

Quantitative analysis further confirmed that the breaking strength of the STAT hydrogel-treated skin was significantly higher than that of sutured skin, demonstrating that the STAT hydrogel application led to more effective mechanical regeneration of the wound tissue (Fig. 6L). For reference, tensile strength was also compared with uninjured native dorsal skin harvested from healthy mice. As expected, healed tissues at day 14 remained weaker than native dermis due to ongoing remodeling; however, STAT-treated wounds exhibited significantly closer recovery toward the native baseline than the suture control (Supplementary Fig. 19). Collectively, these results demonstrate that the STAT hydrogel holds significant potential as a next-generation material for effective wound closure and accelerated healing in clinical settings.

Page 20 (Supporting Information, Fig. S19)

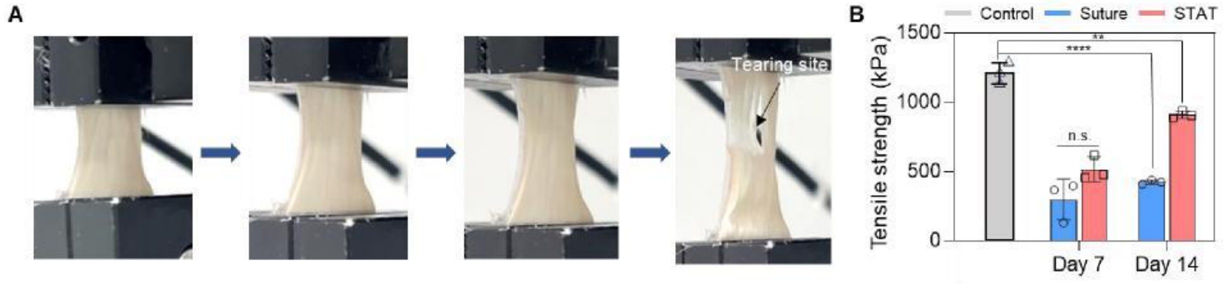

Fig. S19. Tensile strength recovery of healed dermal tissue. (A) Representative photographs of tensile testing of excised dorsal skin samples. (B) Tensile strength comparison of uninjured native dermis and healed wounds treated with suture or STAT at day 14. *(*P < 0.05, **P < 0.01, ***P < 0.001, and ****P < 0.0001). ns, not significant.*

Comment 3. More detail is needed for the dermal injures that were conducted. From the representative images taken in Fig 6. it seems like STAST was removed or fell off the dermal defect at day 1. Was this intentional, was the STAT removed after initial hemostasis, or did the STAT fall off on Day 1.

Response: We thank the reviewer for this important question regarding the dermal wound images in Fig. 6G. We clarify that the apparent absence of STAT on day 1 was not due to accidental detachment or premature falling off. Instead, STAT was intentionally removed after the initial wound-sealing phase in order to obtain representative photographs and to perform subsequent histological evaluation of the underlying regenerated tissue. Specifically, because STAT exhibits strong tissue adhesion through dynamic catechol and borate-assisted interactions, we applied a sorbitol-based triggering solution to partially disrupt reversible borate ester bonding and thereby gently reduce adhesion for controlled detachment. This enabled non-destructive removal of the hydrogel without damaging the healing tissue surface. To further support this controlled removal process, we additionally performed an *ex vivo* porcine skin test, confirming that sorbitol treatment facilitates hydrogel detachment easier compared with untreated conditions (Supplementary Movie 2). Therefore, the day 1 image reflects a planned removal step for analysis rather than material instability or unintended loss *in vivo*.

Page 12 (Manuscript)

Adhesion testing on porcine skin demonstrated that pre-oxidized hydrogels adhered but could be removed easily, whereas oxidized hydrogels exhibited markedly stronger adhesion and resisted detachment (Fig. 2I and Supplementary Movie 1). In addition, because catechol–borate-assisted interactions remain partially reversible, adhesion could be gently reduced when required by applying a sorbitol-based triggering solution, enabling controlled detachment without disrupting the tissue surface (Supplementary Movie 2).

Page 44 (Manuscript)

Sorbitol-Triggered Detachment of Hydrogel

To evaluate the on-demand detachment of the hydrogel from the porcine skin, a 0.1 M solution of D-sorbitol (Sigma-Aldrich, USA) was prepared in PBS. A gauze pad was thoroughly saturated with the 0.1 M D-sorbitol solution and placed directly onto the hydrogel adhered to the porcine skin. After incubating for 3 min at room temperature to allow the sorbitol to diffuse into the hydrogel, the hydrogel was gently detached from the porcine skin surface.

Comment 4. The *in vitro* transwell experiments were lacking detail. Were the cells in direct contact with the STAT or were these cells incubated in a solution where the STAT was in the upper transwell chamber? The authors are ambiguous in regard to how fibroblast aspect ratio is measured. For a n=3, were only 3 cells measured or multiple cells over 3 wells?

Response: We thank the reviewer for pointing out the ambiguity in the description of the transwell assay and morphology quantification. We clarify that NIH-3T3 fibroblasts were not in direct

physical contact with STAT. The hydrogel samples were placed in the upper transwell insert, while cells were cultured in the lower chamber, allowing only diffusible components to interact through the porous membrane. In addition, the aspect ratio analysis was performed by quantifying about 50 cells per well from multiple random fields, with n=3 indicating three independent wells per group rather than three individual cells. These methodological details have now been added to the revised manuscript for clarity.

Page 48 (Manuscript)

The cells were seeded at a concentration of 0.5 × 10^5 cells/mL in 6-well plates, each containing 1.5 mL of high-glucose Dulbecco's Modified Eagle's Medium (DMEM, 11965092, Gibco, USA) supplemented with 10% fetal bovine serum (A5670701, Gibco, USA) and 1% penicillin-streptomycin (15070063, Gibco, USA). The STAT hydrogel was placed in the upper transwell insert, while NIH-3T3 fibroblasts were cultured in the lower chamber, ensuring that cells were not in direct physical contact with the hydrogel but were exposed to soluble factors diffusing through the porous membrane. Two complementary assays were employed to assess cell viability: the Live/Dead assay (L3224, Invitrogen, USA) and the CCK-8 assay (Thermo Scientific, USA). For the Live/Dead assay, cells were stained according to the manufacturer's instructions, and fluorescence images were captured using an inverted fluorescence microscope (IX81, Olympus, Japan) at 10× magnification.

Page 49 (Manuscript)

The relative cell viability was determined and compared between the different time points. To assess cell morphology, the aspect ratio of the cells, defined as the ratio between the major and minor cell axes, was calculated. For this, ~50 cells were quantified per well from three random fields (n=3 independent wells). Fluorescent staining techniques were used to visualize the cytoskeleton and cell nuclei. NIH-3T3 cells were stained with Alexa 594-conjugated phalloidin (Thermo Scientific, USA) to label the actin filaments and DAPI (Thermo Scientific, USA) to stain the cell nuclei. The cells were fixed with 4% paraformaldehyde for 15 m at room temperature, followed by permeabilization with 0.1% Triton X-100 in PBS for 5 m. The cells were then incubated with DAPI solution for 5 m at room temperature in the absence of light.

Comment 5. For the liver puncture injuries, STAT was applied to the wound and left on the liver for 7 days. Was any histological analysis of the liver puncture done to assess fibrin accumulation or wound healing?

Response: We thank the reviewer for this important question. In response, we performed additional histological analysis of the liver puncture site after 7 days of STAT treatment. As shown in the newly added H&E sections (Supplementary Fig. 16), the day 0 injury site was initially sealed by blood clot formation, whereas by day 7 the defect space was markedly reduced and replaced by progressive tissue, indicating ongoing wound closure and tissue remodeling. These results provide histological evidence supporting both acute hemostasis and subsequent tissue repair promoted by

STAT. Due to the severe and uncontrolled bleeding in the gauze-treated group, animals could not be maintained for long-term histological assessment on day 7. Therefore, histological evaluation was focused on the STAT-treated liver tissues.

Page 22 (Manuscript)

Quantitative analysis further supported these observations (Fig. 5C). The STAT hydrogel group exhibited a dramatic reduction in total blood loss compared to both the untreated and gauze groups. Furthermore, the hemostatic time was shortened to just a few seconds. These results highlight the superior hemostatic efficacy of the hydrogel. In addition, histological analysis of the puncture site confirmed initial clot sealing at day 0 and substantial reduction of the defect space with progressive repair tissue formation by day 7 (Supplementary Fig. 16B), suggesting that STAT not only achieves rapid hemostasis but also supports subsequent wound closure in liver tissue. To investigate the hemostatic mechanism, the surfaces of gauze and STAT hydrogel were examined via SEM (Fig. 5D).

Page 17 (Supporting Information, Fig. S16)

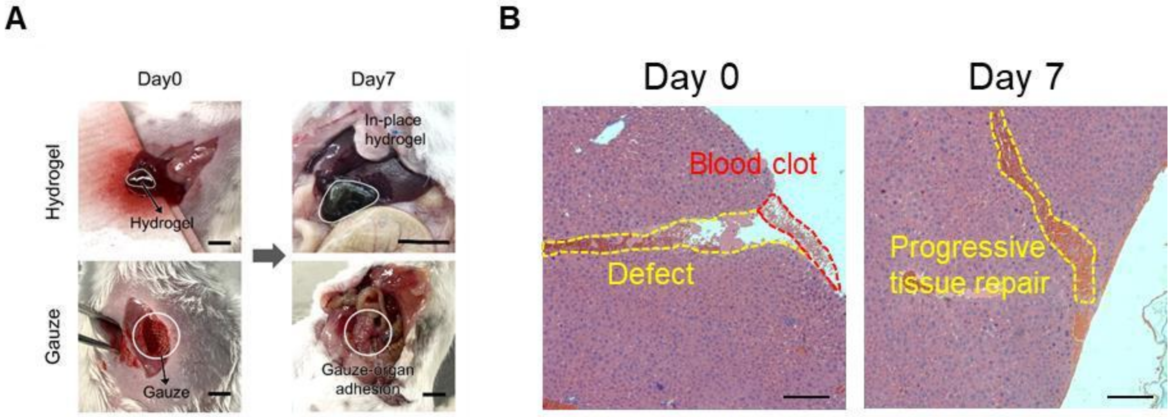

Fig. S16. Photographs showing the STAT hydrogel and the gauze attached to liver tissue. Comparison of the STAT hydrogel and the gauze on the liver bleeding site on Day 0 and Day 7 (scale bar: 5 mm). (B) H&E staining images of the repaired skin after 7 days (scale bar: 100 μm).

Comment 6. The authors do several tests demonstrating how the catechol state allows for easy removal of STAT from skin. At a wound site, the platelet plug and provisional fibrin matrix are as strong as uninjured dermis, were any experiments done where after a traumatic bleed and clotting the STAT was removed? Would STAT removal after clotting disturb the provisional healing matrix.

Response: We thank the reviewer for raising this important question. We would like to clarify that STAT is not intended to be routinely removed immediately after clot formation at actively bleeding traumatic sites. Rather, STAT is designed to function as an adhesive hemostatic patch that remains in place during the acute phase to stabilize the platelet/fibrin plug and seal the wound surface.

Therefore, removal experiments directly after clotting in severe bleeding models (e.g., liver puncture) were not performed, as forced mechanical detachment could risk disturbing the provisional matrix. However, in the dermal wound setting, where removal may be required for imaging or tissue assessment, we performed an additional controlled detachment test using sorbitol treatment to partially weaken reversible borate-assisted interactions. Notably, even in mildly bleeding skin wound conditions after initial clotting, sorbitol-triggered release enabled gentle hydrogel removal without visible disruption of the wound surface or tissue tearing, supporting that STAT can be non-destructively detached when necessary under controlled conditions (Fig 6G and Supplementary Movie 2). We have clarified this intended usage and the role of sorbitol-triggered detachment in the revised manuscript.

Page 12 (Manuscript)

Adhesion testing on porcine skin demonstrated that pre-oxidized hydrogels adhered but could be removed easily, whereas oxidized hydrogels exhibited markedly stronger adhesion and resisted detachment (Fig. 2I and Supplementary Movie 1). In addition, because catechol–borate-assisted interactions remain partially reversible, adhesion could be gently reduced when required by applying a sorbitol-based triggering solution, enabling controlled detachment without disrupting the tissue surface (Supplementary Movie 2).

Page 44 (Manuscript)

Sorbitol-Triggered Detachment of Hydrogel

To evaluate the on-demand detachment of the hydrogel from the porcine skin, a 0.1 M solution of D-sorbitol (Sigma-Aldrich, USA) was prepared in PBS. A gauze pad was thoroughly saturated with the 0.1 M D-sorbitol solution and placed directly onto the hydrogel adhered to the porcine skin. After incubating for 3 min at room temperature to allow the sorbitol to diffuse into the hydrogel, the hydrogel was gently detached from the porcine skin surface.

<u>Reviewer #3:</u>

I co-reviewed this manuscript with one of the reviewers who provided the listed reports. This is part of the Nature Communications initiative to facilitate training in peer review and to provide appropriate recognition for Early Career Researchers who co-review manuscripts.

Response: We thank Reviewer #3 for their time and for participating in the Nature Communications co-reviewing initiative. We appreciate their contribution to the peer-review process and are pleased to have our work reviewed as part of this professional training program.

Reviewer #4:

1. Executive Summary

The authors propose a "STAT" hydrogel utilizing a double-network of PVA/Alginate-Dopamine/Tannic Acid/Borax/PAA. The central claim is the achievement of "instant" wet adhesion via the partial oxidation of catechol to o-quinone, triggered by "brief gloved kneading". While the *in vivo* hemostatic data in rabbit models are extensive, the study suffers from a fundamental lack of conceptual novelty and scientific rigor. The manuscript is recommended for rejection because the core adhesive mechanism—controlling the oxidation state of catechol—has been extensively reported in the literature with far greater precision. The proposed "kneading" strategy offers no distinct advantage over existing chemical or enzymatic triggers and, in fact, introduces significant uncontrollability.

Response: We sincerely thank the reviewer for providing this overall assessment of our manuscript and for raising important concerns regarding conceptual novelty and mechanistic rigor. We fully acknowledge that catechol-to-quinone conversion and mussel-inspired wet adhesion mechanisms have been extensively explored in the literature. However, we would like to clarify that the key contribution of our work does not lie in redefining catechol oxidation chemistry itself, but rather in the practical implementation of a clinically deployable, reagent-free adhesion and hemostasis strategy within a mechanically reinforced double-network hydrogel designed for severe wet bleeding environments. Importantly, STAT is not intended as a reinvention of catechol chemistry; instead, our advance lies in integrating multiple clinically relevant requirements-instant wet adhesion, robust mechanical integrity, notch-insensitive toughness, rapid self-healing, and strong hemostatic efficacy into a single injectable and moldable platform that functions under high-flow hemorrhagic conditions. Unlike many previous catechol-based adhesives that rely on external oxidants, enzymatic triggers, or multi-step activation schemes, STAT enables immediate wet-tissue bonding through an intrinsic and simple activation process that does not require auxiliary reagents. This reagent-free feature is particularly advantageous in emergency or intraoperative bleeding scenarios, where additional chemical triggers are impractical.

We also respectfully disagree that the kneading strategy introduces uncontrollability. STAT does not rely on spontaneous or runaway catechol oxidation during arbitrary handling. Instead, borax plays a central regulatory role by reversibly complexing with catechol groups through borate-diol coordination, thereby suppressing premature oxidation during fabrication and storage and establishing a well-defined activation window (e.g., *Chem. Mater.* 2016, 28, 15, 5432–5439, *ACS Appl. Mater. Interfaces* 2023, 15, 30, 36922–36935).

We note that even under manual handling conditions, the STAT hydrogel exhibited a high degree of reproducibility in its adhesive and hemostatic performance, with minimal batch-to-batch variation observed across independent preparations. This robustness arises from the intrinsic buffering and regulatory role of borax–catechol coordination, which limits uncontrolled oxidation during handling and provides a defined activation window. Nevertheless, to further elevate experimental rigor and to explicitly address potential concerns regarding operator-dependent variability, we subsequently adopted a device-controlled mechanical mixing approach, as

described below. Moreover, we note that kneading-induced activation itself is not an unscientific or unprecedented concept, but rather a mechanically defined stimulus that has been increasingly adopted in recent hydrogel systems to trigger reproducible chemical or structural responses. For example, recent studies have demonstrated that controlled kneading or mechanical manipulation can be used to activate oxidation-dependent crosslinking or to induce on-demand functional responses in hydrogel networks, including dough-kneading-inspired bulk hydrogel fabrication strategies (*Adv. Funct. Mater.* 2025, 35, 2506602) and kneading-responsive hyaluronic acid-based hydrogels for mechanically triggered drug release (*Acta Biomater*. 2026, 210, 160-175). These reports collectively support the notion that kneading can serve as a well-defined mechanical input, rather than an arbitrary or purely empirical handling step.

In response to the reviewer's concern, we have added new mechanistic evidence demonstrating that catechol-to-quinone conversion occurs only under brief and standardized mechanical mixing conditions using a controlled mixing device (Supplementary Fig. 8) (e.g., *ACS Omega* 2021, 6, 8, 5113–5118, *Angew Chem Int Ed Engl.* 2019 Jan 14;58(3):696-714, *Chem. Soc. Rev.*, 2020,49, 433-464). As shown in the newly added UV-vis analysis, borax-containing samples remain largely unoxidized under static conditions. In contrast, partial quinone formation is promoted only upon time-defined shear/mixing at 37 °C, confirming that oxidation is reproducible and controllable rather than stochastic (Supplementary Fig. 3).

Nevertheless, we fully appreciate the reviewer's concern regarding reproducibility and experimental rigor. In line with this feedback, we have further strengthened the manuscript by replacing manual hand-kneading with a controlled mechanical mixing device that applies standardized shear and mixing duration. This device-based approach enables precise regulation of the activation conditions and allows quantitative correlation between mechanical input and catechol-to-quinone conversion. Importantly, the newly added data confirm that oxidation levels are highly reproducible across independent batches when activated under device-defined kneading conditions, thereby eliminating operator-dependent variability and further reinforcing the controllability of the STAT activation process.

Furthermore, we show that oxidation does not continuously progress after activation. Instead, the oxidation level rapidly reaches a plateau, and no further darkening or property drift was observed when samples were transferred into oxygen-limited (N2-purged) storage conditions (Supplementary Fig. 10). This additional data directly address the reviewer's concern by demonstrating that catechol activation in STAT is tunable, reproducible, and intrinsically regulated, rather than uncontrollable. Taken together, while the fundamental chemistry of catechol oxidation is well established, STAT provides a distinct translational advance by coupling controlled partial oxidation with a mechanically reinforced double-network architecture. This integrated design enables instant and durable wet adhesion as well as effective hemostasis in stringent *in vivo* rabbit hemorrhage models. We believe this integrated, clinically relevant implementation represents a meaningful step beyond prior catechol-based adhesive systems that remain limited by brittle mechanics, poor crack tolerance, or reliance on external chemical activation.

Summary of newly added data

1. We included additional UV-vis analysis showing that borax suppresses premature catechol oxidation under static conditions, while catechol-to-quinone conversion is selectively promoted only under mechanical mixing/kneading (Supplementary Fig. 3).
2. To ensure reproducibility beyond manual handling, we performed kneading using a controlled mechanical mixing device at 37 °C, providing standardized activation conditions rather than relying solely on gloved kneading (Supplementary Fig. 8).
3. Time-dependent analyses were conducted under defined mixing durations (0, 5, 10, 15, and 20 min), confirming that quinone formation proceeds in a controllable and saturating manner (Supplementary Fig. 8B-D).
4. We further evaluated the mechanical stability of STAT after kneading and subsequent storage at room temperature, demonstrating that the hydrogel properties (e.g., stress and modulus) remain stable (Supplementary Fig. 9).
5. To evaluate long-term storage stability, under oxygen-limited conditions (N2-purged environment), no further oxidation was observed over time, supporting that the partially oxidized state reaches a plateau rather than progressing uncontrollably (Supplementary Fig. 10).
6. To strengthen clinical benchmarking in severe rabbit hemorrhage models, we performed additional comparative hemostasis experiments against two FDA-approved surgical standards, fibrin glue (liquid-type sealant) and the TachoSil patch (patch-type hemostatic dressing), providing rigorous gold-standard controls beyond gauze alone (Supplementary Fig. 17).

We have revised the manuscript to more clearly articulate that the novelty resides in this mechanically robust, multifunctional DN system with controlled user-triggered catechol activation, rather than in catechol oxidation as an isolated chemical concept.

2. Primary Ground for Rejection:
Lack of Novelty & Methodological Regression
The concept of "Oxidation-Triggered Adhesion" is not novel. The authors frame the conversion of catechol to o-quinone as a novel strategy for "instant" adhesion. However, the modulation of the catechol-to-quinone transition to enhance interfacial bonding (via Schiff base or Michael addition) is a foundational concept in mussel-inspired chemistry that has been investigated for over a decade.
Response: We thank the reviewer for this comment. We fully acknowledge that oxidation-triggered catechol-to-quinone conversion and subsequent Schiff base/Michael addition chemistry are foundational concepts in mussel-inspired adhesion and have been investigated in previously. Accordingly, we do not claim catechol oxidation itself as a new chemical principle. Instead, the novelty of STAT lies in the translational implementation of controlled, reagent-free catechol activation within a multifunctional hemostatic double-network hydrogel platform, rather than in the oxidation mechanism in isolation. STAT integrates a mechanically tough and notch-insensitive

double-network architecture with TA-driven rapid coagulation and antibacterial functionality. In addition, borax provides a suppression-activation mechanism that prevents premature oxidation during fabrication and storage, while enabling rapid on-demand quinone formation immediately before application without external oxidants or enzymatic triggers.

Moreover, STAT was not developed as a minimal catechol adhesive formulation, but as a systematically engineered platform in which key compositional parameters (e.g., TA, SA-DOPA) were quantitatively optimized to achieve saturated coagulation performance and mechanical robustness under severe bleeding conditions (Supplementary Fig. 1, 2). We have revised the manuscript to clarify that the advance is this system-level integration and clinically practical activation strategy for severe bleeding environments, where many existing catechol-based adhesives remain limited by brittle mechanics or reliance on auxiliary chemical activation. We have also moderated the wording of "instant" adhesion accordingly.

Page 8 (Manuscript)

We used PVA as the backbone polymer. However, PVA hydrogels obtained through the freezing–thawing process are typically brittle and weak, exhibiting poor adhesion to tissue. To enhance tissue adhesion, SA-DOPA was incorporated into the PVA solution. The SA-DOPA concentration was systematically optimized, and 0.108 M was selected as the minimal level achieving saturated mechanical reinforcement (Supplementary Fig. 1). The flexible alginate chains physically entangle with the PVA network, and the catechol groups of DOPA form multiple hydrogen bonds with hydroxyl groups of PVA.

Page 9 (Manuscript)

The catechol groups of SA-DOPA are capable of not only extensive hydrogen bonding with neighboring molecules but also chemical interactions with amine (-NH2) and carboxyl (-COOH) groups, thereby enhancing adhesion to biological substrates.[63] In the second step, TA and borax were introduced to reinforce the network. TA content was also optimized based on hemostatic performance, and 2000 mg was chosen since clotting enhancement saturated beyond this level (Supplementary Fig. 2). TA contains 25 phenolic hydroxyl groups per molecule. These groups are responsible for establishing multiple hydrogen bonds that crosslink the PVA–PVA, PVA–SA-DOPA, and SA-DOPA–SA-DOPA chains (Fig. 2B, red square box). This dense and reversible network of hydrogen bonds enables rapid reformation upon external damage, thereby imparting self-healing ability to the hydrogel.

Page 2 (Supporting Information, Fig. S1)

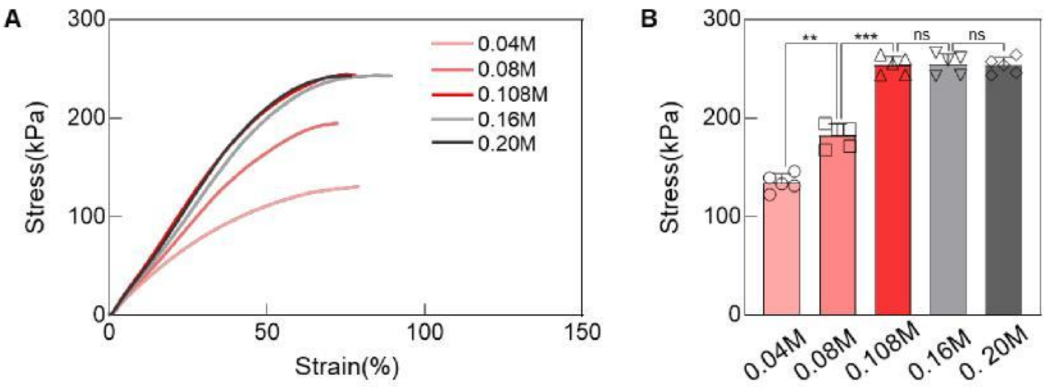

Fig. S1. Optimization of SA-DOPA concentration for STAT hydrogel formation. (A) Representative compressive stress-strain curves of hydrogels prepared with increasing SA-DOPA concentrations. (B) Comparison of maximum compressive stress as a function of SA-DOPA content, showing saturation beyond 0.108 M (n=5). *(*P < 0.05, **P < 0.01, ***P < 0.001, and ****P < 0.0001). ns, not significant.*

Page 3 (Supporting Information, Fig. S2)

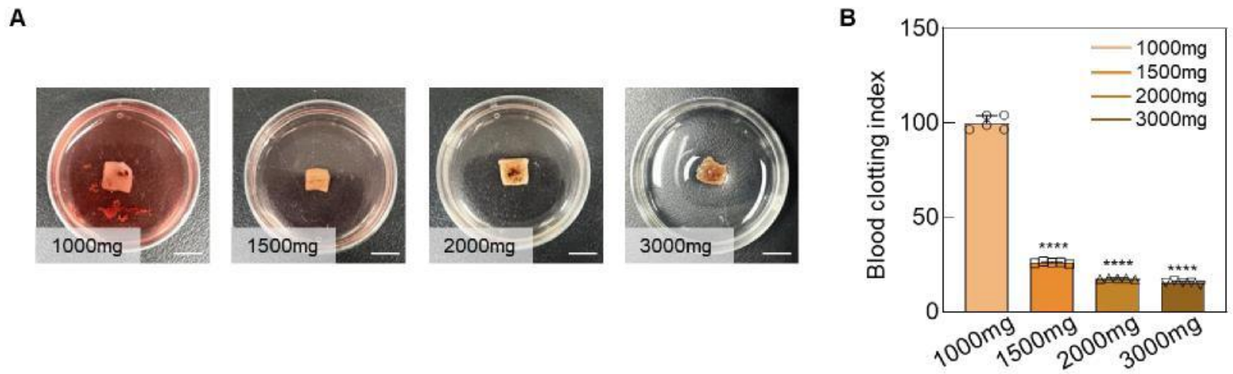

Fig. S2. Optimization of TA concentration for hemostatic performance. (A) Representative photographs of blood coagulation behavior on hydrogels containing increasing TA amounts. (B) Blood clotting index (BCI) as a function of TA dosage, showing saturated coagulation enhancement beyond 2000 mg (n=5). *(*P < 0.05, **P < 0.01, ***P < 0.001, and ****P < 0.0001). ns, not significant.*

Comment 1. Regression, Not Advancement: The authors' approach of using "brief gloved kneading" in ambient air to induce this oxidation is, scientifically speaking, a regression. Unlike established methods where the degree of oxidation is precisely tuned to balance cohesion (self-crosslinking) and adhesion (interfacial bonding), "kneading" is a crude, uncontrolled, and qualitative manual process.

Comment 2. Verdict: This work does not present a conceptual leap. It essentially replicates a well-known chemical mechanism using a less precise, manual method. It represents an incremental formulation study rather than the fundamental advance required for Nature Communications.

Response (Comment 1-2): We thank the reviewer for raising this important point regarding the controllability of catechol oxidation. We agree that oxidation-triggered adhesion systems are ideally tuned to balance cohesion (self-crosslinking) and interfacial bonding, and that purely qualitative manual handling could raise concerns regarding reproducibility. In STAT, however, catechol oxidation is not initiated in an uncontrolled manner. Rather, borax plays a key regulatory role by reversibly complexing catechol groups, thereby suppressing premature oxidation under static conditions and enabling an on-demand activation mechanism (Supplementary Fig. 3). To further address concerns associated with purely manual "gloved kneading," we have implemented a standardized mechanical mixing/kneading procedure at 37 °C using a controlled device, ensuring reproducible activation conditions. A supplementary Fig. 8A demonstrating this device-based mixing protocol has been provided. To rigorously verify controllability, we performed time-dependent oxidation analyses under defined mixing durations (0, 5, 10, 15, and 20 min). Partial oxidation was quantitatively confirmed by UV–vis measurements, together with consistent macroscopic color evolution of the hydrogel corresponding to controlled catechol oxidation (Supplementary Fig. 8B-C). Importantly, rheological and stress-strain measurements further demonstrated that adhesion strength increases systematically with controlled oxidation conditions, supporting a tunable and reproducible activation process (Supplementary Fig. 9A-E). Moreover, to confirm that oxidation does not proceed spontaneously in the absence of mechanical activation, STAT hydrogels stored at room temperature without mixing exhibited no measurable changes in mechanical properties over 48 h (Supplementary Fig. 9F-G). For longer-term stability, samples stored under oxygen-limited conditions (N2-purged flask) for one week maintained mechanical performance comparable to the initial state, indicating that oxidation reaches a stable plateau rather than progressing uncontrollably (Supplementary Fig. 10). Collectively, these additional results demonstrate that STAT provides a controllable and reproducible oxidation-based activation strategy, overcoming the limitations of qualitative manual handling while enabling a clinically practical, reagent-free hemostatic platform for rapid wet adhesion in traumatic environments.

Page 13 (Manuscript)

These results confirm that the hydrogel achieves conformal and covalent adhesion to tissue, preventing mechanical mismatch. Such conformal contact allows the hydrogel to move in harmony with deforming tissues and ensures reliable wound sealing despite frequent patient motion. This robust tissue integration highlights the hydrogel's potential as an effective wound dressing. To address concerns regarding the controllability of catechol oxidation, we performed additional experiments under defined mechanical mixing conditions. As shown in Supplementary Fig. 8A, STAT hydrogels were prepared using a standardized mechanical mixing condition. Representative photographs collected at different mixing times (Supplementary Fig. 8B) show a gradual darkening of the hydrogel, indicating progressive catechol oxidation during mixing. This visual change was

quantitatively analyzed by ImageJ (Supplementary Fig. 8C), confirming a time-dependent increase in color intensity. Importantly, UV–vis spectroscopy further verified the underlying chemical conversion (Supplementary Fig. 8D): the characteristic catechol absorption band at ~280 nm decreased, while a new oxidation-associated band emerged around ~390–400 nm, consistent with catechol-to-o-quinone transformation. These results confirm that catechol oxidation proceeds in a controlled, mixing-time-dependent manner rather than occurring stochastically. Next, we examined how this programmable oxidation translates into mechanical and adhesive
Using a rheometer-based measurement, the adhesion force of STAT hydrogels was quantified as a function of mixing time (Supplementary Fig. 9A). Adhesion increased progressively with mixing duration and reached an optimum at 15 min, which was therefore selected as the standard fabrication condition in this study. Importantly, the mixing-dependent oxidation translated into tunable mechanical and adhesive performance. Adhesion strength and mechanical robustness increased progressively with mixing time, reaching an optimum at 15 min; however, further mixing beyond this point resulted in a pronounced decline in both adhesion and mechanical properties (Supplementary Fig. 9B-D). We attribute this decrease to excessive water loss during prolonged mixing. Consistently, gravimetric analysis revealed substantially greater hydrogel mass reduction at 20 min compared with 15 min, confirming accelerated dehydration under extended mixing conditions (Supplementary Fig. 9E). Finally, STAT hydrogels mixed for 15 min exhibited further mechanical stabilization during room-temperature aging, as evidenced by time-dependent stress-strain behavior and quantified mechanical properties (Supplementary Fig. 9F, G). Collectively, these data demonstrate that STAT oxidation and its resulting adhesive and mechanical performance are highly controllable and reproducible through defined mixing time, directly addressing concerns regarding process variability. In addition, to further confirm that catechol oxidation can be controlled not only during fabrication but also during storage, we examined the effect of limiting oxygen exposure. STAT hydrogels were stored in sealed containers under an N2-purged atmosphere (Supplementary Fig. 10A). After one week, hydrogels stored under ambient conditions exhibited pronounced color darkening, whereas N2-protected samples showed markedly reduced discoloration, indicating suppressed oxidative progression (Supplementary Fig. 10B). Quantitative image analysis confirmed significantly attenuated changes in normalized brightness under oxygen-limited storage (Supplementary Fig. 10C). Importantly, STAT hydrogels preserved under N2 maintained their mechanical and adhesive performance after one week, demonstrating that oxidation-induced property changes can be effectively minimized through controlled storage conditions (Supplementary Fig. 10D).

Page 61 (Manuscript)

Borax-Mediated Control of Catechol Oxidation

To validate borax-mediated control of catechol oxidation, SA-DOPA solutions (1 mg mL^{-1} in PBS) were incubated at 37 °C under three conditions: (i) without borax (static), (ii) with borax (5 mM, static), and (iii) with borax (5 mM) under continuous mixing (800 rpm). After incubation for 15 min in ambient air, UV–vis spectra (230–500 nm) were recorded. Quinone formation was

quantified by extracting the characteristic absorbance values at ~280 nm (catechol) and ~400 nm (o-quinone band), and by calculating the A400/A280 ratio.

Page 61 (Manuscript)

Long-term Storage Stability and Anti-oxidation Evaluation

To investigate the storage stability and prevent oxidative degradation of the STAT hydrogel, an oxygen-free storage system was employed. The hydrogel was placed in a 250 mL multi-neck flask, which was then hermetically sealed with rubber septa. Nitrogen (N_2) gas was purged into the at a flow rate of 5 L/min for 1 min to displace the internal air (approximately 20 volume exchanges) ensuring an inert atmosphere. The flask was then completely sealed to maintain the oxygen-free condition, and the samples were stored for 7 days at room temperature. The stability of the stored hydrogels was evaluated by comparing their properties at day 0 and day 7. The anti-oxidation effect was quantified by measuring the normalized brightness of the hydrogels at different reaction times (0 to 20 min). Additionally, the preservation of adhesive performance after 7 days was assessed through lap shear tests using porcine skin to determine the adhesive strength.

Page 62 (Manuscript)

Standardized Preparation of STAT Hydrogel via Automated Mechanical Kneading

To ensure structural homogeneity and batch-to-batch reproducibility, the STAT hydrogel was prepared using a custom-built automated mechanical kneading system (Fig. A), replacing conventional manual manipulation. The preparation was conducted within a thermal chamber maintained at 37°C to stabilize the crosslinking kinetics. The apparatus was programmed to execute a continuous stretching-and-folding motion following a circular trajectory with a of 6 cm. The system operated at a frequency of 0.3 Hz (3 cycles per 10 seconds), which corresponds to a constant linear kneading speed of approximately 5.66 cm/s. This standardized mechanical stimulation provided uniform shear stress throughout the hydrogel matrix, ensuring a consistent distribution of crosslinks. All samples were processed under these identical spatio-temporal conditions to eliminate operator-dependent variability and to guarantee the reliability of the subsequent physical and adhesive characterizations.

Page 4 (Supporting Information, Fig. S3)

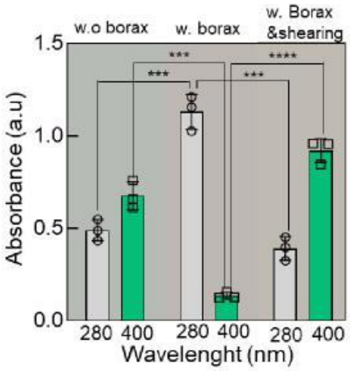

Fig. S3. UV–vis analysis of catechol oxidation in SA-DOPA hydrogels prepared with or without borax (n=3).

Page 9 (Supporting Information, Fig. S8)

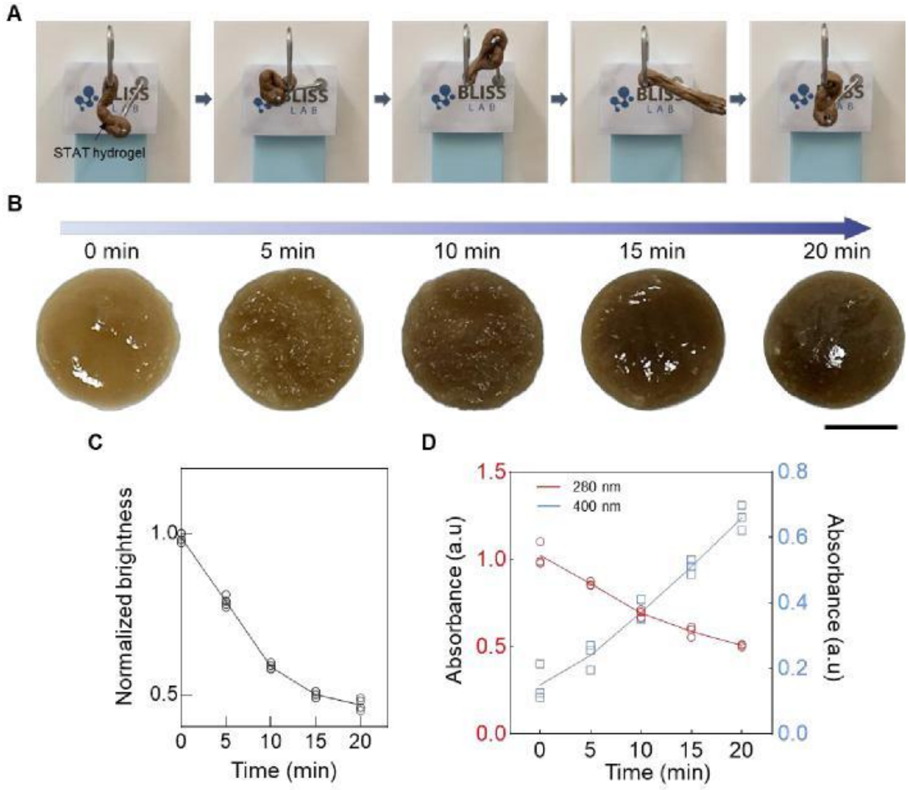

Fig. S8. Time-dependent catechol oxidation during hydrogel mixing. (A) Representative photograph image of mechanical mixing. (B) Representative photographs showing progressive color darkening of the hydrogel as a function of mixing time, indicating gradual oxidation of catechol groups. (C) Quantification of hydrogel color intensity over mixing duration, confirming a time-dependent increase in oxidative conversion (n = 5). (D) UV-vis absorbance spectra of the hydrogel solution collected at different mixing times, demonstrating the emergence of characteristic oxidation-associated peaks, consistent with catechol-to-quinone transformation during mixing (n = 3). *(*P < 0.05, **P < 0.01, ***P < 0.001, and ****P < 0.0001). ns, not significant.*

Page 10 (Supporting Information, Fig. S9)

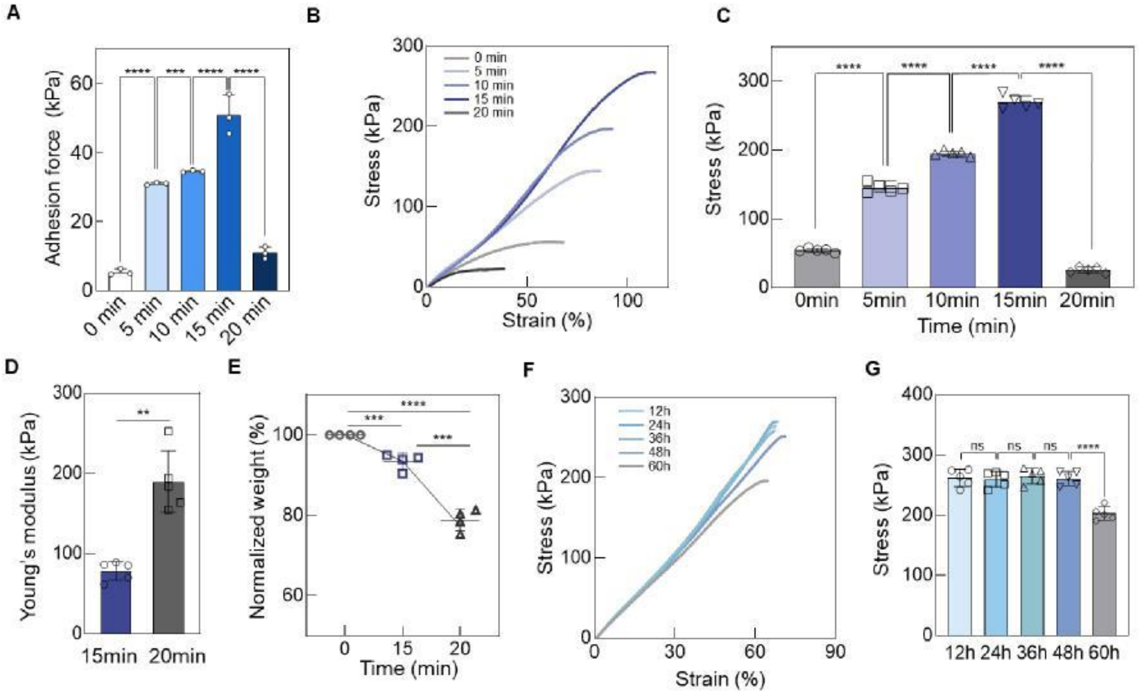

Fig. S9. Oxidation-driven mechanical evolution of STAT hydrogels during mixing and storage . (A) Time-dependent adhesion force of STAT hydrogels measured by rheometry (n=3). (B) Representative stress-strain curves of STAT hydrogels collected at different mixing times (n=5). (C) Quantitative analysis of mechanical performance as a function of mixing time (n=5). (D) Young's modulus of STAT hydrogels measured under varying mixing conditions (n=5). (E) Hydrogel weight change during continuous mixing, revealing gradual mass reduction due to water evaporation (n=4). (F) Stress-strain profiles of STAT hydrogels (15 min mixing) after storage at room temperature for different durations. (G) Quantification of mechanical properties following room-temperature storage (n=5). *(*P < 0.05, **P < 0.01, ***P < 0.001, and ****P < 0.0001). ns, not significant.*

Page 11 (Supporting Information, Fig. S10)

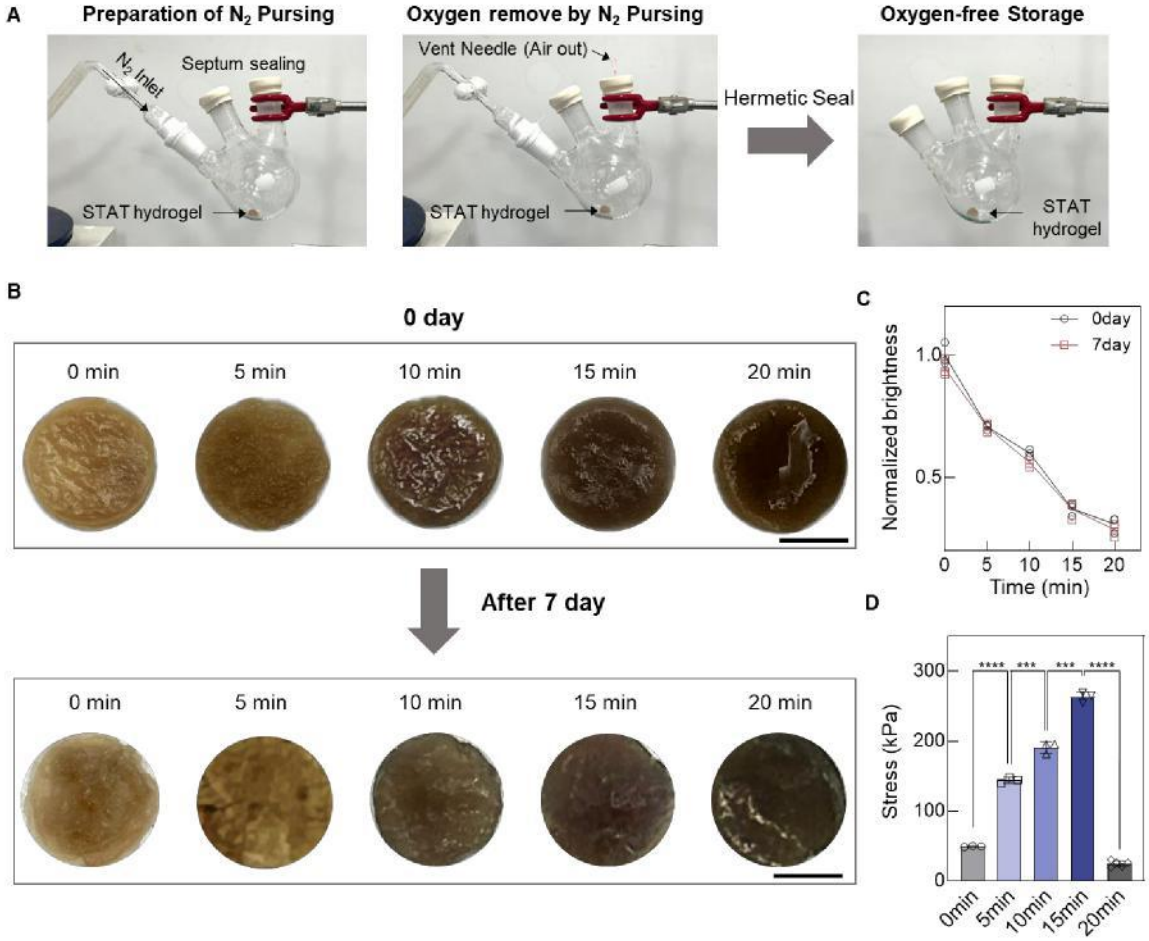

Fig. S10. Suppression of hydrogel oxidation under N2 storage. (A) Photographs showing N2 purging and sealed storage of STAT hydrogels to limit oxygen exposure. (B) Representative images of STAT hydrogels after 1 week of storage under different conditions, revealing reduced color darkening under N2 atmosphere. (C) Quantification of hydrogel color intensity after storage, confirming attenuated oxidation with N2 protection (n=3). (D) Mechanical/adhesion performance measured after 1 week of storage, demonstrating preservation of hydrogel properties under oxygen-limited conditions (n=3). (*P < 0.05, **P < 0.01, ***P < 0.001, and ****P < 0.0001). ns, not significant.

3. Major Scientific Flaws (Critical Issues)

1). The "Black Box" of Kneading-Induced Activation. The reliance on "kneading" undermines the reproducibility of the study.

Comment 1. Uncontrolled Variables: The oxidation rate of catechol is strictly governed by oxygen availability, pH, and radical generation. Manual kneading introduces uncontrollable variables (kneading intensity, duration, glove material, ambient humidity).

Comment 2. Lack of Kinetic Control: The authors fail to provide any kinetic data correlating "kneading energy/time" with the precise concentration of quinone groups. Without this, the synthesis is "art," not science.

Response: We thank the reviewer for emphasizing the importance of reproducible activation kinetics. We agree that catechol oxidation can, in principle, be influenced by variables such as oxygen availability, pH, and environmental conditions. For this reason, STAT activation was not performed through arbitrary manual handling, but under standardized and controlled processing conditions. Specifically, oxidation was triggered using a defined mechanical mixing protocol at fixed temperature (37 °C) and formulation pH, rather than being dependent on glove material or operator-dependent kneading intensity (Supplementary Fig. 8A). Regarding kinetic control, we acknowledge that direct absolute quantification of quinone concentration at soft hydrogel-tissue interfaces remains experimentally challenging, as quinones are transient intermediates that are rapidly consumed through coupling reactions. Instead, we provide reproducible kinetic surrogate measurements that directly track oxidation progression. Time-dependent UV-vis analysis shows a consistent decrease of the catechol absorption band (~280 nm) and emergence of an o-quinone associated feature (~390-400 nm) as a function of defined mixing duration, reaching a plateau within an optimized activation window (~15 min) (Supplementary Fig. 8). Importantly, this oxidation progression correlates quantitatively with adhesion force and mechanical performance, demonstrating that the activation process is tunable and reproducible rather than qualitative (Supplementary Fig. 9). Finally, to further minimize uncontrolled oxidative drift, we demonstrate that oxidation does not continue spontaneously after activation, as samples stored under oxygen-limited (N2-purged) conditions exhibit no additional darkening or property change over one week (Supplementary Fig. 10). Collectively, these results establish that STAT activation is governed by defined processing kinetics and intrinsic formulation regulation, rather than an irreproducible "black-box" manual procedure.

Page 13 (Manuscript)

These results confirm that the hydrogel achieves conformal and covalent adhesion to tissue, preventing mechanical mismatch. Such conformal contact allows the hydrogel to move in harmony with deforming tissues and ensures reliable wound sealing despite frequent patient motion. This robust tissue integration highlights the hydrogel's potential as an effective wound dressing. To address concerns regarding the controllability of catechol oxidation, we performed additional experiments under defined mechanical mixing conditions. As shown in Supplementary Fig. 8A, STAT hydrogels were prepared using a standardized mechanical mixing condition. Representative photographs collected at different mixing times (Supplementary Fig. 8B) show a gradual darkening of the hydrogel, indicating progressive catechol oxidation during mixing. This visual change was quantitatively analyzed by ImageJ (Supplementary Fig. 8C), confirming a time-dependent increase in color intensity. Importantly, UV–vis spectroscopy further verified the underlying chemical conversion (Supplementary Fig. 8D): the characteristic catechol absorption band at ~280 nm decreased, while a new oxidation-associated band emerged around ~390–400 nm, consistent with

catechol-to-o-quinone transformation. These results confirm that catechol oxidation proceeds in a controlled, mixing-time-dependent manner rather than occurring stochastically. Next, we examined how this programmable oxidation translates into mechanical and adhesive Using a rheometer-based measurement, the adhesion force of STAT hydrogels was quantified as a function of mixing time (Supplementary Fig. 9A). Adhesion increased progressively with mixing duration and reached an optimum at 15 min, which was therefore selected as the standard fabrication condition in this study. Importantly, the mixing-dependent oxidation translated into tunable mechanical and adhesive performance. Adhesion strength and mechanical robustness increased progressively with mixing time, reaching an optimum at 15 min; however, further mixing beyond this point resulted in a pronounced decline in both adhesion and mechanical properties (Supplementary Fig. 9B-D). We attribute this decrease to excessive water loss during prolonged mixing. Consistently, gravimetric analysis revealed substantially greater hydrogel mass reduction at 20 min compared with 15 min, confirming accelerated dehydration under extended mixing conditions (Supplementary Fig. 9E). Finally, STAT hydrogels mixed for 15 min exhibited further mechanical stabilization during room-temperature aging, as evidenced by time-dependent stress-strain behavior and quantified mechanical properties (Supplementary Fig. 9F, G). Collectively, these data demonstrate that STAT oxidation and its resulting adhesive and mechanical performance are highly controllable and reproducible through defined mixing time, directly addressing concerns regarding process variability. In addition, to further confirm that catechol oxidation can be controlled not only during fabrication but also during storage, we examined the effect of limiting oxygen exposure. STAT hydrogels were stored in sealed containers under an N2-purged atmosphere (Supplementary Fig. 10A). After one week, hydrogels stored under ambient conditions exhibited pronounced color darkening, whereas N2-protected samples showed markedly reduced discoloration, indicating suppressed oxidative progression (Supplementary Fig. 10B). Quantitative image analysis confirmed significantly attenuated changes in normalized brightness under oxygen-limited storage (Supplementary Fig. 10C). Importantly, STAT hydrogels preserved under N2 maintained their mechanical and adhesive performance after one week, demonstrating that oxidation-induced property changes can be effectively minimized through controlled storage conditions (Supplementary Fig. 10D).

Page 61 (Manuscript)

Borax-Mediated Control of Catechol Oxidation

To validate borax-mediated control of catechol oxidation, SA-DOPA solutions (1 mg mL^{-1} in PBS) were incubated at 37 °C under three conditions: (i) without borax (static), (ii) with borax (5 mM, static), and (iii) with borax (5 mM) under continuous mixing (800 rpm). After incubation for 15 min in ambient air, UV–vis spectra (230–500 nm) were recorded. Quinone formation was quantified by extracting the characteristic absorbance values at ~280 nm (catechol) and ~400 nm (o-quinone band), and by calculating the A400/A280 ratio.

Page 61 (Manuscript)

Long-term Storage Stability and Anti-oxidation Evaluation

To investigate the storage stability and prevent oxidative degradation of the STAT hydrogel, an oxygen-free storage system was employed. The hydrogel was placed in a 250 mL multi-neck flask, which was then hermetically sealed with rubber septa. Nitrogen (N_2) gas was purged into the at a flow rate of 5 L/min for 1 min to displace the internal air (approximately 20 volume exchanges) ensuring an inert atmosphere. The flask was then completely sealed to maintain the oxygen-free condition, and the samples were stored for 7 days at room temperature. The stability of the stored hydrogels was evaluated by comparing their properties at day 0 and day 7. The anti-oxidation effect was quantified by measuring the normalized brightness of the hydrogels at different reaction times (0 to 20 min). Additionally, the preservation of adhesive performance after 7 days was assessed through lap shear tests using porcine skin to determine the adhesive strength.

Page 62 (Manuscript)

Standardized Preparation of STAT Hydrogel via Automated Mechanical Kneading

To ensure structural homogeneity and batch-to-batch reproducibility, the STAT hydrogel was prepared using a custom-built automated mechanical kneading system (Fig. A), replacing conventional manual manipulation. The preparation was conducted within a thermal chamber maintained at 37°C to stabilize the crosslinking kinetics. The apparatus was programmed to execute a continuous stretching-and-folding motion following a circular trajectory with a of 6 cm. The system operated at a frequency of 0.3 Hz (3 cycles per 10 seconds), which corresponds to a constant linear kneading speed of approximately 5.66 cm/s. This standardized mechanical stimulation provided uniform shear stress throughout the hydrogel matrix, ensuring a consistent distribution of crosslinks. All samples were processed under these identical spatio-temporal conditions to eliminate operator-dependent variability and to guarantee the reliability of the subsequent physical and adhesive characterizations.

Page 4 (Supporting Information, Fig. S3)

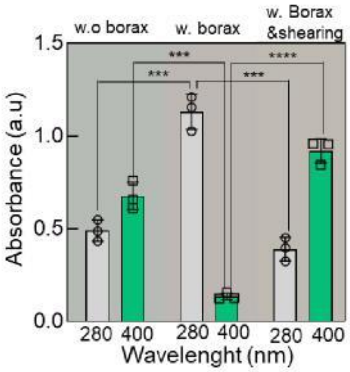

Fig. S3. UV–vis analysis of catechol oxidation in SA-DOPA hydrogels prepared with or without borax (n=3).

Page 9 (Supporting Information, Fig. S8)

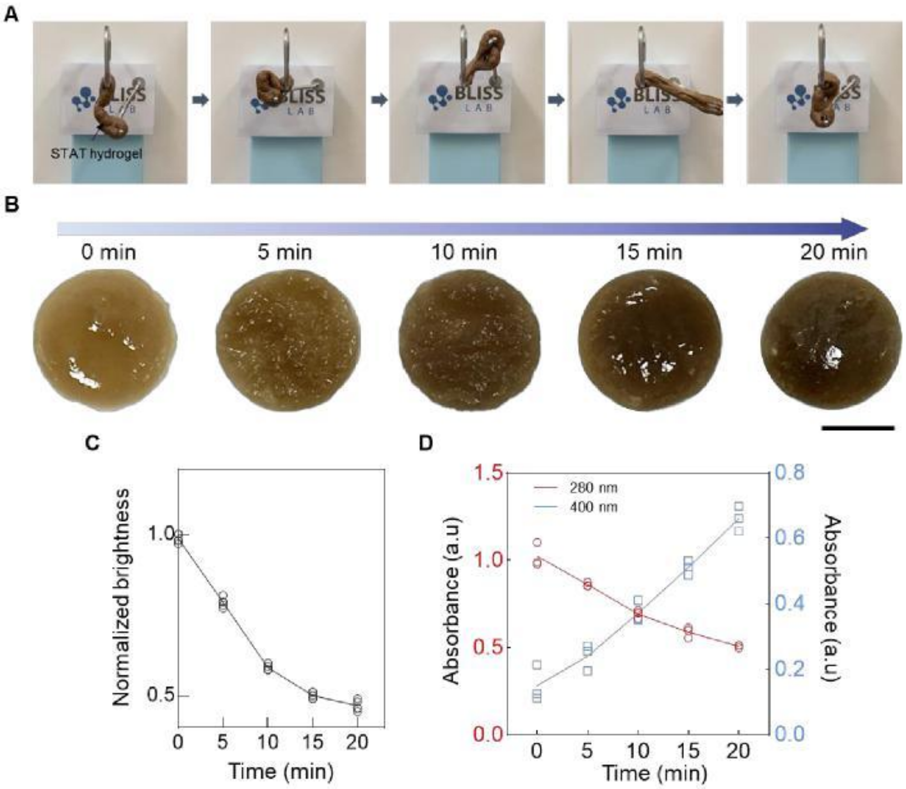

Fig. S8. Time-dependent catechol oxidation during hydrogel mixing. (A) Representative photograph image of mechanical mixing. (B) Representative photographs showing progressive color darkening of the hydrogel as a function of mixing time, indicating gradual oxidation of catechol groups. (C) Quantification of hydrogel color intensity over mixing duration, confirming a time-dependent increase in oxidative conversion (n = 5). (D) UV-vis absorbance spectra of the hydrogel solution collected at different mixing times, demonstrating the emergence of characteristic oxidation-associated peaks, consistent with catechol-to-quinone transformation during mixing (n = 3). *(*P < 0.05, **P < 0.01, ***P < 0.001, and ****P < 0.0001). ns, not significant.*

Page 10 (Supporting Information, Fig. S9)

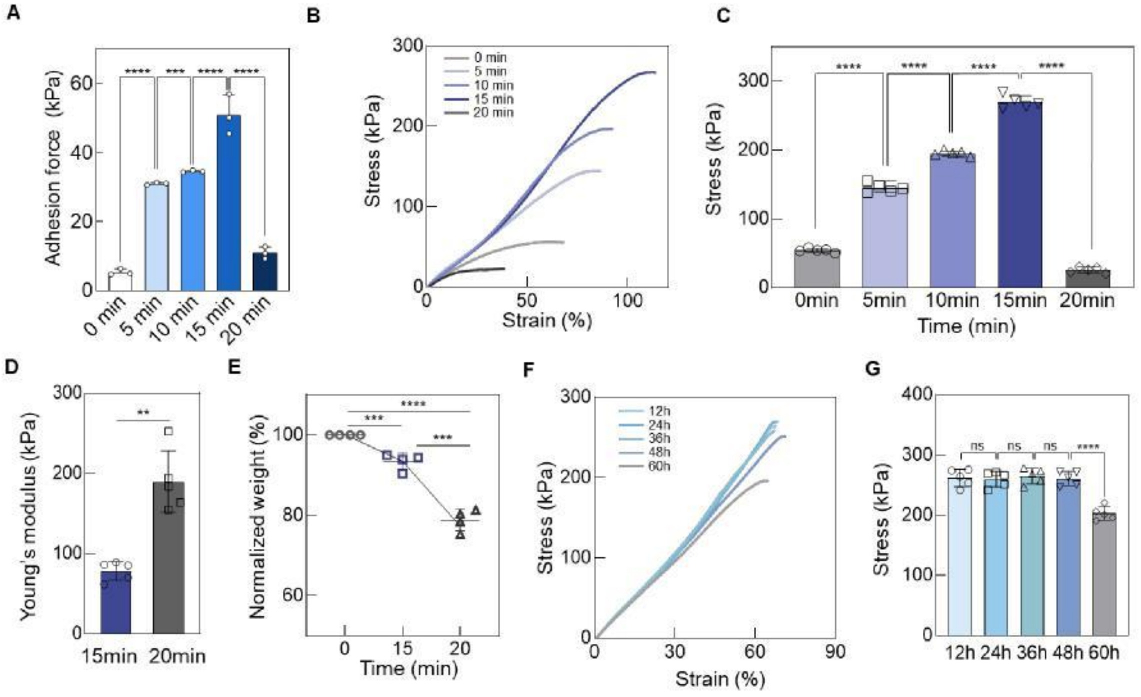

Fig. S9. Oxidation-driven mechanical evolution of STAT hydrogels during mixing and storage. (A) Time-dependent adhesion force of STAT hydrogels measured by rheometry (n=3). (B) Representative stress-strain curves of STAT hydrogels collected at different mixing times (n=5). (C) Quantitative analysis of mechanical performance as a function of mixing time (n=5). (D) Young's modulus of STAT hydrogels measured under varying mixing conditions (n=5). (E) Hydrogel weight change during continuous mixing, revealing gradual mass reduction due to water evaporation (n=4). (F) Stress-strain profiles of STAT hydrogels (15 min mixing) after storage at room temperature for different durations. (G) Quantification of mechanical properties following room-temperature storage (n=5). *(*P < 0.05, **P < 0.01, ***P < 0.001, and ****P < 0.0001). ns, not significant.*

Page 11 (Supporting Information, Fig. S10)

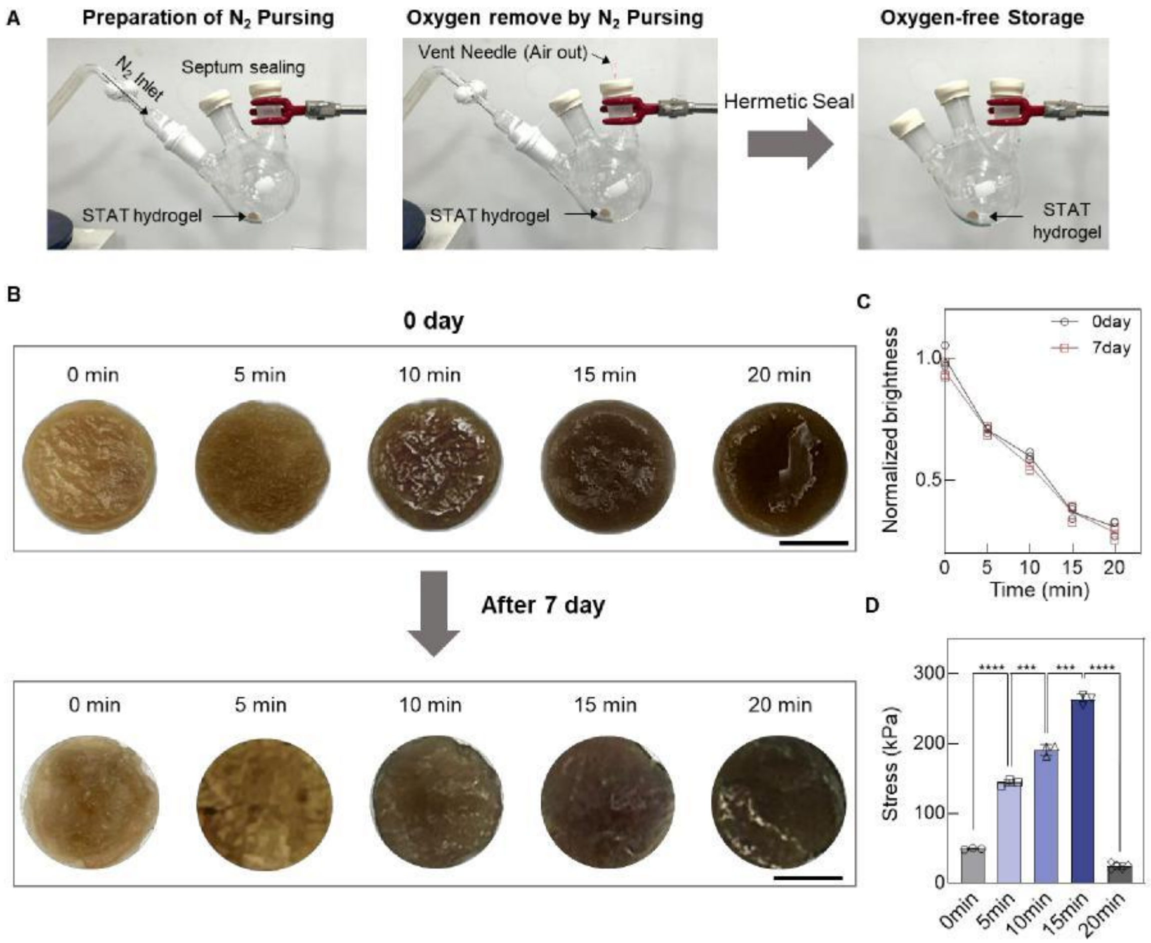

Fig. S10. Suppression of hydrogel oxidation under N2 storage. (A) Photographs showing N2 purging and sealed storage of STAT hydrogels to limit oxygen exposure. (B) Representative images of STAT hydrogels after 1 week of storage under different conditions, revealing reduced color darkening under N2 atmosphere. (C) Quantification of hydrogel color intensity after storage, confirming attenuated oxidation with N2 protection (n=3). (D) Mechanical/adhesion performance measured after 1 week of storage, demonstrating preservation of hydrogel properties under oxygen-limited conditions (n=3). (*P < 0.05, **P < 0.01, ***P < 0.001, and ****P < 0.0001). ns, not significant.

2). Speculative Mechanism of "Covalent" Bonding
The authors attribute the adhesion to covalent bonding (Michael addition) but provide no direct spectroscopic evidence of these bonds at the tissue interface.

Comment 1. Insufficient Proof: Figure 2 only characterizes the bulk hydrogel (UV-vis, FTIR). The macroscopic adhesion tests (Lap shear, Peel test) cannot distinguish between covalent bonding and

strong physical interactions (hydrogen bonding/cation-π) provided by the Tannic Acid and PVA networks.

Comment 2. Requirement: To claim a "covalent" mechanism, surface-specific analysis (e.g., interfacial XPS or using model amine surfaces) is mandatory.

Response (Comment 1-2): We thank the reviewer for raising this important point regarding the mechanistic origin of tissue adhesion. We agree that macroscopic adhesion measurements alone (lap shear and peel tests) cannot unambiguously distinguish covalent bonding from strong noncovalent interactions, such as hydrogen bonding or cation–π interactions contributed by tannic acid and PVA networks. We have therefore revised the manuscript to more carefully phrase the mechanism as being supported by catechol-quinone chemistry rather than claimed as definitive proof. To strengthen mechanistic evidence, we performed additional experiments to evaluate whether nucleophilic tissue functional groups participate in the adhesion process. First, UV-vis spectroscopy confirmed catechol-to-o-quinone conversion upon controlled oxidation, evidenced by attenuation of the catechol band (~280 nm) and emergence of a quinone-associated band (~390– 400 nm) (Fig. 2G). This oxidative transformation is a prerequisite for Michael-type addition reactions. More importantly, we introduced a functional group blocking assay using sulfo-NHS acetate to chemically mask primary amines on porcine skin prior to hydrogel application (Supplementary Fig. 4). Amine blocking significantly reduced the adhesion retention of the oxidized STAT hydrogel after PBS rinsing, indicating that tissue nucleophiles (amines) contribute to the interfacial bonding mechanism. This result provides direct support for quinone-mediated covalent coupling at the tissue-hydrogel interface, beyond bulk characterization alone. While we acknowledge that interfacial XPS would be an ideal surface-specific method, such analysis at soft, hydrated tissue interfaces is technically challenging and often limited by signal complexity. Therefore, we employed the widely accepted model nucleophile-blocking approach as a practical and mechanistically informative alternative. Together, these new data strengthen the conclusion that oxidation-induced quinone formation contributes to enhanced tissue adhesion through covalent interactions, in concert with complementary noncovalent bonding.

Page 12 (Manuscript)

Compared with the pre-oxidized state, the oxidized hydrogel exhibited a distinct decrease in the catechol absorption peak (~280 nm), accompanied by the emergence of a new band around ~390 nm, characteristic of o-quinone formation. Consistent with these spectral changes, optical photographs also revealed a dark brown color after oxidation, hallmark of DOPA oxidation (Fig. 2H). To further support the contribution of quinone-mediated covalent bonding at the tissue interface, we performed a functional group blocking assay using sulfo-NHS acetate to mask primary amines on porcine skin. Amine blocking significantly reduced the adhesion of the oxidized STAT hydrogel after PBS rinsing (Supplementary Fig. 4), indicating that nucleophilic tissue amines participate in the oxidative catechol–quinone coupling mechanism. Adhesion testing on porcine skin demonstrated that pre-oxidized hydrogels adhered but could be removed easily,

whereas oxidized hydrogels exhibited markedly stronger adhesion and resisted detachment (Fig. 2I and Supplementary Movie 1).

Page 5 (Supporting Information, Fig. S4)

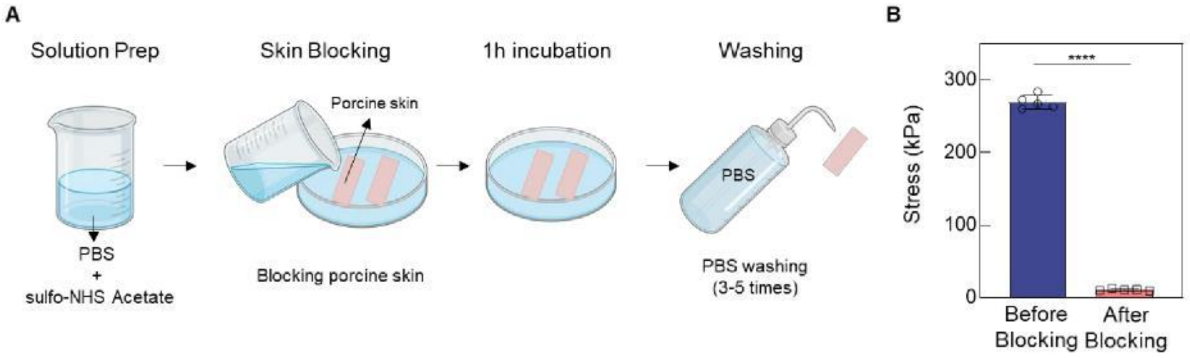

Fig. S4. Tissue amine functional group blocking assay for adhesion mechanism. (A) Schematic of sulfo-NHS acetate pre-treatment to block surface amines on porcine skin prior to hydrogel application. (B) Quantification of STAT adhesion retention after PBS rinsing, showing significantly reduced adhesion on amine-blocked tissue (n=5). *(*P < 0.05, **P < 0.01, ***P < 0.001, and ****P < 0.0001). ns, not significant*

3). Inappropriate Controls in Preclinical Models The translational value is overstated due to weak experimental design in the large animal models.

Comment 1. Weak Comparison: In the severe rabbit hemorrhage models (spleen/liver), the STAT hydrogel is compared only against surgical gauze. Gauze is a passive material known to fail in high-flow bleeding.

Comment 2. Missing Gold Standard: A manuscript of this caliber must compare the proposed material against active clinical standards (e.g., Fibrin Glue, TachoSil). The fact that Fibrin Glue was used in the mild skin model but excluded from the severe bleeding model suggests a selection bias that inflates the perceived efficacy of the hydrogel.

Response (Comment 1-2):

We thank the reviewer for this important comment regarding the need for clinically relevant benchmarking in the severe rabbit hemorrhage models. We fully agree that surgical gauze represents only a passive control and that comparison against active hemostatic standards is essential to rigorously evaluate translational performance under high-flow bleeding conditions. In response, we have performed additional comparative hemostasis experiments in both the rabbit spleen and liver injury models using two FDA-approved clinical standards: fibrin glue as a representative liquid-type sealant and TachoSil as a patch-type hemostatic dressing. These materials are widely used in current surgical practice and therefore provide appropriate gold-standard comparators. As shown in the newly added Supplementary Fig. 17, and Supporting Movie 4, 5, STAT achieved rapid and stable hemostasis with significantly reduced residual blood loss compared with gauze, fibrin glue, and TachoSil. Importantly, these new results confirm that the

hemostatic efficacy of STAT is maintained even under clinically stringent, large-volume bleeding conditions. The revised manuscript now includes full gold-standard comparisons across both mild and severe hemorrhage models, providing a more rigorous and balanced assessment of STAT performance.

Page 22 (Manuscript)

Next, the hemostatic efficacy was assessed in a preclinical large-volume bleeding model using rabbit spleen and liver injury models. For rigorous benchmarking against clinically established hemostatic materials, we additionally performed comparative hemostasis experiments using two FDA-approved standards: fibrin glue as a representative liquid-type sealant and TachoSil as a patch-type hemostatic dressing (Fig. 5E and Supplementary Fig 4, Movie 4, 5). The spleen, which receives approximately 20-25% of the cardiac output and has one of the highest blood flow rates per tissue mass, undergoes massive hemorrhage upon injury. Such bleeding is difficult to control due to its dense microvascular network, high perfusion pressure, and soft parenchymal tissue that resists compression. The liver dissection model mimics extensive organ damage from trauma or surgery, resulting in continuous bleeding over a large area and requiring broad coverage. In both the spleen and liver injury models, applying gauze, fibrin glue, and TachoSil patch failed to achieve reliable hemostasis: even after 3 min of compression, active bleeding persisted from the cut surface (Fig. 5F). In contrast, the STAT hydrogel formed an immediate conformal seal on the wet tissue upon application, rapidly facilitating clot formation at the interface and stopping bleeding within seconds. In the spleen model, incision with a scalpel resulted in persistent surface bleeding that could not be controlled by gauze but was immediately sealed by the STAT hydrogel. Similarly, in the liver dissection model, bleeding continued despite gauze compression. In contrast, the STAT hydrogel adhered firmly to the irregular and large wound site, maintaining a stable hemostatic state throughout the observation period. SEM analysis of the interaction between the bleeding site and the hemostatic material revealed that gauze surfaces retained only a small number of platelets and RBCs (Fig. 5G), whereas the STAT hydrogel surfaces displayed abundant platelets and RBCs tightly bound within a dense fibrin mesh (Fig. 5H). This confirms that the STAT hydrogel induces rapid TA-mediated coagulation and thrombus network formation. Additionally, residual blood volume was compared among bleeding sites treated with gauze, fibrin glue, a TachoSil patch, or the STAT hydrogel (Fig. 5I and Supplementary Fig. 17A). The gauze, fibrin glue, and TachoSil patch -treated group retained substantial amounts of blood, whereas the STAT hydrogel-treated group left only negligible traces. Quantitative analysis demonstrated that residual blood volume in the STAT hydrogel group was markedly reduced compared to the other groups (Fig. 5J and Supplementary Fig. 17B).

Page 53 (Manuscript)

Following approximately one second of uncontrolled bleeding, gauze and the STAT hydrogels were applied to the bleeding wounds, and the amount of blood loss was measured. Blood loss was quantified by measuring the weight of the absorbed materials used during the procedure. After the

experiment, the gauze, fibrin glue (Tisseel, Baxter, USA)., TachoSil patch (Takeda, Japan) and the STAT hydrogels were subjected to SEM (JSM-IT-500HR, JEOL, Japan) imaging to observe their morphology and interaction with the blood components.

Page 18 (Supporting Information, Fig. 17)

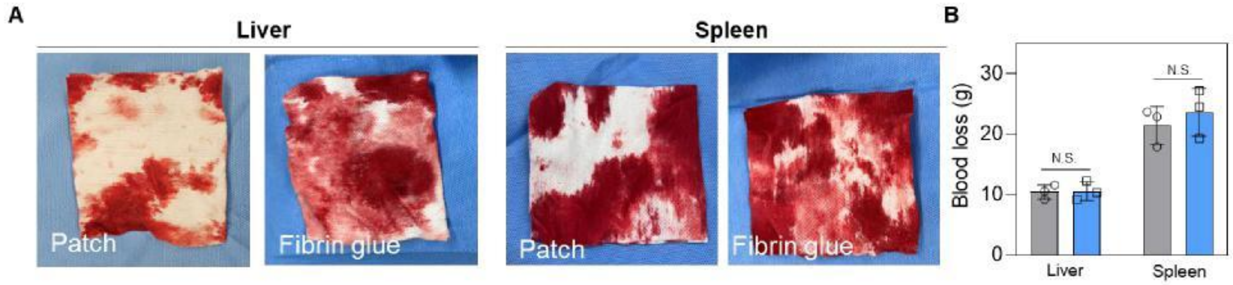

Fig. S17. Hemostatic performance in a rabbit model. (A) Photographs showing blood loss after hemostasis. (**B**) Accumulated blood loss comparisons among liver and spleen treatments (n=3: n is the sample size for each group). *(*P < 0.05, **P < 0.01, ***P < 0.001, and ****P < 0.0001). ns, not significant.*

4. Conclusion

While the multicomponent formulation (DN network) yields tough mechanics, the study's core novelty relies on a mechanism (catechol oxidation) that is already well-understood and is applied here in a scientifically uncontrolled manner ("kneading"). Coupled with the lack of rigorous chemical proof for the interface and weak *in vivo* controls, the manuscript does not meet the standards of novelty and rigor for Nature Communications.

Response: We thank the reviewer for the overall summary and for the constructive critique regarding novelty and rigor. We fully acknowledge that catechol oxidation chemistry is well established in mussel-inspired adhesion and is not claimed here as a new chemical principle. However, we respectfully emphasize that the novelty of STAT does not reside in catechol oxidation in isolation, but in the translational system-level integration of controlled, reagent-free catechol activation within a mechanically reinforced, multifunctional double-network hydrogel designed specifically for severe wet bleeding environments. Importantly, STAT does not rely on scientifically uncontrolled “kneading”. In response to the reviewer’s concern, we have provided new mechanistic data demonstrating that oxidation is intrinsically regulated by borax-mediated catechol complexation, suppressed under static conditions, and reproducibly triggered only within a defined, time-controlled mechanical mixing window using a standardized device (Supplementary Figs. 3, 8-10). Furthermore, we show that oxidation reaches a stable plateau and does not progress uncontrollably during storage under oxygen-limited conditions (Supplementary Fig. 11). Regarding interfacial mechanisms, we have moderated mechanistic language and added new functional group blocking experiments demonstrating that tissue nucleophiles contribute to adhesion, providing additional support for quinone-mediated coupling beyond bulk characterization alone (Supplementary Fig. 4). Finally, to address concerns about translational

benchmarking, we performed additional severe rabbit hemorrhage experiments against widely used clinical gold standards (fibrin glue and TachoSil), eliminating potential selection bias and providing rigorous comparator controls (Supplementary Fig. 17 and Supporting Movie 4, 5). Taken together, these substantial revisions strengthen both the mechanistic rigor and the translational significance of the work. We believe STAT represents a clinically deployable hemostatic platform that uniquely combines instant wet adhesion, toughness, self-healing, and gold-standard validated efficacy under high-flow hemorrhage conditions, which will be of broad interest to the *Nature Communications* readership.

Dear Editors and Referees,

Thank you for your interest and thoughtful comments on our paper, "Quinone-Mediated, Tissue-Adaptive Double-Network Hydrogel for Hemostasis and Wet-Tissue Adhesion", submitted for publication in *Nature Communications.* Please find our explanations and answers below, which hopefully address the issues presented, and they are revised in our newly submitted version of the manuscript. The changes made in the manuscript are highlighted in yellow so that they can be easily traced and will be organized separately as an attachment file. *Characters in red refer to the comments made by reviewers.

Reviewer #1:

This work presents a double-network hydrogel hemostatic adhesive fabricated by incorporating borax and catechol-containing components (tannic acid and dopamine) to facilitate crosslinking, enabling rapid adhesion and hemostasis under mild conditions. The authors systematically characterize the hydrogel's crosslinking structure, mechanical properties, biocompatibility, hemostatic performance, and adhesive capabilities. Overall, this is a well-organized study with substantial experimental data. I recommend acceptance after minor revisions.

Response: We sincerely thank the reviewer for the positive evaluation of our work and for recognizing the systematic characterization and experimental depth of the study. We are grateful for the constructive feedback and have carefully addressed all minor points raised, as detailed below.

Comment 1. Two catechol-containing components—tannic acid and dopamine—are employed in this system. What was the rationale behind using both? Do they serve distinct and complementary roles in the hydrogel formulation?

Response: We thank the reviewer for this insightful comment and apologize for any lack of clarity in our original description. As described in the manuscript, tannic acid (TA) and dopamine were incorporated to fulfill complementary functions within the hydrogel system. TA primarily contributes to rapid hemostasis through its polyphenolic structure, which promotes protein interactions and accelerates clot formation. In contrast, dopamine enhances interfacial adhesion to biological substrates (e.g., skin and wet tissues) via catechol-mediated surface binding. To improve clarity, we have further elaborated this design rationale in the revised manuscript, explicitly emphasizing the distinct yet complementary roles of TA in hemostasis and dopamine in tissue adhesion.

Page 6 (Manuscript)

The upper-right panel of Figure 1A illustrates how one of the main components, TA, interacts with endogenous hemostatic factors such as fibrin and platelets. This interaction concentrates these factors at the bleeding site.[59] This biochemical interaction enables rapid clot formation and hemostasis within seconds. The lower panel depicts how the hydrogel can secure gaping tissue and maintain a moist wound environment, which is known to facilitate natural tissue regeneration and accelerate the wound-healing process. Conventional hydrogel sealants often require chemical activators such as EDC/NHS to form covalent bonds with tissue. This process takes > 1 h and carries a risk of local irritation from residual reagents (Fig. 1B). In contrast, the incorporation of SA-DOPA in our STAT hydrogel enables the instantaneous formation of multiple non-covalent and covalent interactions. These include hydrogen bonding, cation–it interactions, it–it stacking, and Michael addition directly with the tissue surface.[60] Through these mechanisms, the STAT hydrogel achieves robust adhesion within seconds without any pretreatment. This rapid bonding is particularly advantageous in emergency or high-volume bleeding scenarios. Thus, although both TA and SA-DOPA contain catechol moieties, they serve distinct and complementary functions in the STAT hydrogel: TA primarily contributes to rapid hemostasis through interactions with endogenous clotting factors, whereas SA-DOPA provides strong interfacial adhesion to wet tissues through catechol-mediated surface interactions. Figure 1C shows illustrations that the STAT hydrogel exhibits high tensile strength due to its DN structure, where the first rigid network dissipates stress through sacrificial bond breakage and the second ductile network maintains structural integrity.[61]

Comment 2. The manuscript lacks specific details regarding the composition of the STAT hydrogel. What is the proportion of SA-DOPA in the formulation? How was this particular composition determined or optimized?

Response: We thank the reviewer for this important comment. The detailed composition of the STAT hydrogel, including the exact proportion of SA-DOPA relative to PVA and other components, has now been explicitly clarified in the revised Methods section. In the formulation, SA-DOPA (50 mg) was incorporated into the PVA solution (3.64 g in 20 mL DI water), corresponding to approximately 1.37 wt% relative to PVA. As described in the manuscript and Supplementary Fig. S1, the SA-DOPA concentration was systematically optimized. Increasing SA-DOPA content enhanced the mechanical reinforcement of the hydrogel, as evidenced by compressive stress-strain analysis; however, the maximum compressive stress reached a plateau beyond 0.108 M. Therefore, 0.108 M was selected as the minimal concentration achieving saturated mechanical reinforcement while maintaining structural integrity and processability. To improve clarity, we have further elaborated this optimization rationale in the revised manuscript and clearly specified the formulation ratios.

Page 8 (Manuscript)

We used PVA as the backbone polymer. However, PVA hydrogels obtained through the freezing–thawing process are typically brittle and weak, exhibiting poor adhesion to tissue. To enhance

tissue adhesion, SA-DOPA was incorporated into the PVA solution. The SA-DOPA concentration was systematically optimized, and 0.108 M was selected as the minimal level achieving saturated mechanical reinforcement (Supplementary Fig. 1). The flexible alginate chains physically entangle with the PVA network, and the catechol groups of DOPA form multiple hydrogen bonds with hydroxyl groups of PVA.

Page 2 (Supporting Information, Fig. S1)

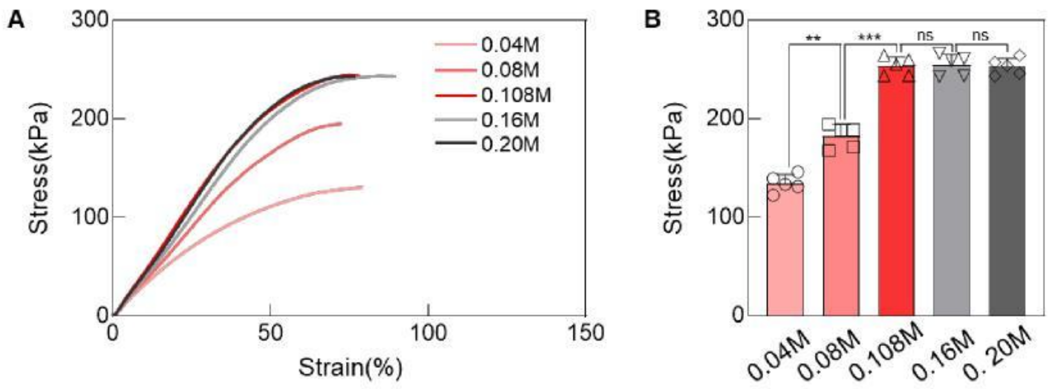

Fig. S1. Optimization of SA-DOPA concentration for STAT hydrogel formation. (A) Representative compressive stress-strain curves of hydrogels prepared with increasing SA-DOPA concentrations. (B) Comparison of maximum compressive stress as a function of SA-DOPA content, showing saturation beyond 0.108 M (n=5: n is the sample size for each group). *(*P < 0.05, **P < 0.01, ***P < 0.001, and ****P < 0.0001). ns, not significant.*

Page 41 (Manuscript)

Fabrication of the STAT Hydrogel

For hydrogel fabrication, a solution of poly(vinyl alcohol) (PVA, 18.18% w/v, 3.64 g in 20 mL DI water) was prepared, and SA–DOPA (50 mg, 0.05 g) was added to the solution. The mixture was stirred at 100 °C for 1 h. Subsequently, 2 mL of borax solution (2 wt%) was added, followed by tannic acid (TA, 9.09% w/v), and the solution was stirred for an additional 2 h. The resulting solution was cast onto a glass plate mold and frozen at −16 °C for 12 h, followed by thawing for 12 h at room temperature. The hydrogel was sequentially dried at 37 °C and then at 100 °C, each for 1 h. To incorporate a PAA network, the dried hydrogel was immersed in an acrylic acid (AA) precursor solution containing acrylic acid (30 wt%), N,N′-bis(acryloyl)cystamine (0.03 wt%), and 2,2′-azobis(2-methylpropionamidine) dihydrochloride (0.15 wt%) in DI water for 2 h. The hydrogel was then heated at 70 °C for 30 min to initiate polymerization. Finally, the hydrogel was washed three times with DI water and soaked overnight in DI water to complete the fabrication process.

Comment 3. The authors attribute the wet adhesion mechanism to the oxidation of catechol to ortho-quinone under ambient conditions, which supposedly enables adhesion without external activation. How was it confirmed that oxidation proceeds specifically to ortho-quinone rather than, for instance, dopamine polymerizing into polydopamine? The UV-vis data presented may not be sufficient to draw this conclusion; additional evidence would strengthen the mechanistic interpretation.

Response: We thank the reviewer for this important mechanistic concern. UV-vis analysis reveals a decrease in the catechol-associated band (~280 nm) together with the emergence of an oxidation-associated band (~400 nm), which is consistent with catechol oxidation to quinone-like species. Furthermore, borax complexation suppresses the oxidation of SA-DOPA, as confirmed by UV-vis spectroscopy, indicating that catechol oxidation within the network is chemically regulated. Upon kneading, progressive color darkening and increased oxidation-associated absorbance are observed, accompanied by enhanced wet-tissue adhesion. This time-dependent correlation suggests that mixing-induced oxidation activates interfacial bonding. While oxidative dopamine polymerization could be considered, it is important to note that our system does not contain free dopamine but alginate-tethered DOPA (SA-DOPA), in which catechol groups are covalently anchored to the polymer backbone. In such polymer-tethered catechol systems, oxidation typically proceeds through quinone intermediates that subsequently participate in intermolecular coupling or interfacial bonding reactions, rather than forming bulk polydopamine deposits. Similar mechanisms have been reported for catechol-functionalized polymers and hydrogels where oxidation of pendant catechol groups leads to quinone-mediated crosslinking or adhesion (Yang et al., *ACS Omega*, **2015**, 1, 126–132; Lee et al., *Science*, **2007**, 318, 426–430). Therefore, the observed color change and adhesion enhancement are more consistent with quinone-mediated oxidation of pendant catechol groups rather than bulk polydopamine formation. To avoid overinterpretation, we have revised the manuscript to moderate the mechanistic claims and to describe the findings as supportive of quinone-mediated adhesion rather than definitive proof of specific ortho-quinone formation.

Page 12 (Manuscript)

The generated oxidized catechol species can undergo covalent bonding through Michael-type addition reactions with nucleophilic groups (e.g., amines, thiols) present in biological tissues, thereby strengthening interfacial adhesion. This oxidative transformation was supported by UV–vis spectroscopy (Fig. 2G). Compared with the pre-oxidized state, the oxidized hydrogel exhibited a decrease in the catechol absorption peak (~280 nm), accompanied by the emergence of a new band around ~390 nm, consistent with the formation of quinone-like species. Consistent with these spectral changes, optical photographs also revealed a dark brown color after oxidation (Fig. 2H), suggesting catechol oxidation within the hydrogel network. To further support the contribution of quinone-mediated covalent bonding at the tissue interface, we performed a functional group blocking assay using sulfo-NHS acetate to mask primary amines on porcine skin.

Page 4 (Supporting Information, Fig. S3)

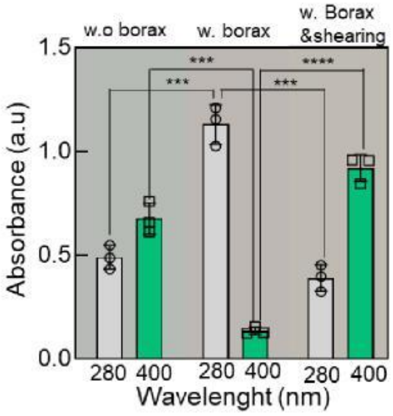

Fig. S3. UV–vis analysis of catechol oxidation in SA-DOPA hydrogels prepared with or without borax (n=3: n is the sample size for each group).

Comment 4. In Figure 3a, how was the PVA hydrogel prepared for comparison? While it is understandable that incorporating SA-DOPA increases crosslinking and enhances toughness, conventional PVA hydrogels can also exhibit relatively high mechanical strength. A more detailed discussion or benchmarking would be helpful.

Response: We thank the reviewer for this valuable comment. To clarify the experimental comparison, we have now explicitly described the preparation method of the control PVA hydrogel in the revised Methods section. The control PVA hydrogel was prepared under identical conditions (polymer concentration, freezing-thawing cycle, and drying process) as the STAT hydrogel, except that SA-DOPA, tannic acid, and borax were not incorporated. This ensures a fair and direct comparison of the mechanical properties. In addition, we have expanded the Discussion section to provide a more detailed benchmarking analysis. While conventional freeze-thaw PVA hydrogels can exhibit moderate mechanical strength due to crystallite formation, they are typically brittle and lack efficient energy dissipation mechanisms (Supplementary Fig. 12). In contrast, the STAT hydrogel benefits from a dual-network architecture and dynamic catechol-mediated interactions, which enhance toughness and mechanical robustness.

Page 42 (Manuscript)

Preparation of PVA Hydrogel

PVA hydrogel was prepared using the freeze–thaw method. Briefly, 4000 mg of PVA was dissolved in 20 mL of DI water under constant stirring at 100 °C for 1 h until a clear homogeneous solution was obtained. The resulting solution was then subjected to a freeze-thaw cycle consisting of freezing at -20 °C for 12 h followed by thawing at room temperature for 4 h. The hydrogel obtained after one freeze–thaw cycle was used for subsequent experiments.

Page 13 (Supporting Information, Fig. S12)

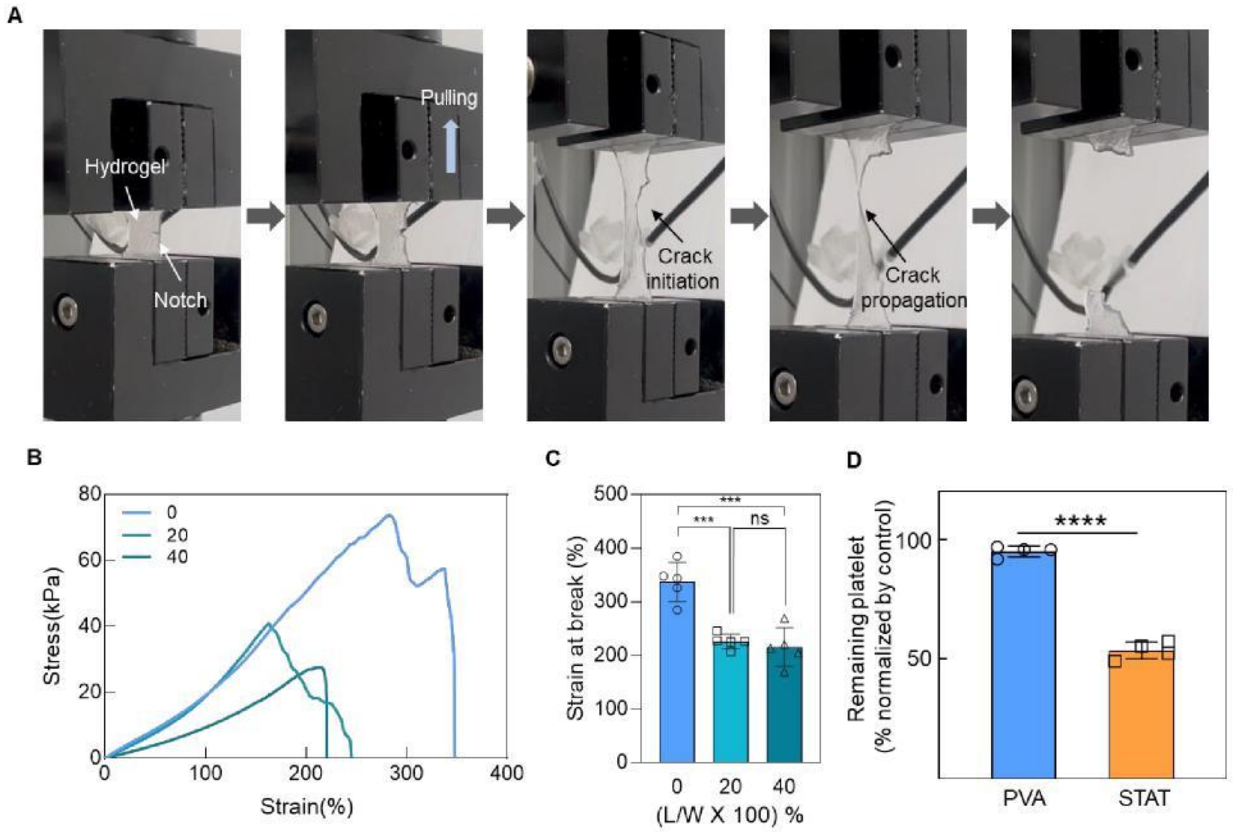

Fig. S12. Notch tolerance and platelet association of STAT hydrogels. (A)Sequential image showing elongation of the notch-introduced PVA hydrogel. (B) Strain at break values of the PVA hydrogel with varying notch lengths. (C) Statistical analysis of the strain-at-break of the notch-introduced PVA hydrogel (n=5: n is the sample size for each group). (D) Remaining platelets in PRP supernatant after 5 min contact with PVA or STAT hydrogels. STAT exhibited significantly enhanced platelet association compared with PVA (n=4: n is the sample size for each group). *(*P < 0.05, **P < 0.01, ***P < 0.001, and ****P < 0.0001). ns, not significant.*

Comment 5. To better highlight the advancements of this work, it would be beneficial to include a comparative analysis with other hemostatic adhesive hydrogels reported in the literature, particularly in terms of adhesion strength, hemostatic efficacy, and mechanical properties.

Response: We thank the reviewer for this constructive suggestion. To better contextualize the advancements of the STAT hydrogel, we have now included a comparative analysis with representative recently reported high-impact hemostatic adhesive hydrogels in the revised manuscript (Table 1). The comparison includes adhesion strength, hemostatic performance, and mechanical properties. This benchmarking analysis highlights that the STAT hydrogel achieves a balanced integration of strong wet-tissue adhesion, rapid hemostatic capability, and robust mechanical properties, demonstrating its competitive performance relative to recently reported state-of-the-art systems.

Page 8 (Manuscript)

While both materials demonstrated comparable biocompatibility, our STAT hydrogel significantly outperformed the fibrin glue in key metrics: mechanical strength, adhesion, antibacterial activity, and hemostatic speed. These results highlight the hydrogel's potential as a next-generation platform for wound treatment. To further contextualize the performance of the STAT hydrogel,
we conducted a comparative analysis with representative recently reported hemostatic adhesive hydrogels based on adhesion strength, hemostatic time, and printability (Table S1).

Page 20 (Supporting Information, Table S1)

Tables

Material	Adhesion Strength [kPa] (shear test)	Hemostatic Time [s]	Printability	Special feature	Refs
STAT hydrogel	273	~ 10 s	O	Quinone-mediated instant wet-tissue adhesion (no EDC/NHS, no external activation)	
DNAgel (DNA hydrogel band-aid)	5.29 ± 0.80	NR (time-to-hemostasis not reported as a single 'seconds' endpoint)	X	NETs-mimicking ultra-swelling hemostatic hydrogel (platelet/RBC enrichment)	1
PVA/TA/PAA hydrogel (TA-incorporated PVA/PAA double-network hydrogel patch)	31	~ 10 s	O	Nonswellable, self-healable tough DN hydrogel with conformal covalent tissue adhesion	2

SSAD-Patch (multilayer hydrophobic/hydrophilic patch)	22.2 ±2.4	NR (reported as sealing within 10 s; not hemostasis-time endpoint)	X	Pressure-triggered wet tissue sealing patch for rapid hemorrhage control	3
ES gel (ε-polylysine + Tetra-PEG-SS injectable adhesive hydrogel)	29.4 ± 7.4	~ 8 s	X	Ultrafast, antibacterial, coagulation-independent hemostatic gel (large-animal/anticoagulated)	4

Table S1. Rapid Hemostasis and Bioadhesive Hydrogel for Wound Healing

Page 21 (Supporting Information)

Reference

1. Ye R*, et al.* Neutrophil extracellular traps-inspired DNA hydrogel for wound hemostatic adjuvant. *Nature Communications* **15**, 5557 (2024).

2. Park J*, et al.* A mechanically resilient and tissue-conformable hydrogel with hemostatic and antibacterial capabilities for wound care. *Advanced Science* **10**, 2303651 (2023).

3. Liu S*, et al.* A nature-derived, hetero-structured, pro-healing bioadhesive patch for high-performance sealing of wet tissues. *Advanced Materials* **36**, 2309774 (2024).

4. Yang Y*, et al.* An injectable hydrogel with ultrahigh burst pressure and innate antibacterial activity for emergency hemostasis and wound repair. *Advanced Materials* **36**, 2404811 (2024).

Comment 6. Were the hydrogels used for performance testing fabricated via 3D printing or conventional mixing/casting methods? If 3D-printed samples were used, do they exhibit comparable performance to their cast counterparts? Clarifying this point would be important given the emphasis on printability.

Response: We thank the reviewer for this important clarification. Hydrogels used for performance testing were fabricated via syringe extrusion from the same hydrogel formulation. In Fig. 6a, syringe extrusion was used to demonstrate the injectability and continuous filament formation of the hydrogel. In Fig. 6e, a low-resolution 3D printer was employed to further confirm structural printability and shape fidelity at a small scale. For the *in vivo* wound healing experiments (Fig.

6g), the hydrogel was applied via syringe extrusion rather than high-resolution 3D printing. This approach was selected because precise architectural patterning is not required for wound coverage, and syringe extrusion ensures uniform material delivery under clinically relevant conditions. Importantly, both syringe-extruded and 3D-printed hydrogels are derived from the same formulation and undergo identical post-gelation processes, resulting in comparable bulk network structures and material properties. Therefore, the performance data reflect the intrinsic material properties rather than fabrication-specific effects. We have clarified this point in the revised manuscript.

Page 24 (Manuscript)
As shown in sequential optical images (**Fig. 6A**), the STAT hydrogel could be smoothly extruded through a standard medical syringe without clogging (ii), forming a continuous filament upon deposition (iii) and maintaining its shape after release (iv). These demonstrations were performed using syringe extrusion to evaluate the injectability and continuous filament formation of the hydrogel under clinically relevant conditions. These results demonstrate that the STAT hydrogel possesses excellent injectability and shape fidelity, supporting the feasibility of syringe-type application for on-demand wound sealing and minimally invasive delivery.

Page 25 (Manuscript)
Under these optimized conditions, the STAT hydrogel could be stably printed into various geometries (e.g., circle, triangle, square, spiral), all of which maintained structural integrity without collapse, demonstrating a high resolution of 400 μm. This experiment was performed to demonstrate the structural printability and shape fidelity of the hydrogel using a low-resolution 3D printing process. High-magnification SEM images further confirmed that the printed constructs exhibited a uniform and smooth surface morphology (Fig. 6E and Supplementary Fig. 9).

Page 26 (Manuscript)
The wound closure capability of the STAT hydrogel was then assessed in a mouse dorsal skin incision model (Fig. 6F). After approximating the wound edges, the hydrogel was injected via syringe to serve as the STAT hydrogel-based sealant. For *in vivo* wound treatment, the hydrogel was applied via syringe extrusion rather than 3D printing, as precise architectural patterning is not required for wound coverage and syringe delivery better reflects clinically relevant application conditions. Unlike conventional sealants that primarily provide mechanical closure, the STAT hydrogel simultaneously adhered to the tissue and retained a moist microenvironment at the wound site.
